# Supplementary material for: Synthesis of vancomycin fluorescent probes that retain antimicrobial activity, identify Gram-positive bacteria, and detect Gram-negative outer membrane damage
Source: Commun Biol. 2023 Apr 14;6:409. doi: 10.1038/s42003-023-04745-x (PMC10102067; doi:10.1038/s42003-023-04745-x)
Supplement: Supplementary file 2 — Supplementary Information [file 42003_2023_4745_MOESM2_ESM.pdf]

Supplementary Information

Synthesis of vancomycin fluorescent probes that retain antimicrobial activity, identify Gram-positive bacteria, and detect Gram-negative outer membrane damage.

Bing Zhang,[1] Wanida Phetsang,[1] M. Rhia L. Stone,[1] Sanjaya Kc,[1] Mark S. Butler,[1] Matthew A Cooper,[1] Alysha G. Elliott[1], Urszula Łapińska,[2,3] Margaritis Voliotis,[2,4] Krasimira Tsaneva-Atanasova,[2,4,5,6] Stefano Pagliara[2,3] and Mark A. T. Blaskovich*

[1]Centre for Superbug Solutions, Institute for Molecular Bioscience, The University of Queensland, Brisbane, Queensland 4072, Australia.

[2]Living Systems Institute, University of Exeter, Stocker Road, Exeter EX4 4QD, UK.

[3]Biosciences, University of Exeter, Stocker Road, Exeter EX4 4Q, UK.

[4]Department of Mathematics, University of Exeter, Stocker Road, Exeter, UK.

[5]EPSRC Hub for Quantitative Modelling in Healthcare, University of Exeter, Exeter, EX4 4QJ, UK.

[6]Department of Bioinformatics and Mathematical Modelling, Institute of Biophysics and Biomedical Engineering, Bulgarian Academy of Sciences, 105 Acad. G. Bonchev Str., 1113 Sofia, Bulgaria.

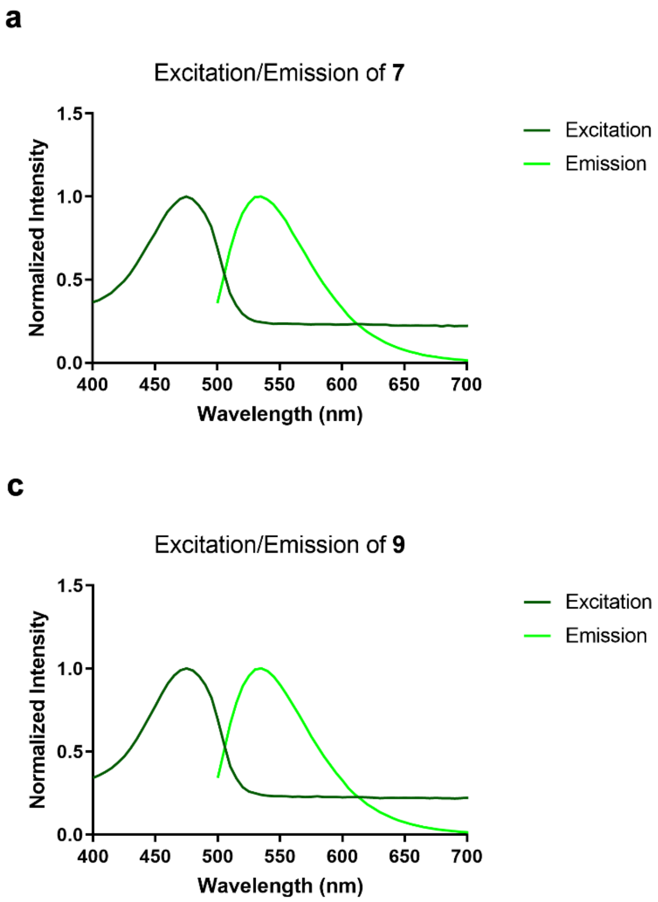

Figure S1. Excitation/Emission of vancomycin probes 7–9. (A) Probe **7; (B)** Probe **8; (C)** Probe **9.**

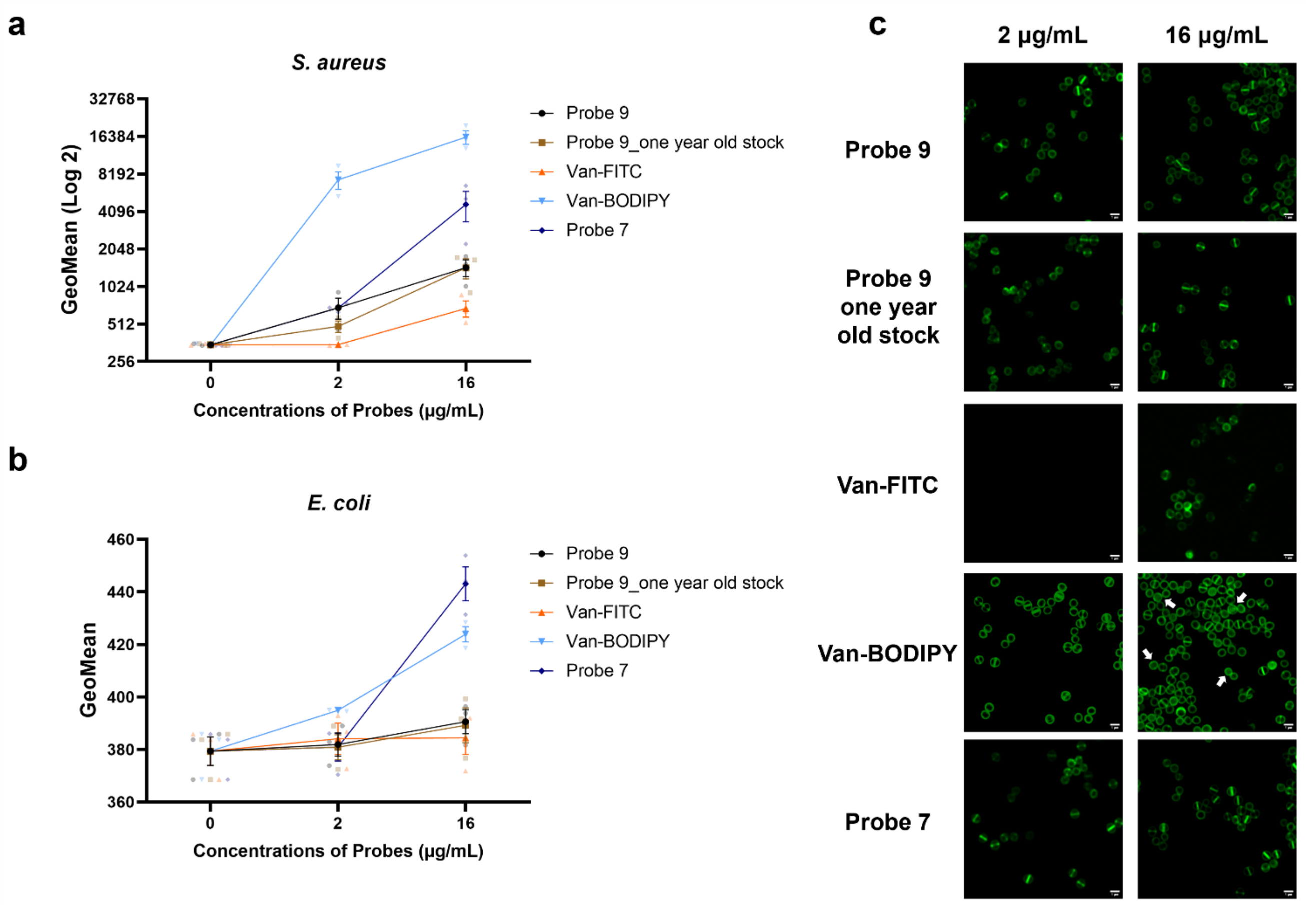

Figure S2. Comparison of probe 7 and probe 9 to commercial fluorescent probes using flow cytometry and microscopy. Labelling of *S. aureus* ATCC 25923 **(A)** and *E. coli* ATCC25922 **(B)** with vancomycin probe **7**, probe **9**, Van-FITC (SBR00028, Sigma-Aldrich), and Van-BODIPY (V34850, Invitrogen™) measured using flow cytometric analysis. Bacteria were labelled with probes for 30 min at 37 °C, the samples were then measured by flow cytometer. The data are presented as the mean ± SEM (*n* = 3). GeoMean: Geometric Mean. **(C)** Airyscan confocal super-resolution imaging of vancomycin probe 7, probe 9, Van-FITC (SBR00028, Sigma-Aldrich), and Van-BODIPY (V34850, Invitrogen™) in *S. aureus* ATCC 25923. Bacteria were stained with probes (2 and 16 µg/mL, Green) for 30 min at 37 °C. Arrows indicate cells with Van-BODIPY entering cytoplasm. Scale bar = 1 µm.

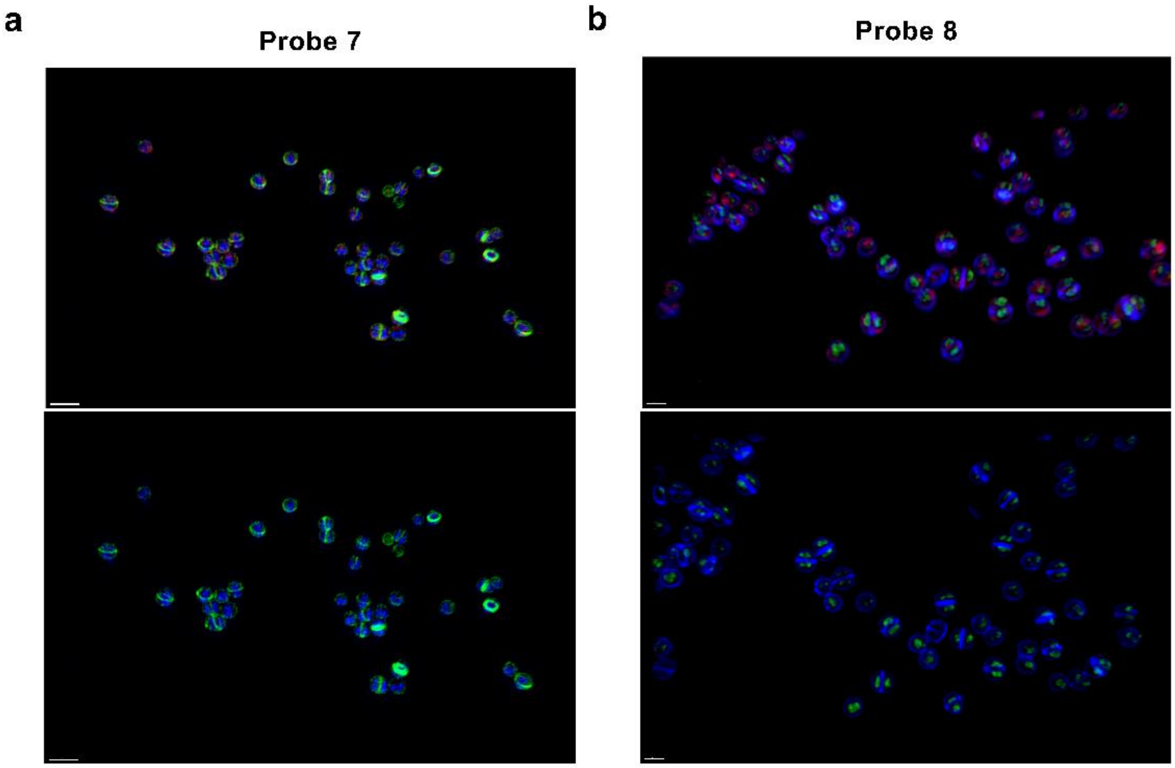

Figure S3. **3D-SIM fluorescence imaging of *Staphylococcus aureus* ATCC 25923. (A)** Green; probe **7**, Red; FM 4-64FX (bacterial membrane), Blue; Hoechst 33342 (nucleic acid); **(B)** Blue; probe **8**, Red; FM 4-64FX (bacterial membrane), Green; SYTO® 21 (nucleic acid); scale bar = 2 µm. Video of *S. aureus* ATCC 700699 labelled with probe **7** is available.

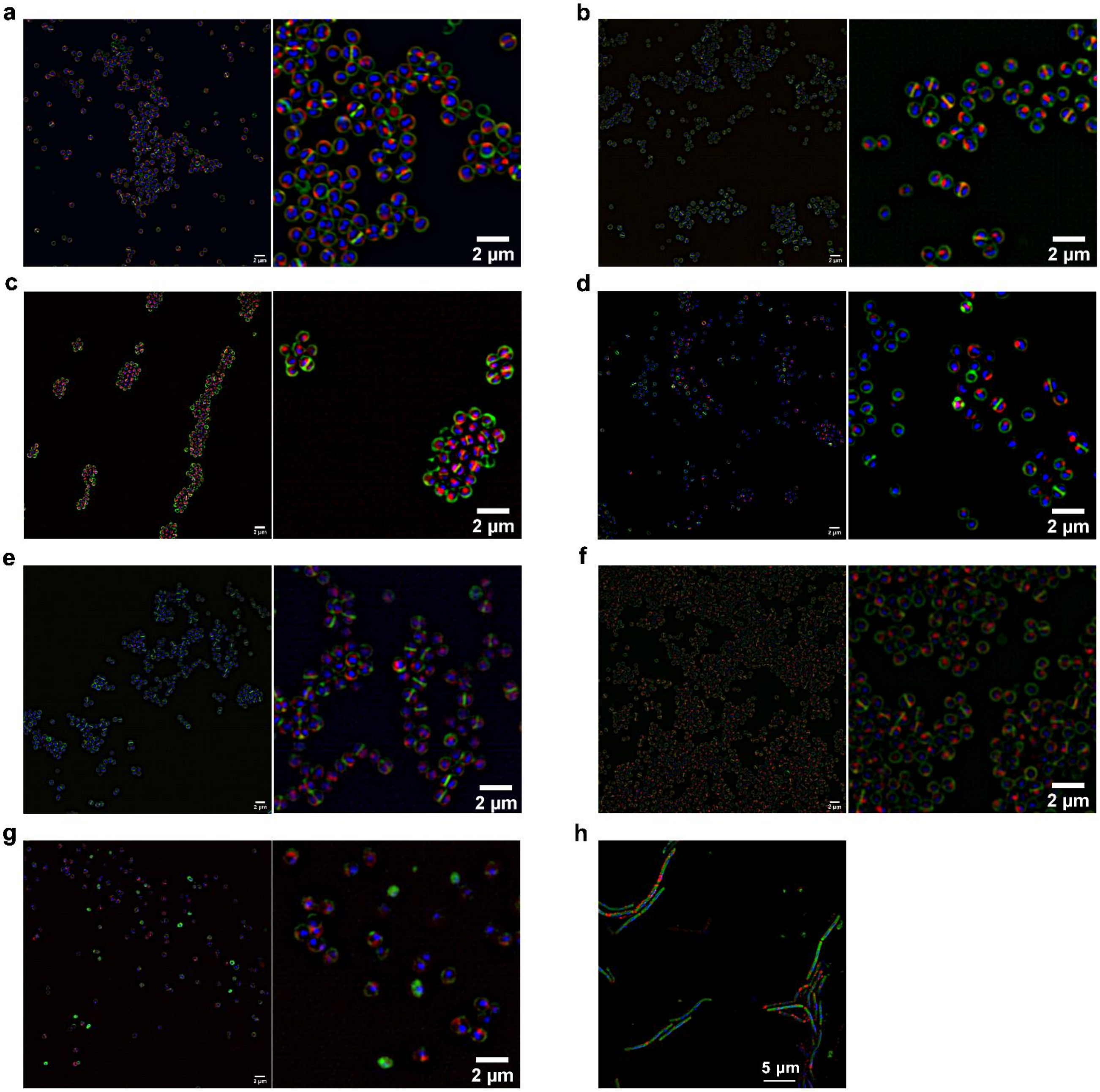

Figure S4. SR-SIM fluorescence imaging of various Gram-positive bacteria. Green: probe **7** (16 µg/mL); Red: FM 4-64FX (bacterial membrane); Blue: Hoechst 33342 (nucleic acid). **(A)** *S. aureus* ATCC 25923**; (B)** MRSA[a] ATCC 43300; **(C)** GISA[a] NRS 1; **(D)** VRSA[a] VRS 4; **(E)** *Staphylococcus epidermidis* ATCC 12228; **(F)** *Streptococcus pneumoniae* ATCC 33400; **(G)** *Enterococcus faecium* ATCC 35667; **(H)** *Bacillus subtilis* (ATCC 6633). **(A-G)** scale bar = 2 µm**; (H)** scale bar = **5** µm**.** [a]abbreviations detailed in full in **Table S4**.

NMR and MSMS spectrum for fluorescent vancomycin

NMR

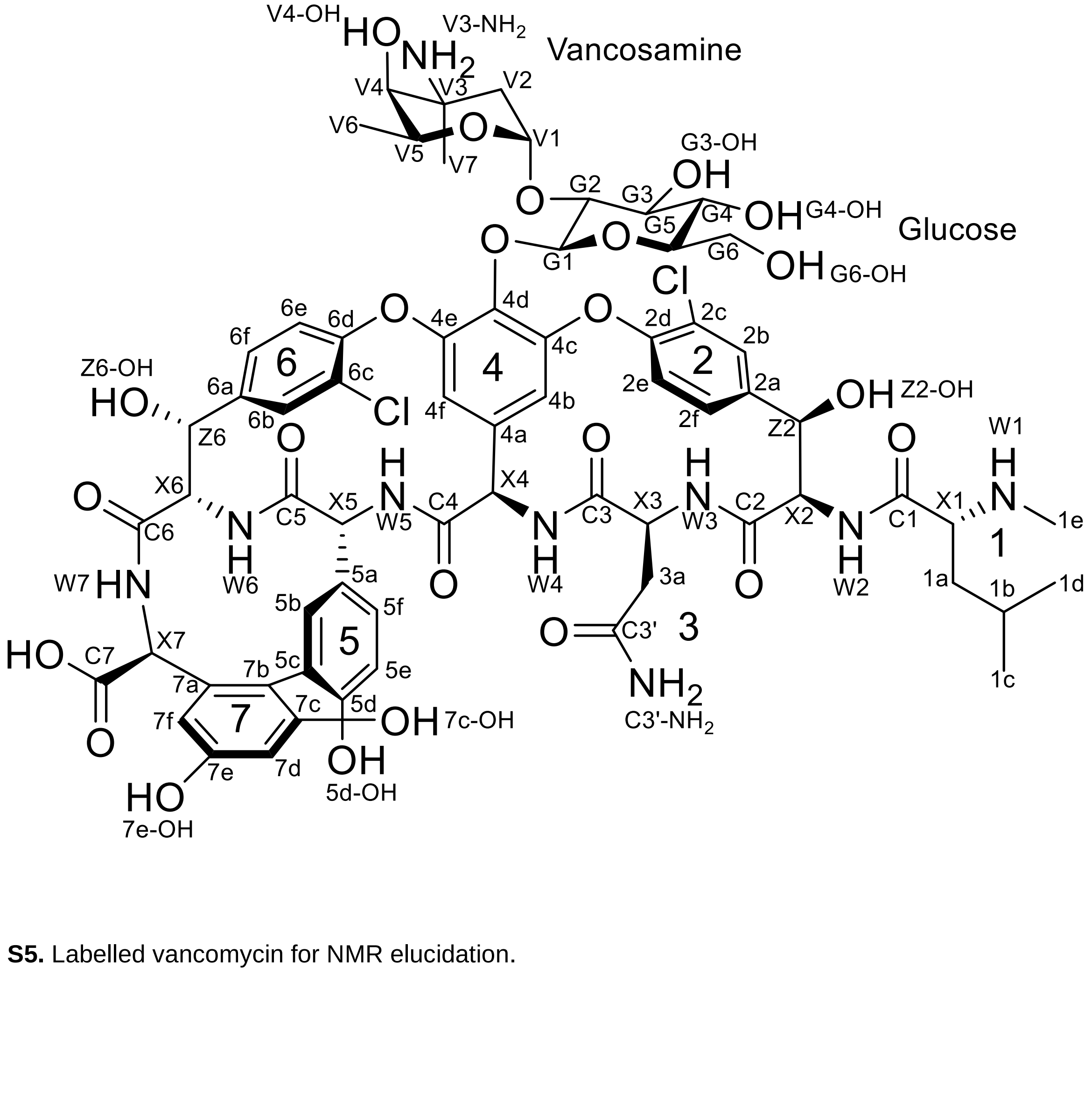

Figure S5. Labelled vancomycin for NMR elucidation.

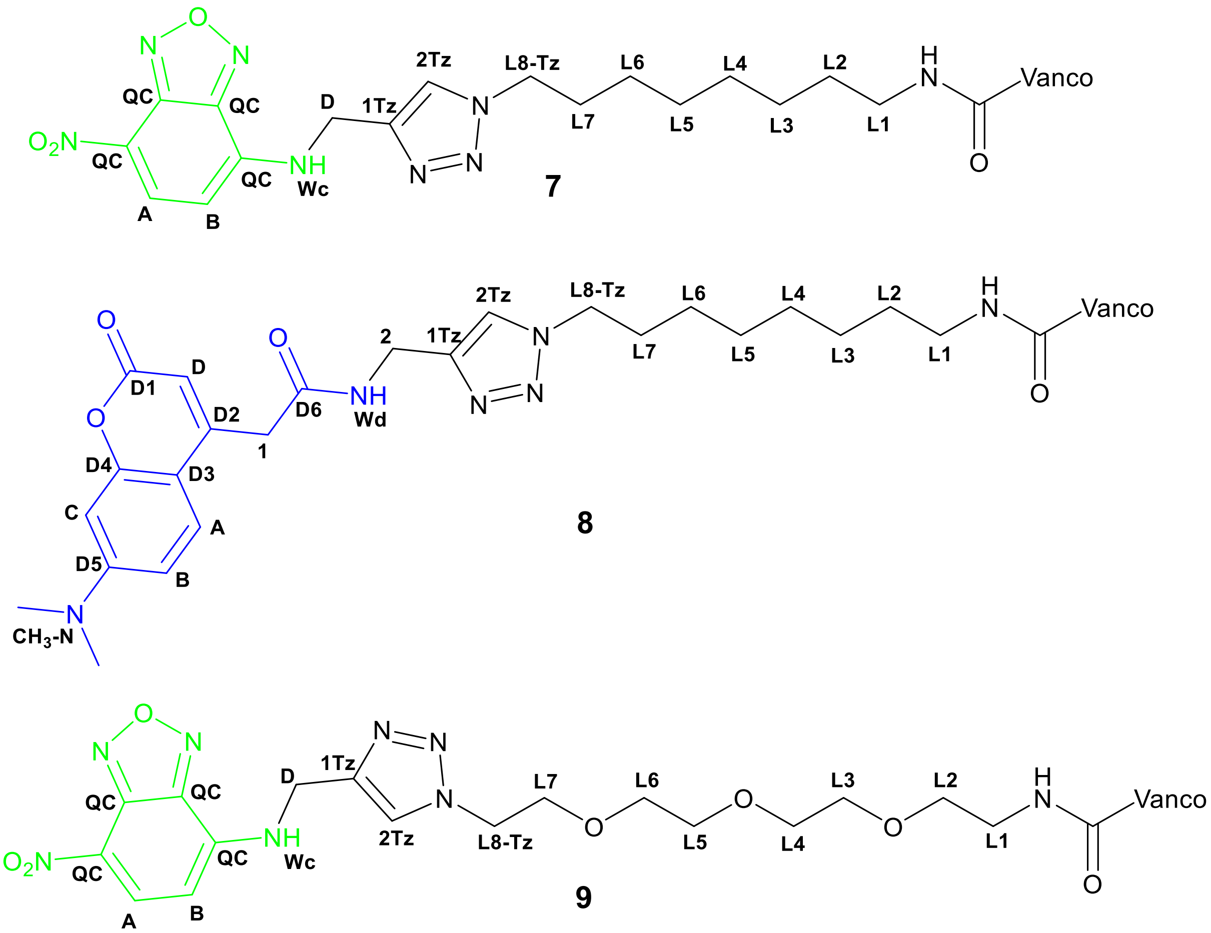

Figure S6. Labelled vancomycin probes for NMR elucidation.

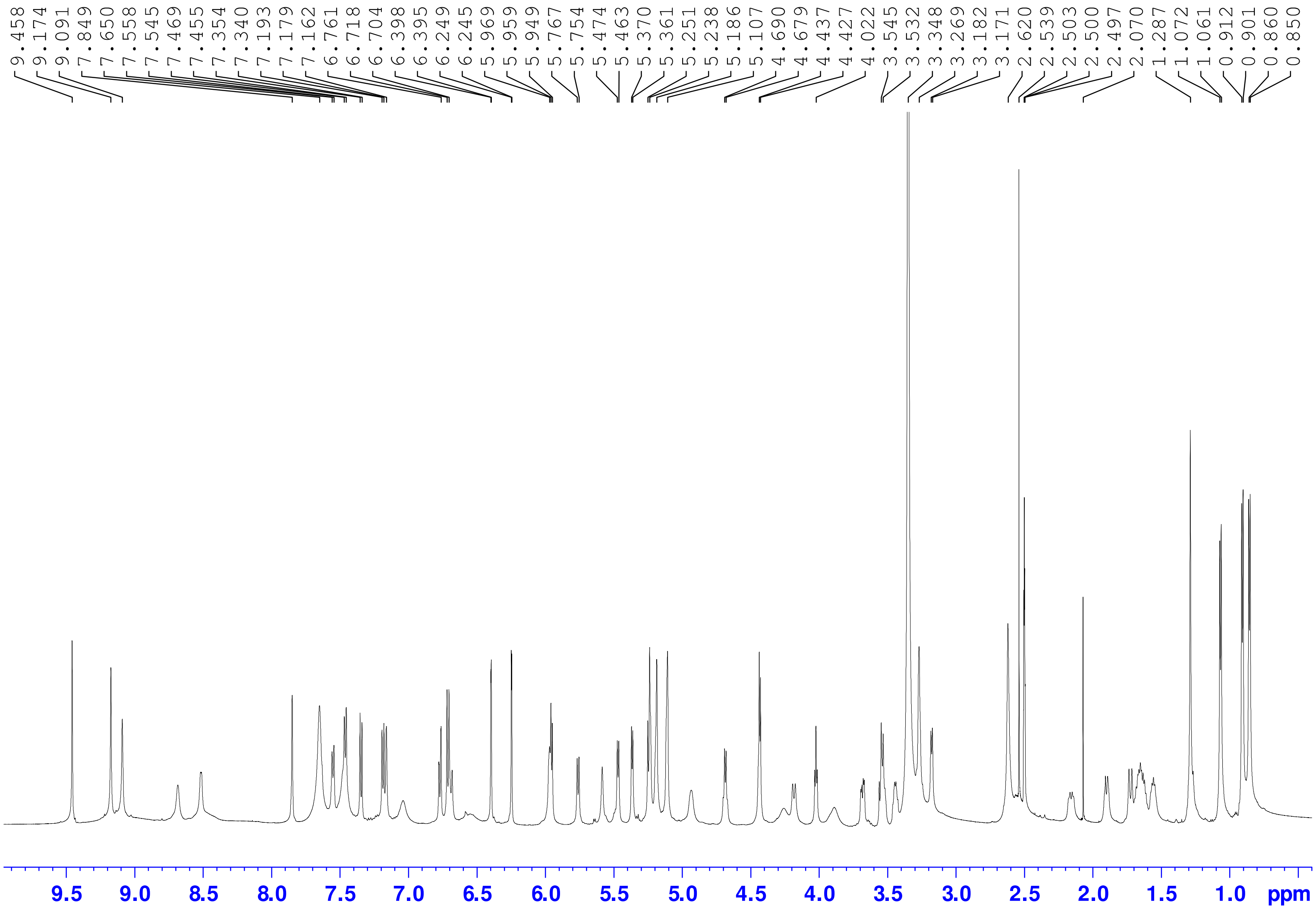

Figure S7. ^{1}H NMR (600 MHz, DMSO-d_6) of vancomycin TFA salt form **1**.

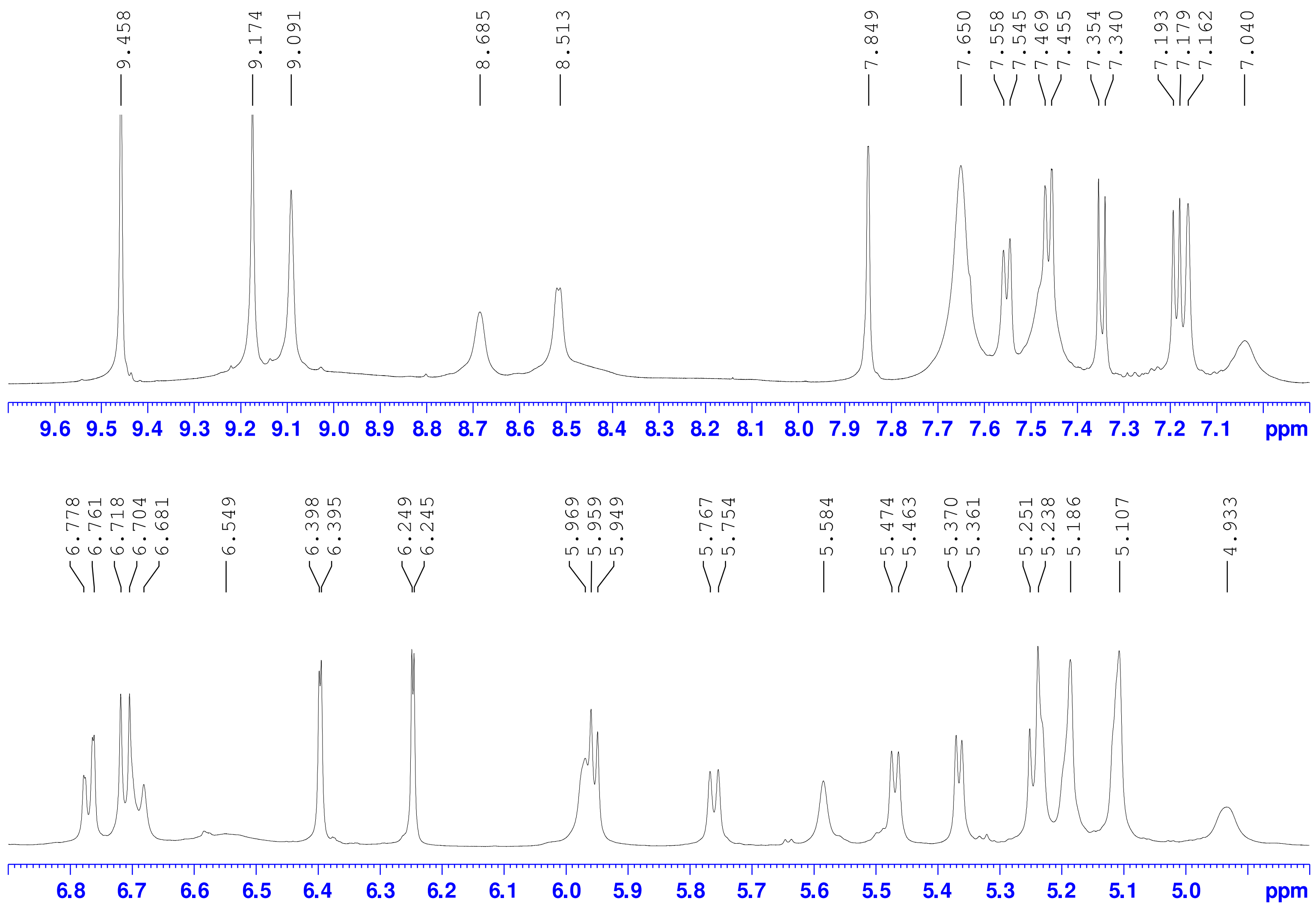

Figure S8. ^{1}H NMR (600 MHz, DMSO-d_6) of vancomycin TFA salt form **1**.

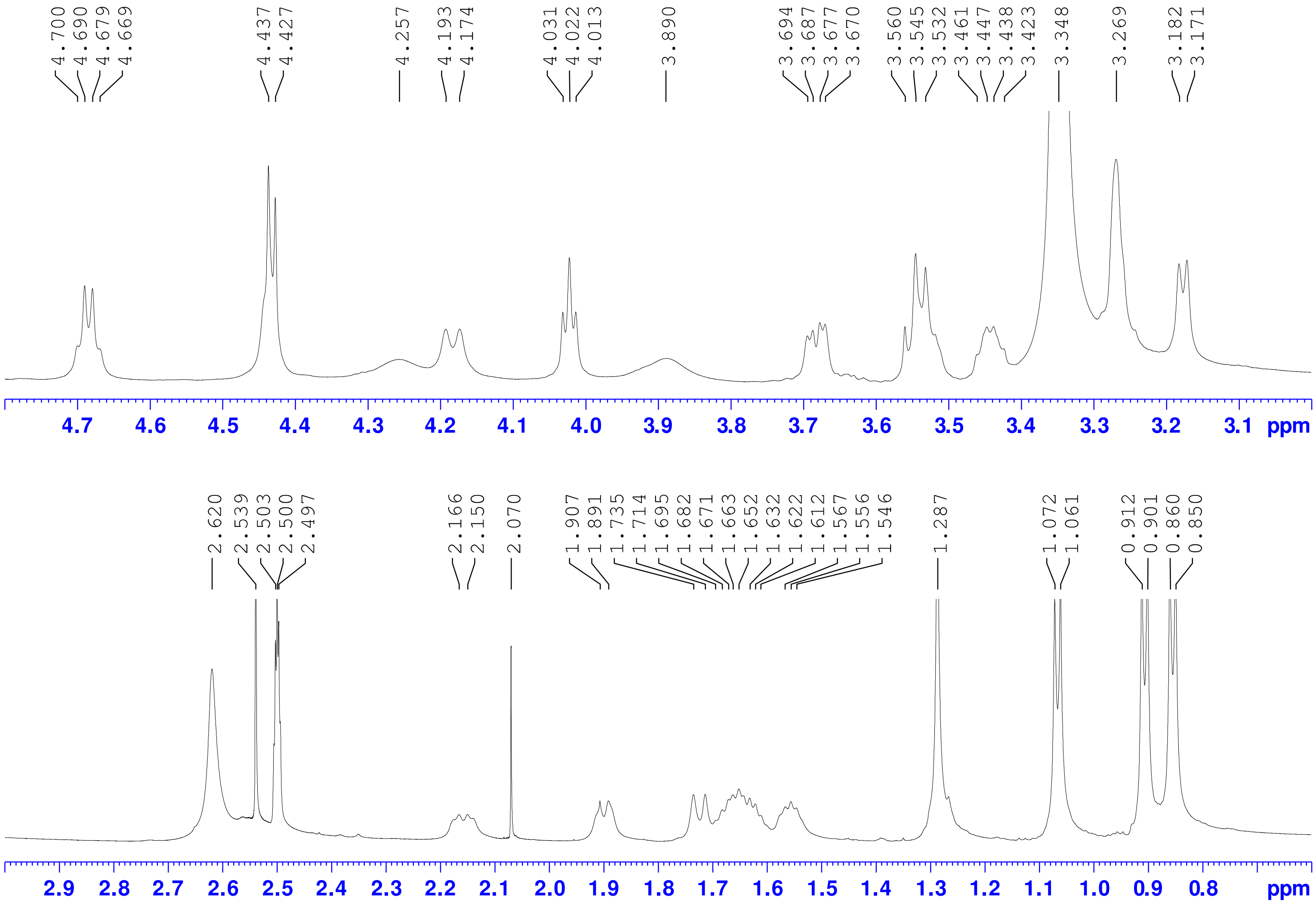

Figure S9. ^{1}H NMR (600 MHz, DMSO-d_6) of vancomycin TFA salt form **1**.

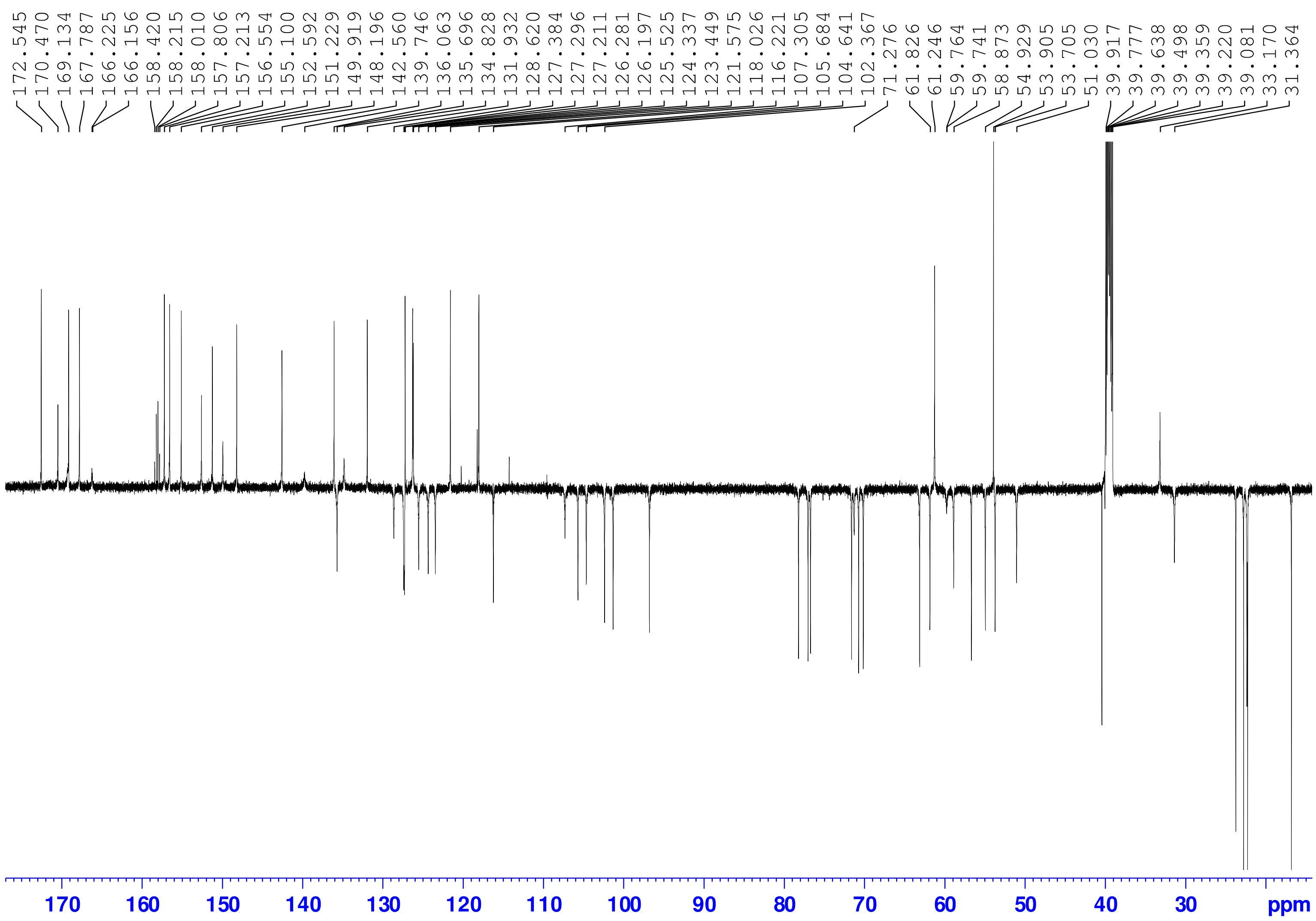

Figure S10. JMOD NMR (150 MHz, DMSO-d_6) of vancomycin TFA salt form **1**.

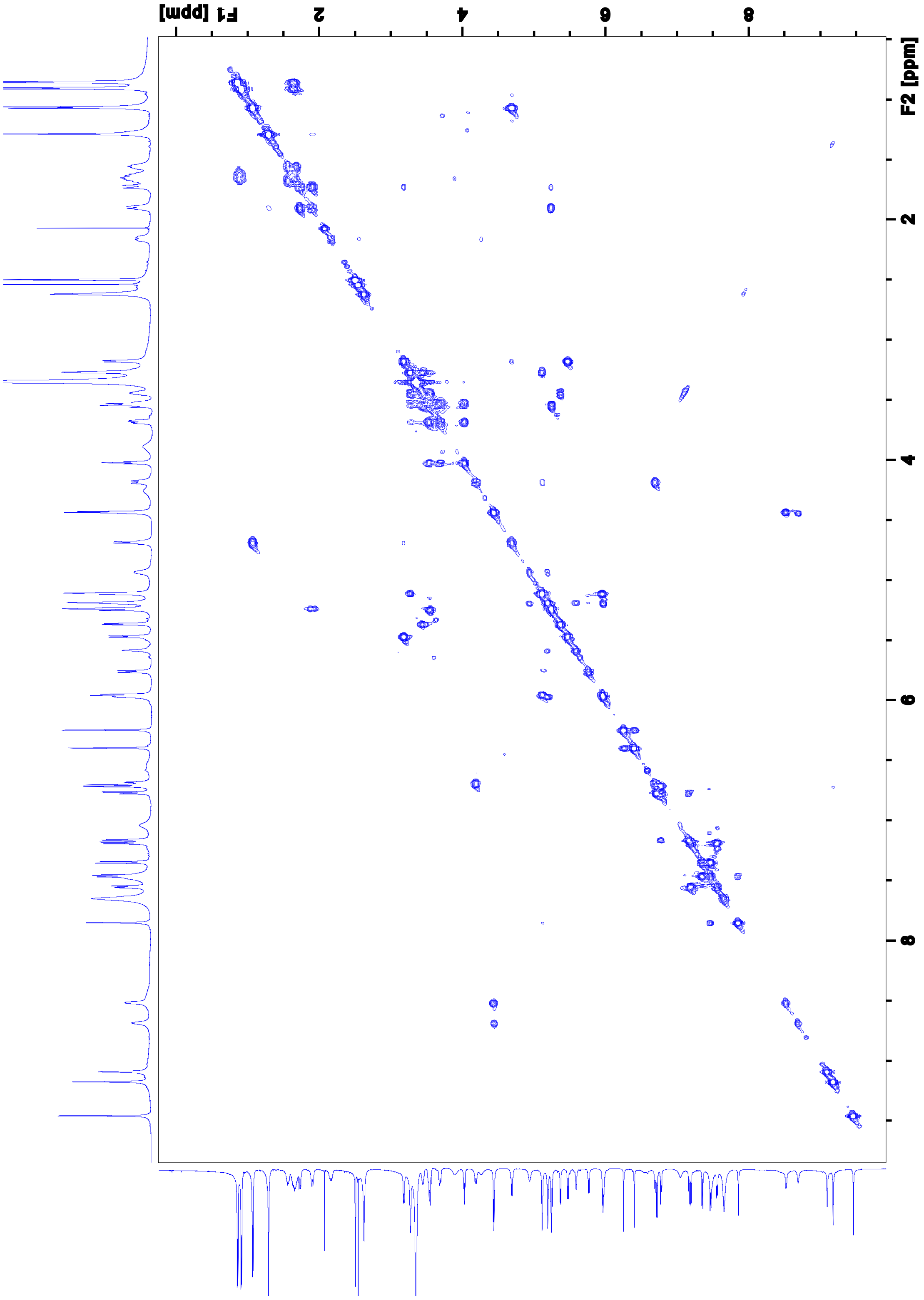

Figure S11. gCOSY spectra (DMSO-d_6) of vancomycin TFA salt form **1**.

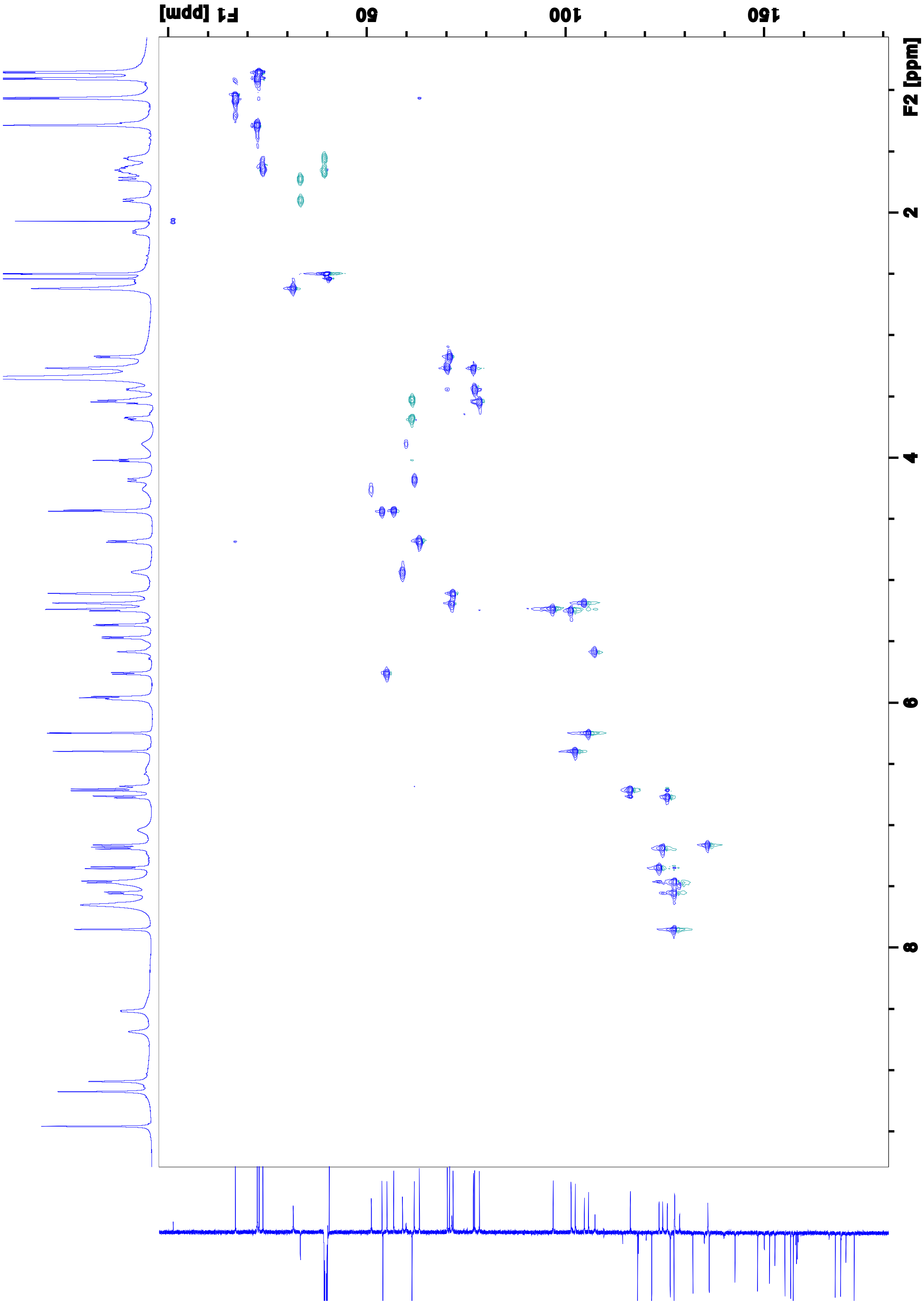

Figure S12. edHSQC spectra (DMSO-d_6) of vancomycin TFA salt form **1**.

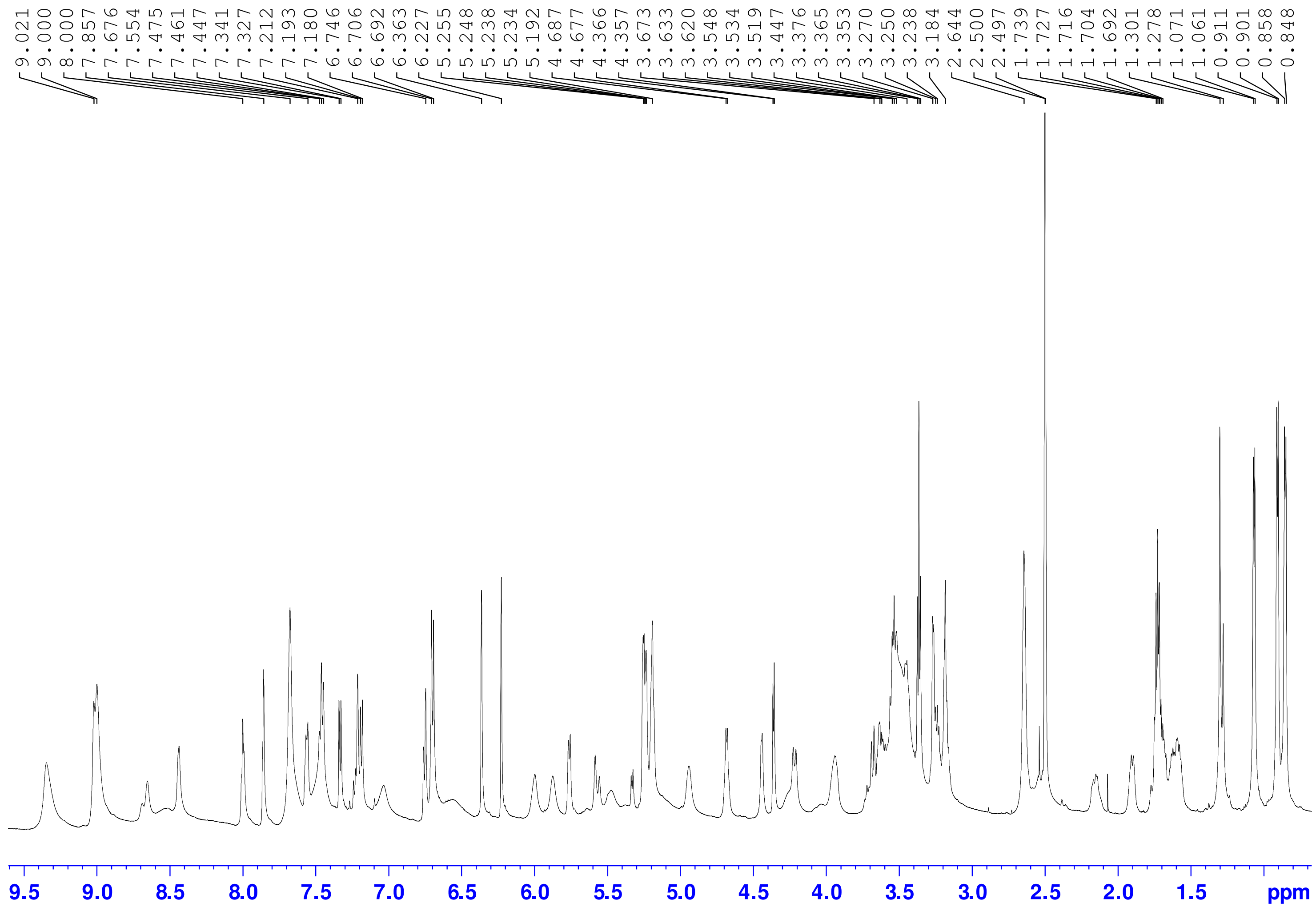

Figure S13. ^{1}H NMR (600 MHz, DMSO-d_6) of vanco-3C-N_3 **2**.

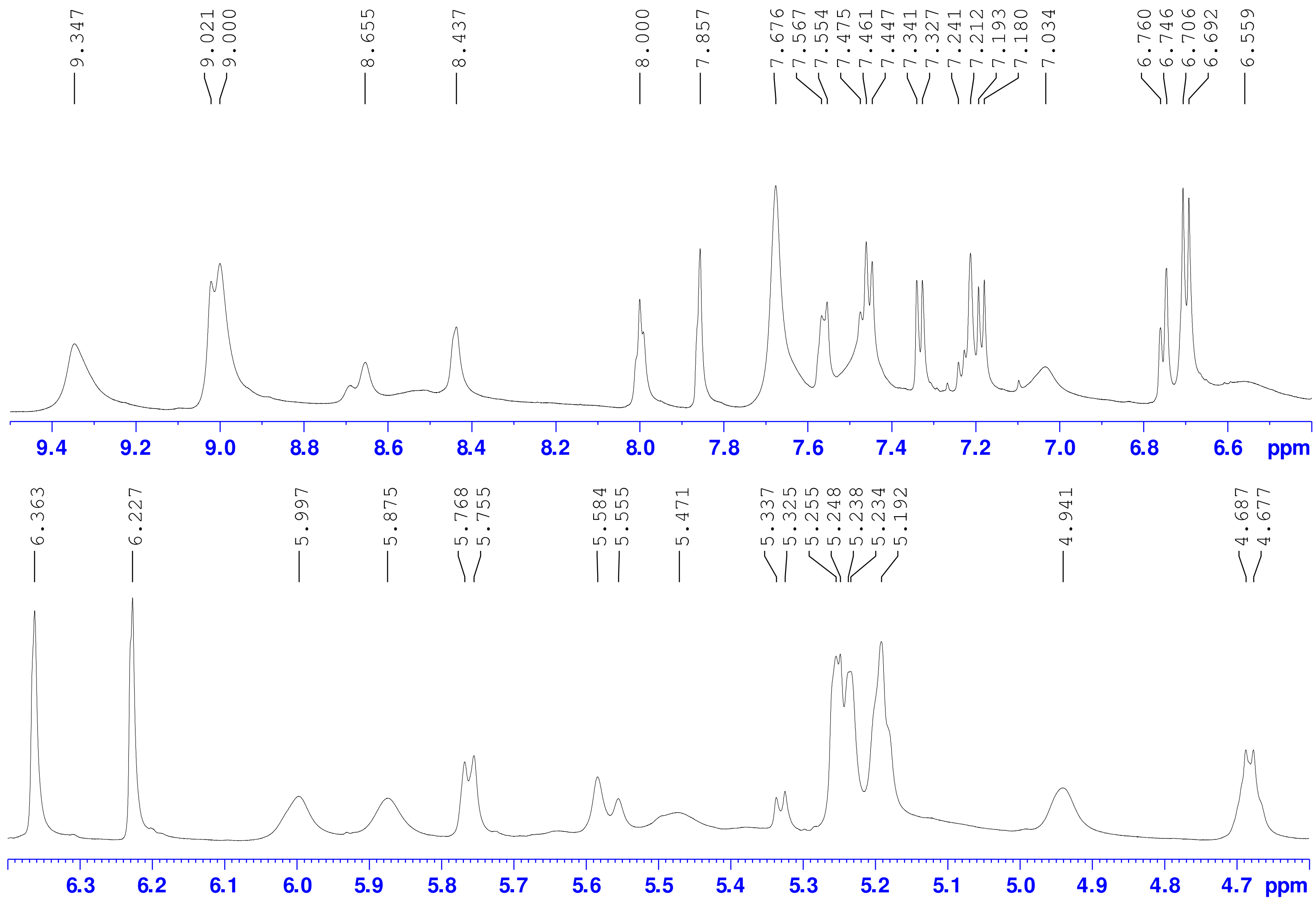

Figure S14. ^{1}H NMR (600 MHz, DMSO-d_6) of vanco-3C-N_3 **2**.

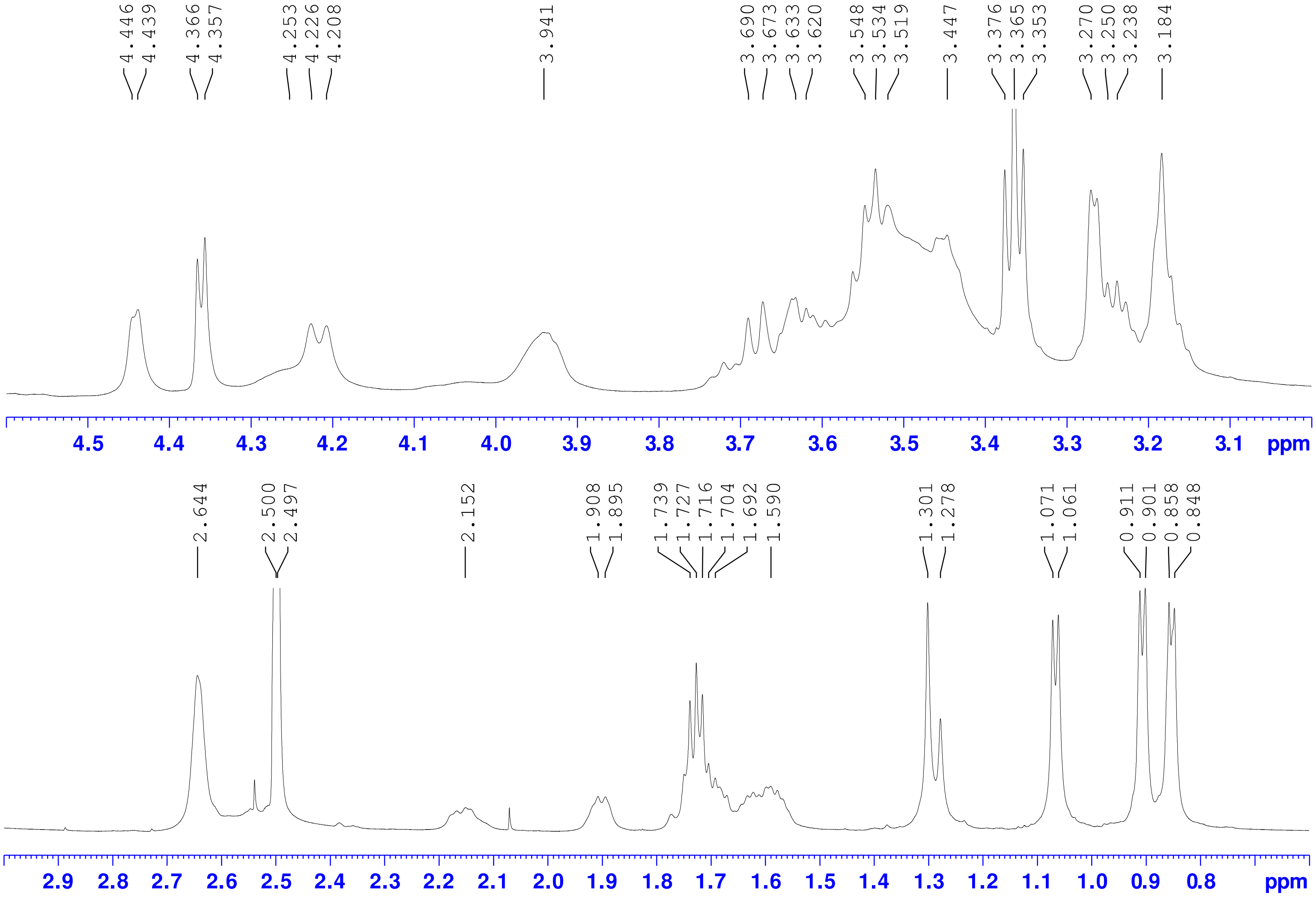

Figure S15. ^{1}H NMR (600 MHz, DMSO-d_6) of vanco-3C-N_3 **2**.

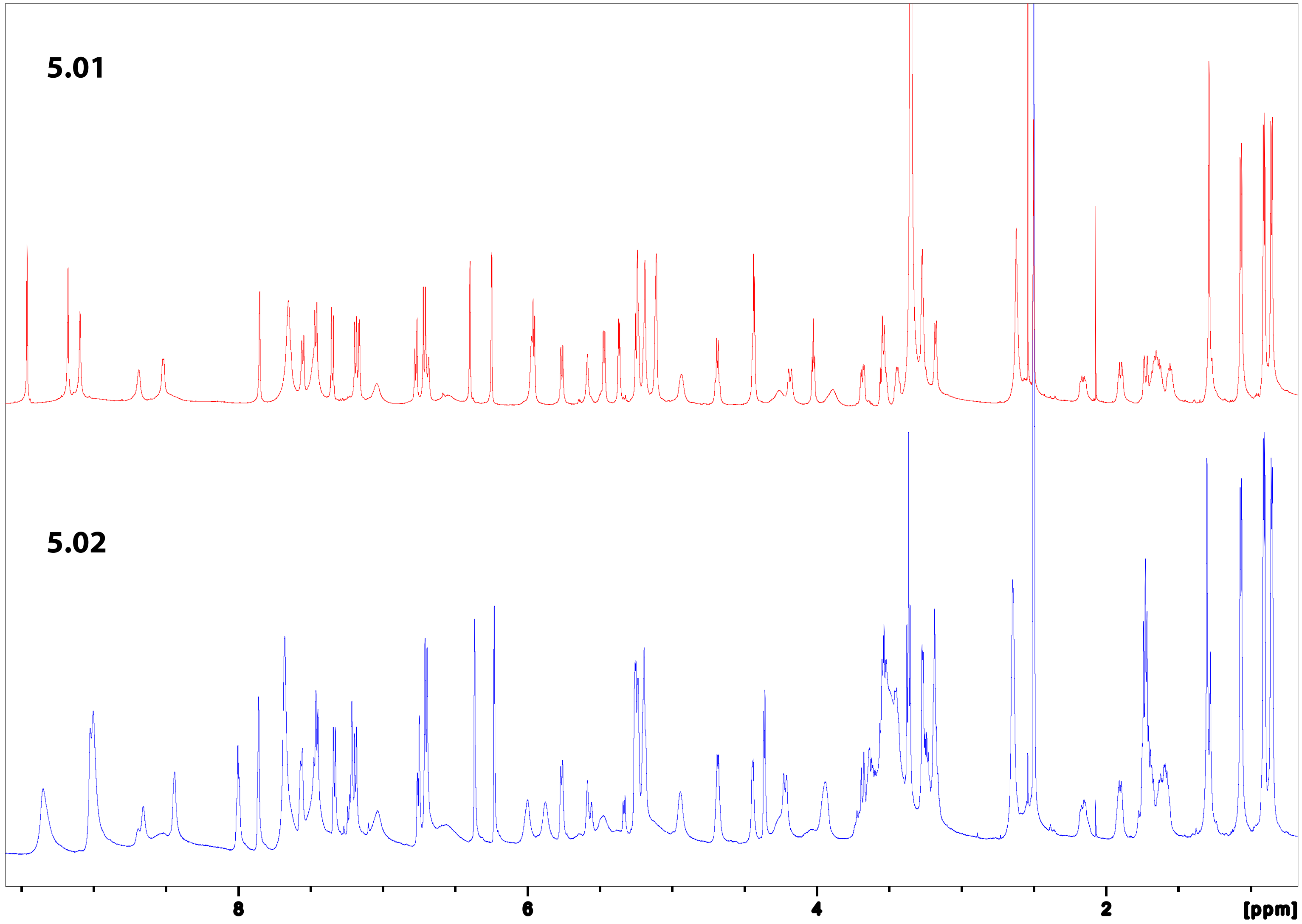

Figure S16. ^{1}H NMR (600 MHz, DMSO-d_6) of vanco-3C-N_3 **2** (blue) *vs* vancomycin **1** (red).

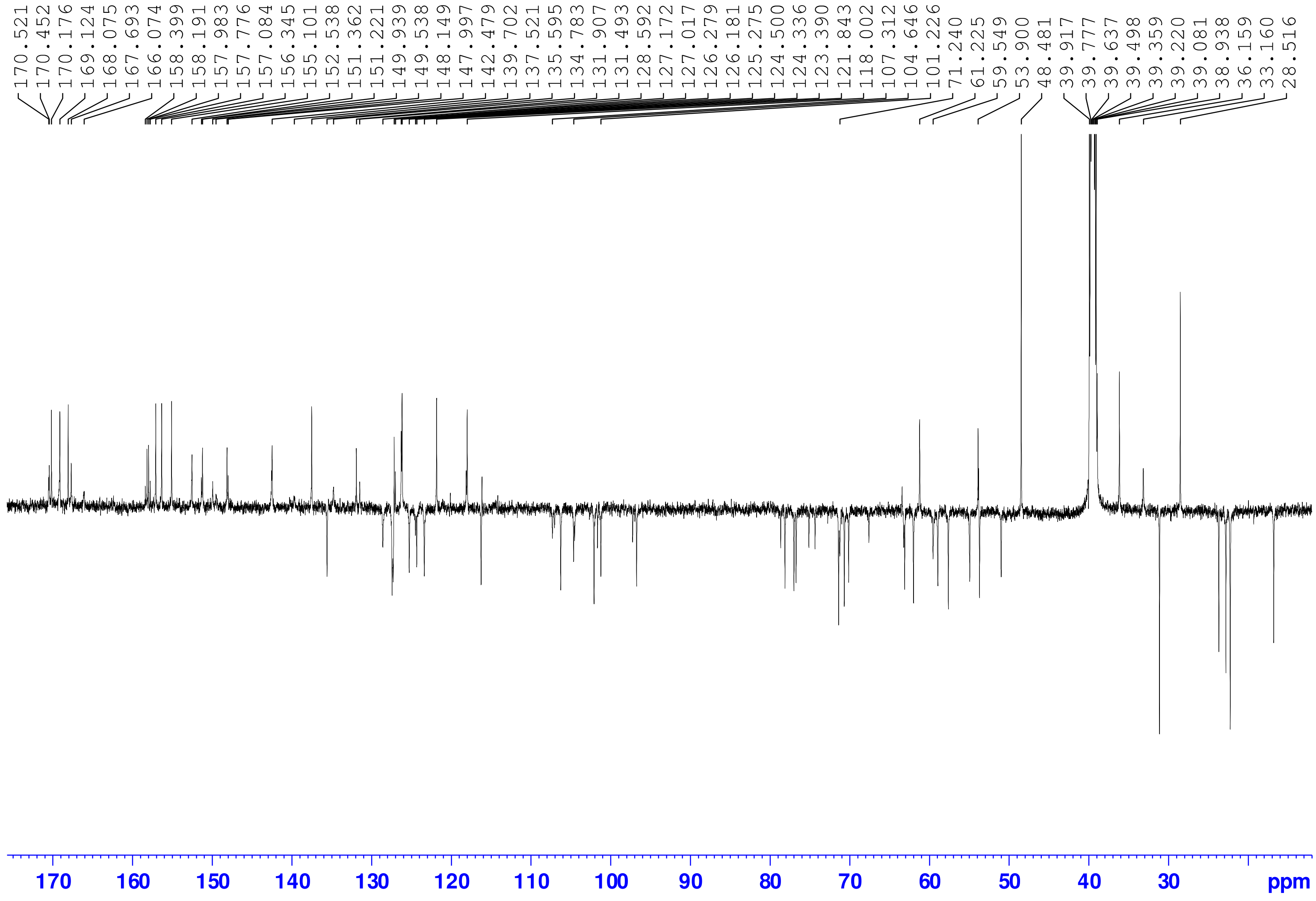

Figure S17. JMOD NMR (150 MHz, DMSO-d_6) of vanco-3C-N_3 **2**.

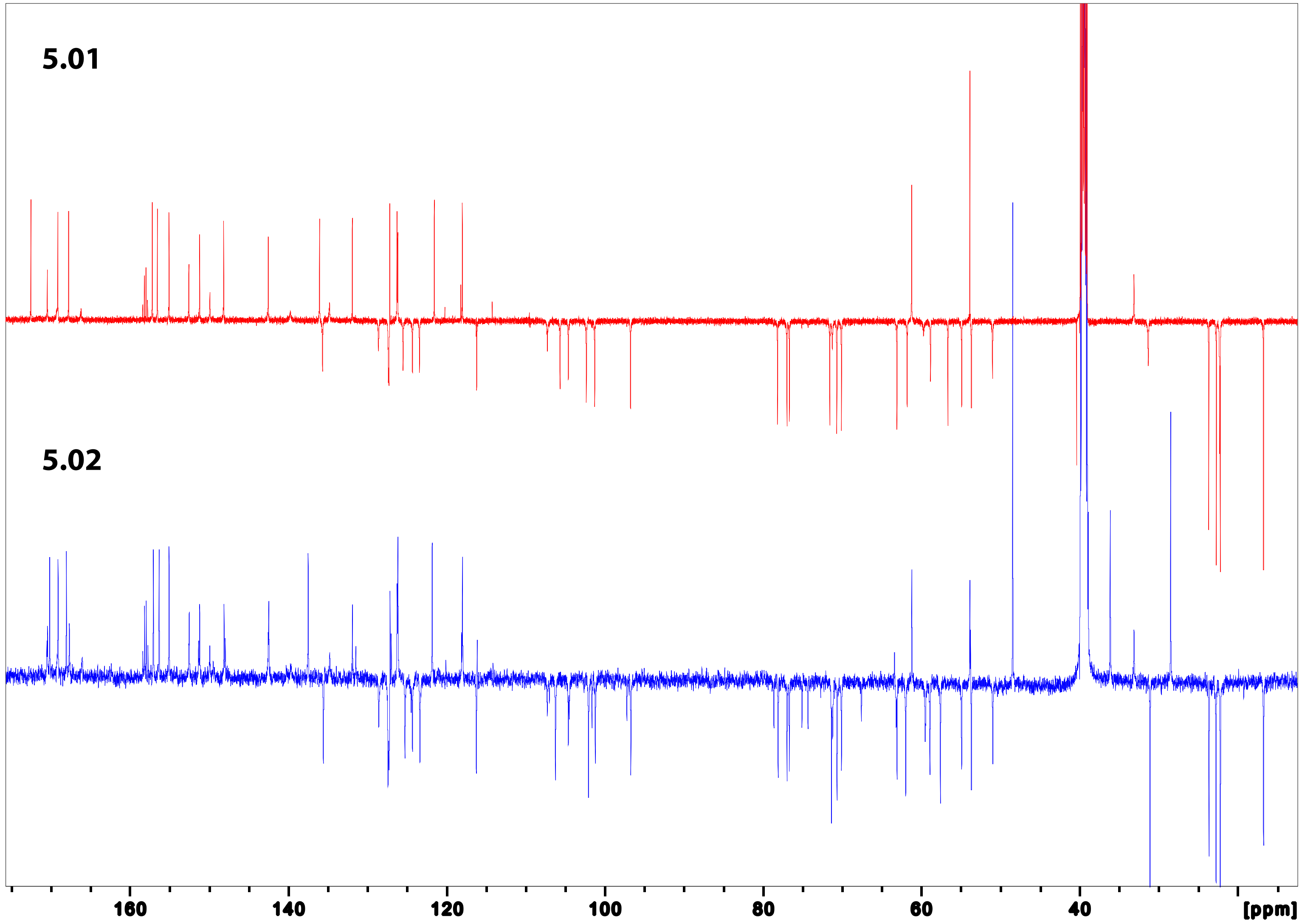

Figure S18. JMOD NMR (150 MHz, DMSO-d_6) of vanco-3C-N_3 **2**).

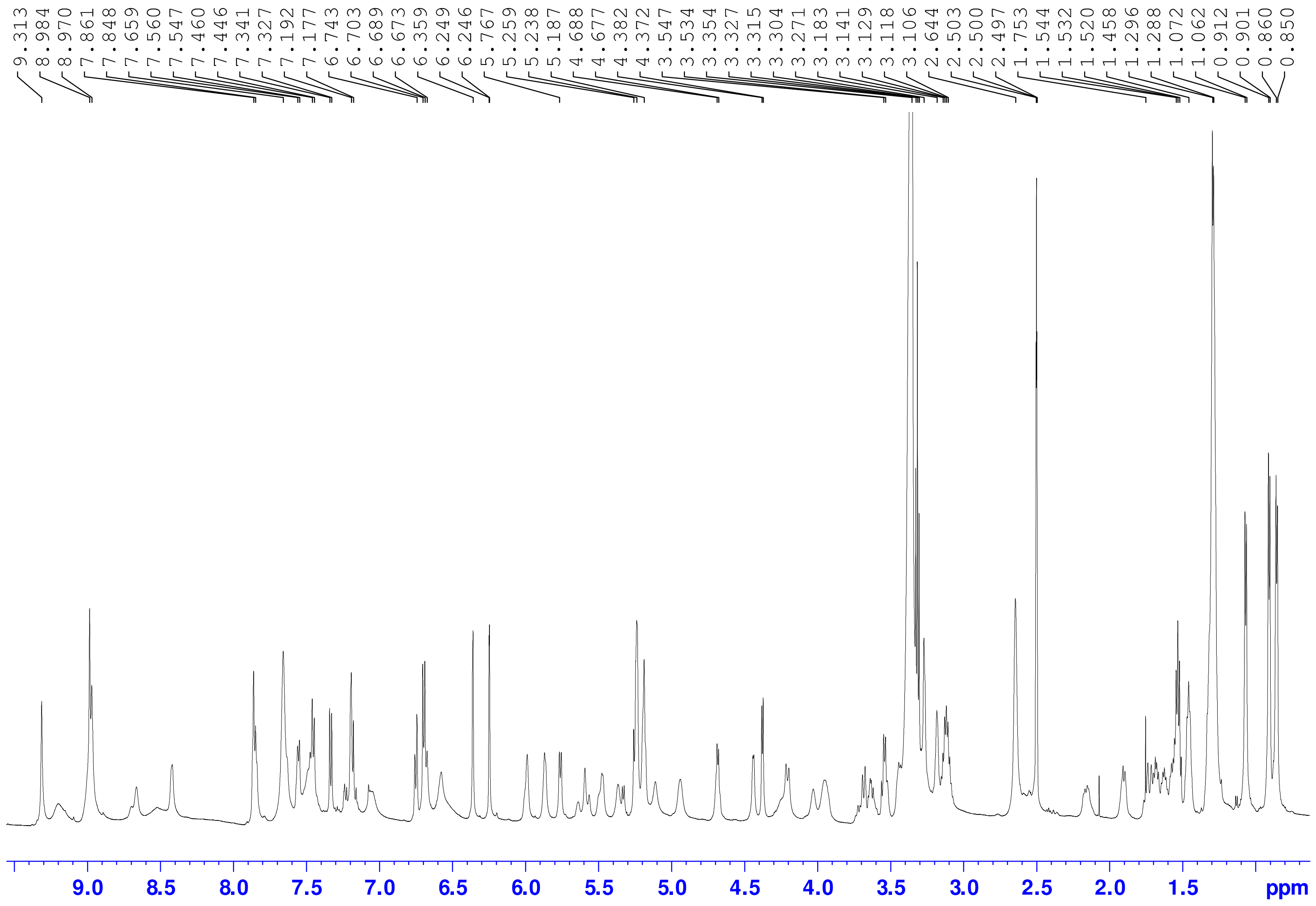

Figure S19. ^{1}H NMR (600 MHz, DMSO-d_6) of vanco-8C-N_3 **3**.

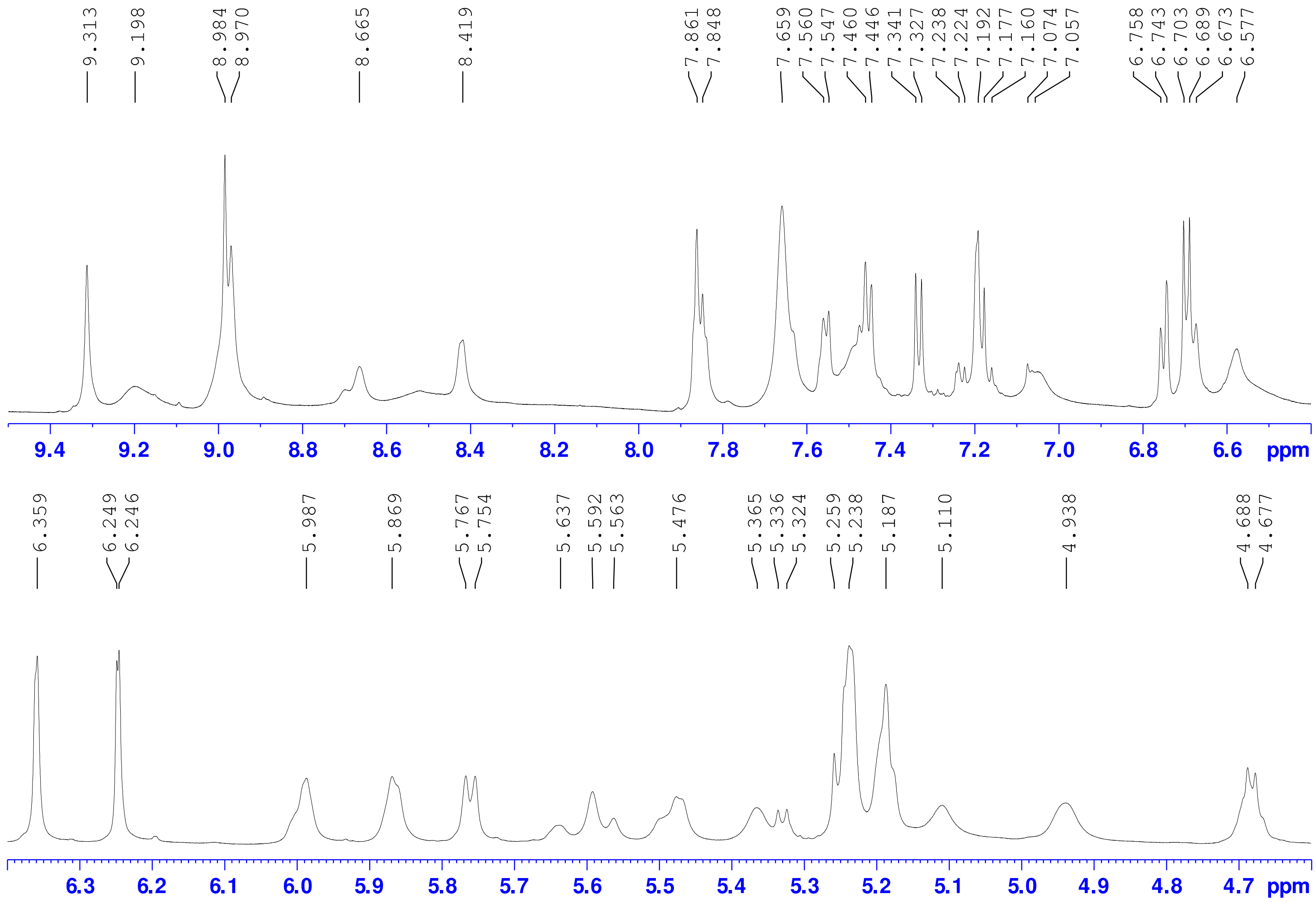

Figure S20. ^{1}H NMR (600 MHz, DMSO-d_6) of vanco-8C-N_3 **3**.

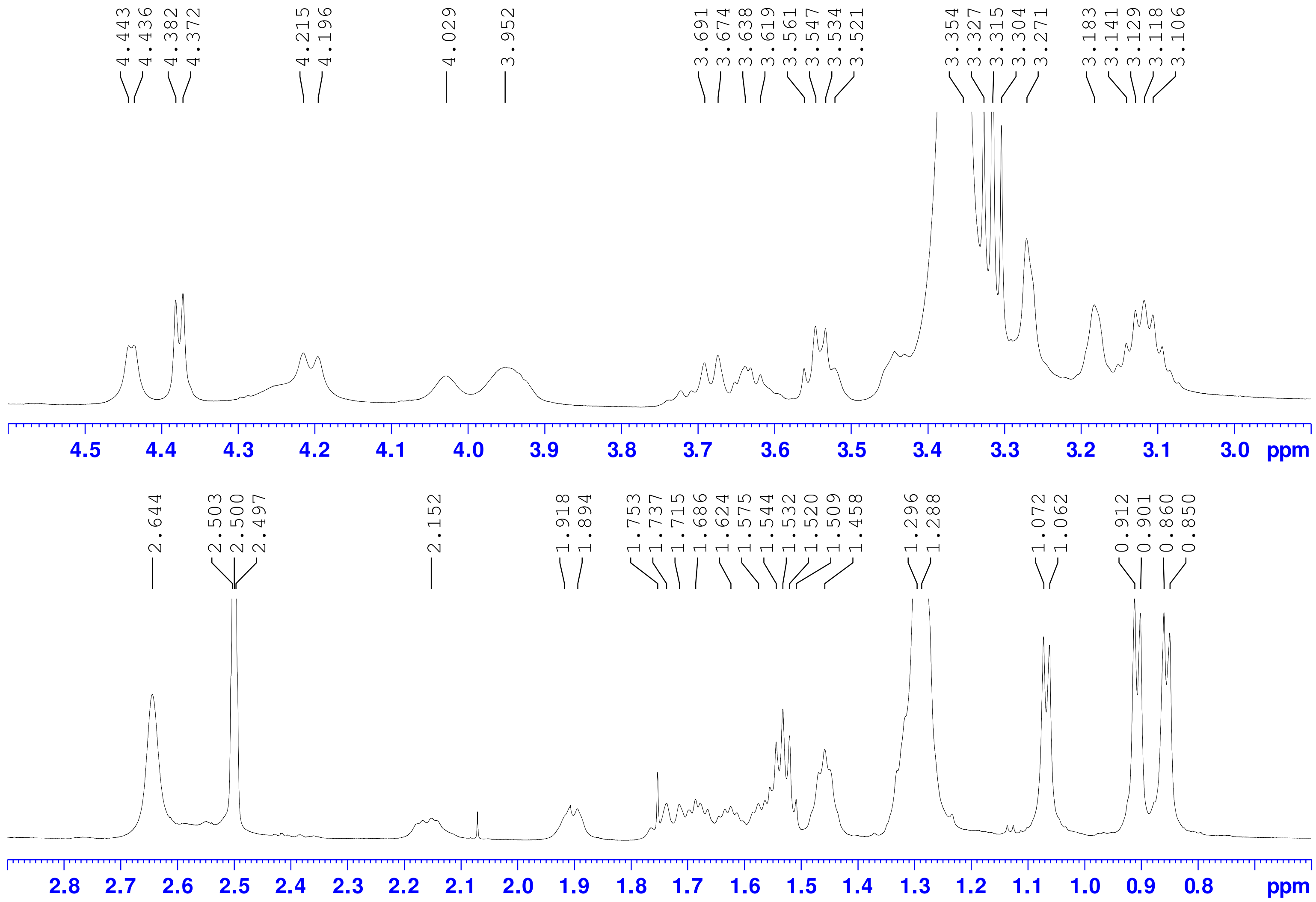

Figure S21. ^{1}H NMR (600 MHz, DMSO-d_6) of vanco-8C-N_3 **3**.

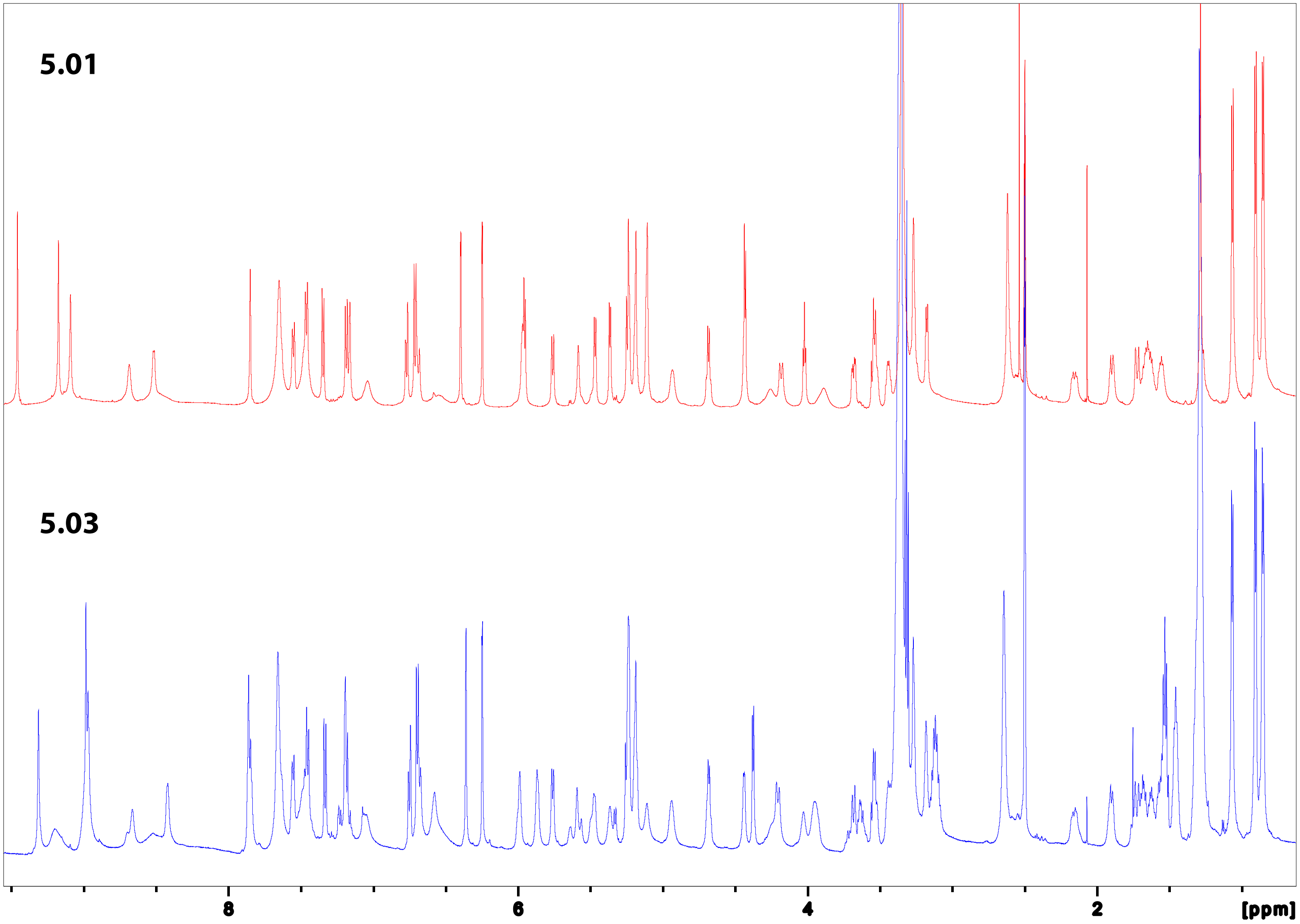

Figure S22. ^{1}H NMR (600 MHz, DMSO-d_6) of vanco-8C-N_3 **3** (blue) *vs* vancomycin **1** (red).

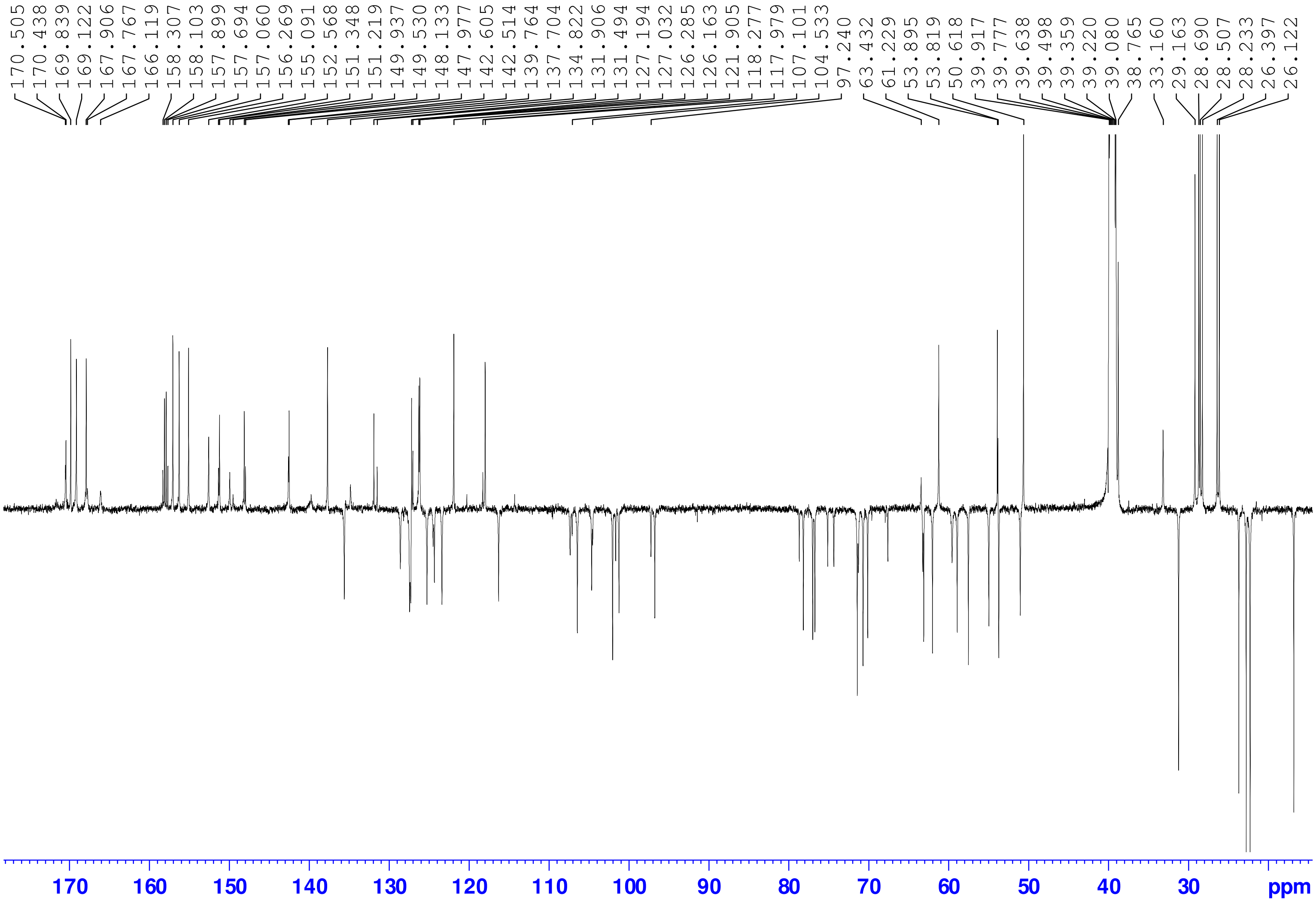

Figure S23. JMOD NMR (150 MHz, DMSO-d_6) of vanco-8C-N_3 **3**.

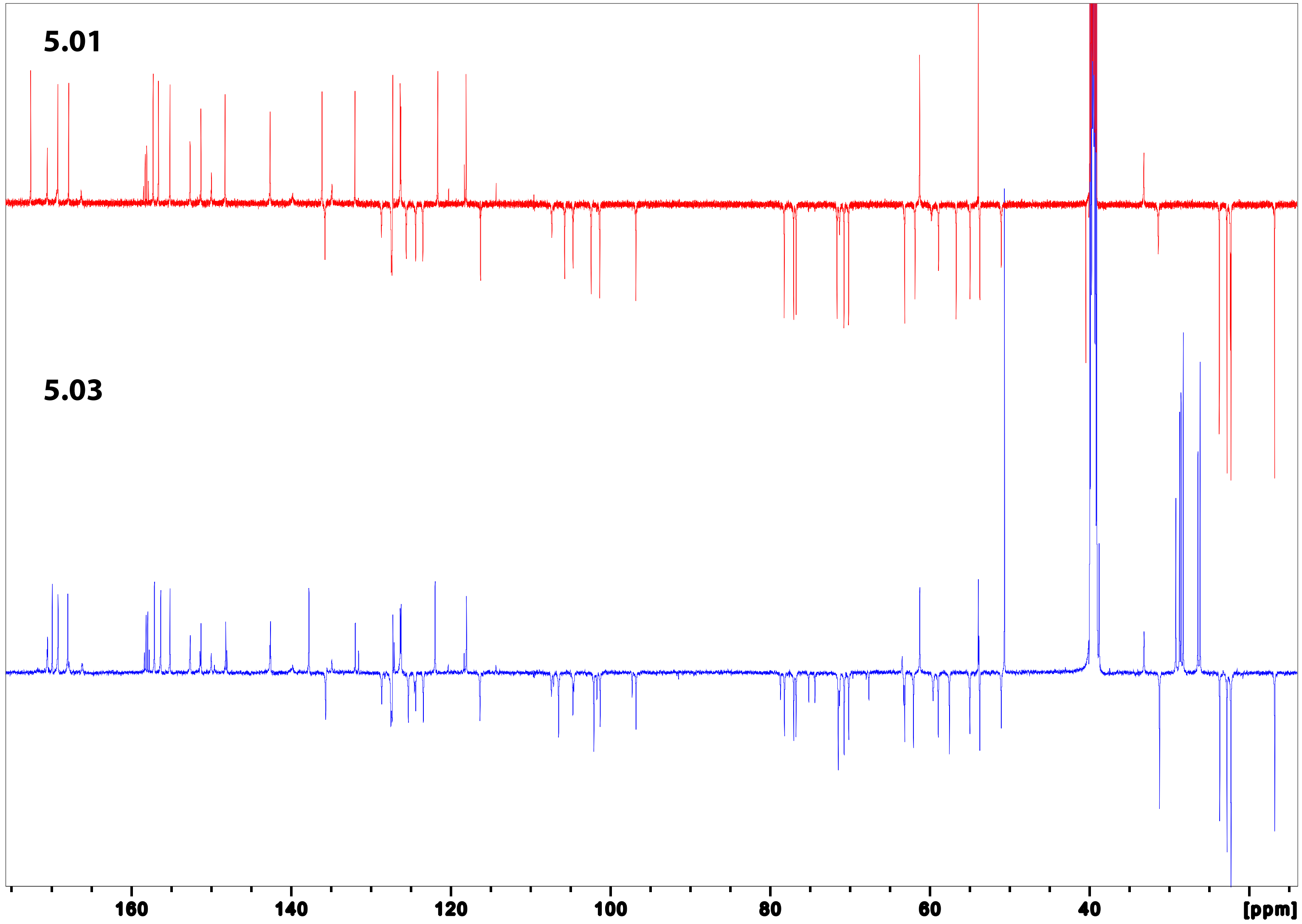

Figure S24. JMOD NMR (150 MHz, DMSO-d_6) of vanco-8C-N_3 **3** (blue) *vs* vancomycin **1** (red).

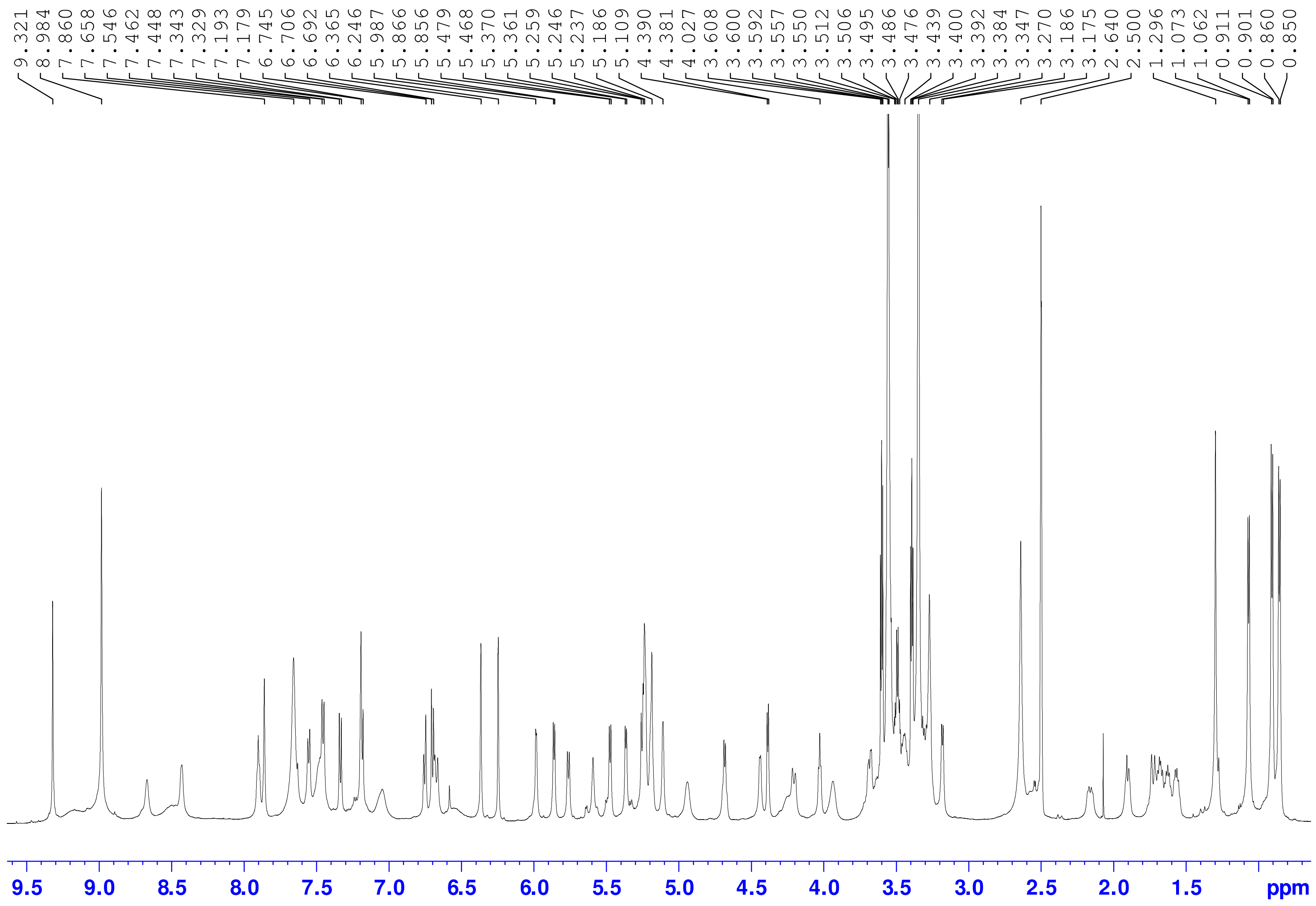

Figure S25. ^{1}H NMR (600 MHz, DMSO-d_6) of vanco-3PEG-N_3 **4**.

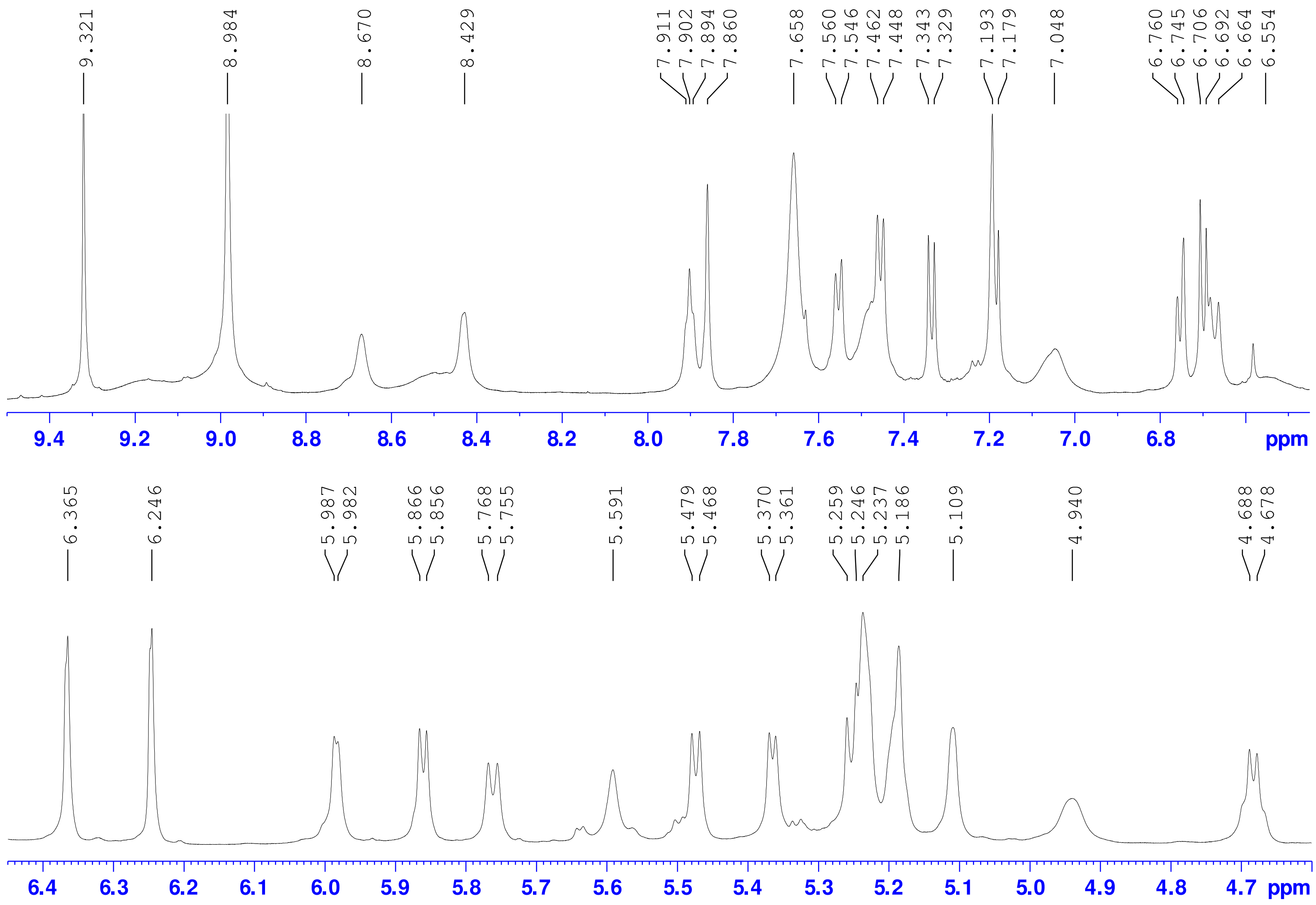

Figure S26. ^{1}H NMR (600 MHz, DMSO-d_6) of vanco-3PEG-N_3 **4**.

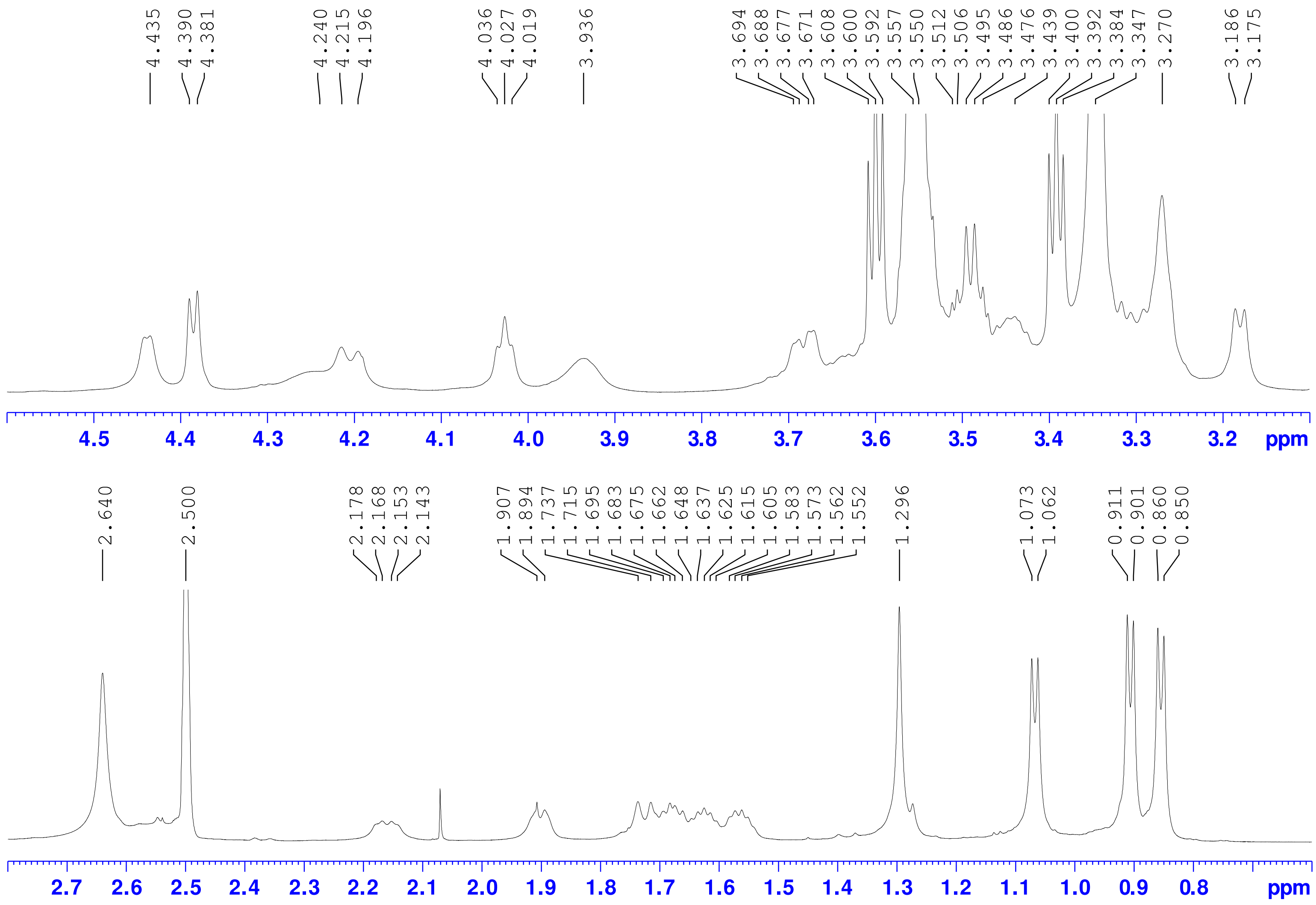

Figure S27. ^{1}H NMR (600 MHz, DMSO-d_6) of vanco-3PEG-N_3 **4**.

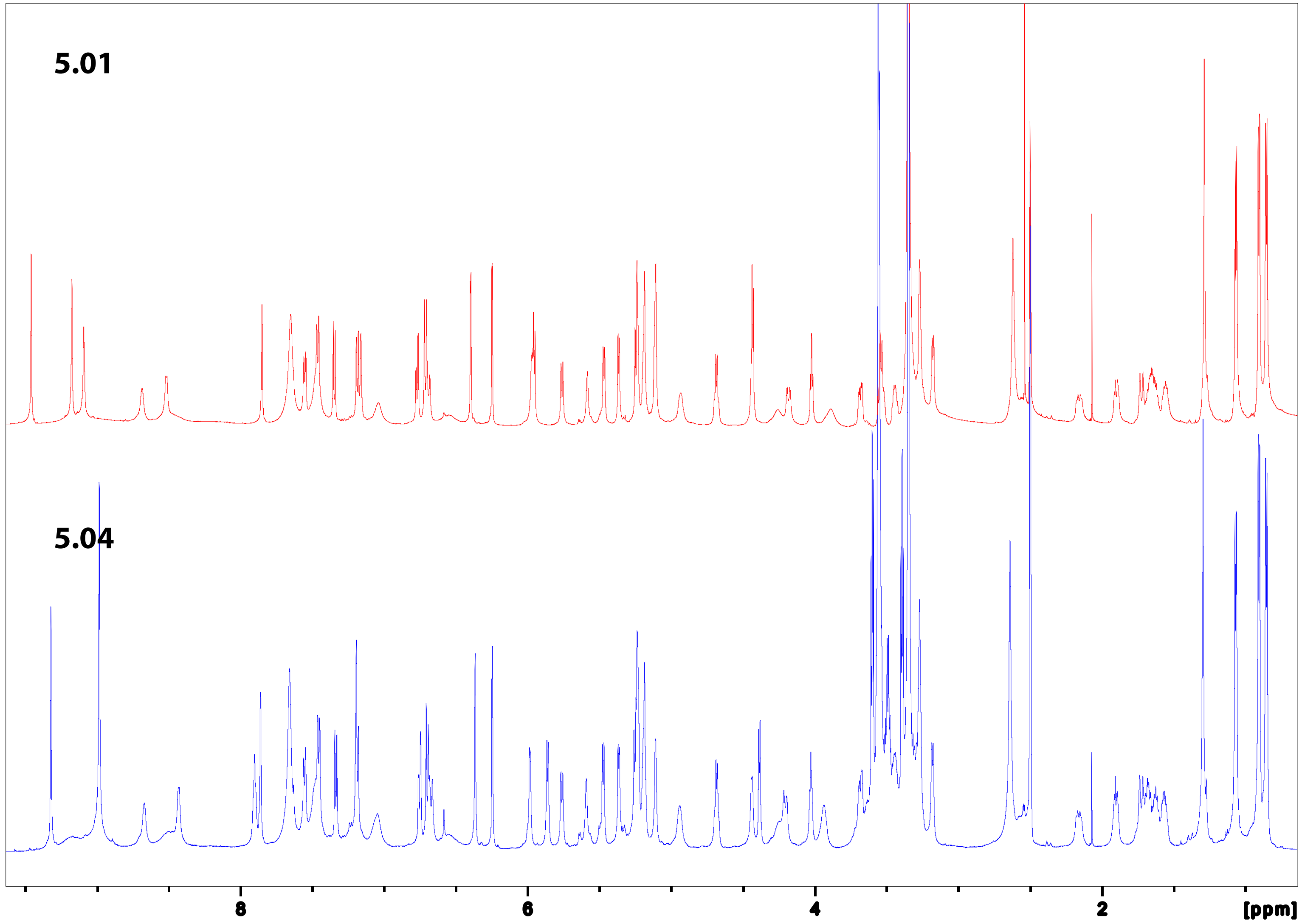

Figure S28. ^{1}H NMR (600 MHz, DMSO-d_6) of vanco-3PEG-N_3 **4** (blue) *vs* vancomycin **1** (red).

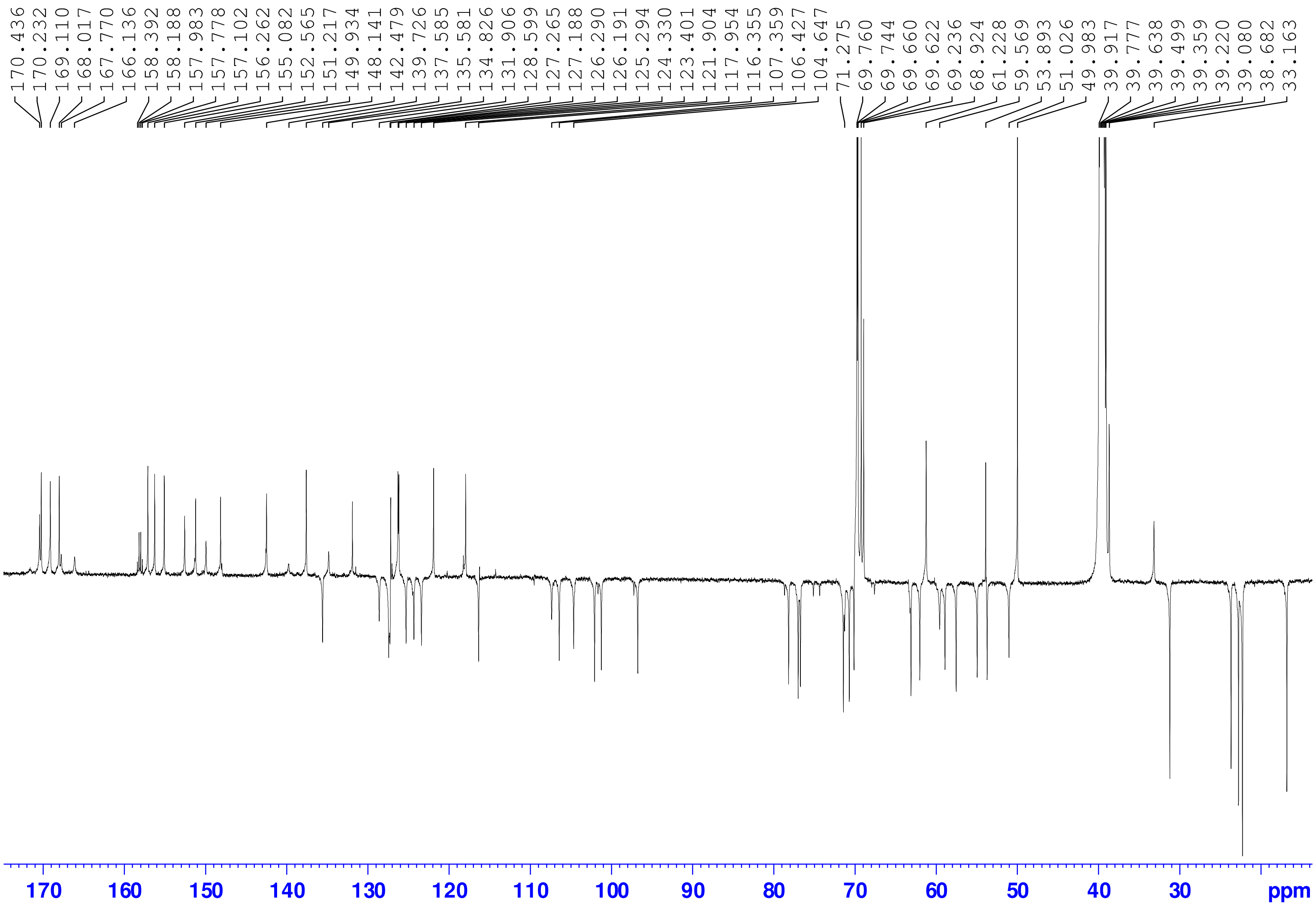

Figure S29. JMOD NMR (150 MHz, DMSO-d_6) of vanco-3PEG-N_3 **4**.

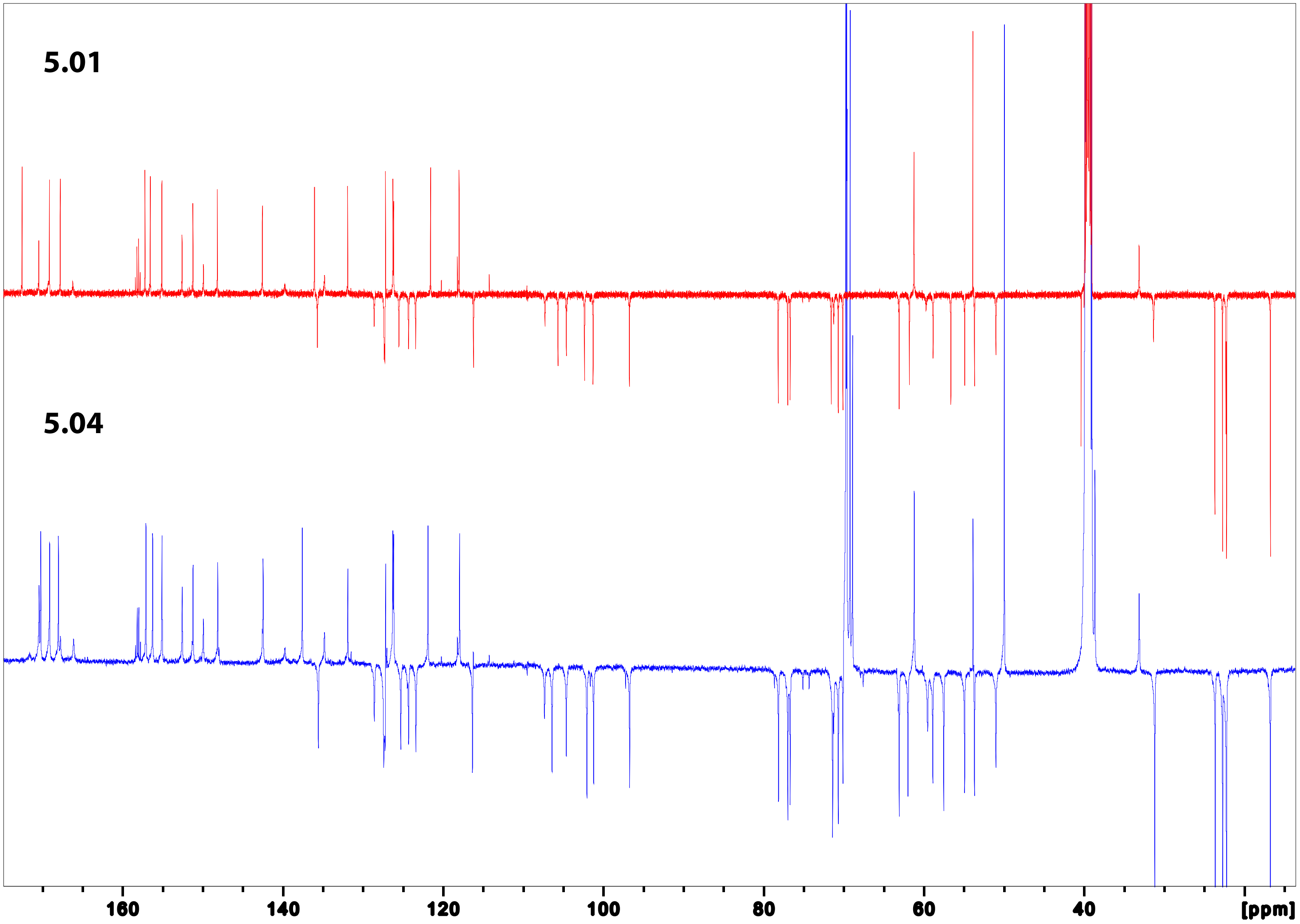

Figure S30. JMOD NMR (150 MHz, DMSO-d_6) of vanco-3PEG-N_3 (blue) *vs* vancomycin **1** (red).

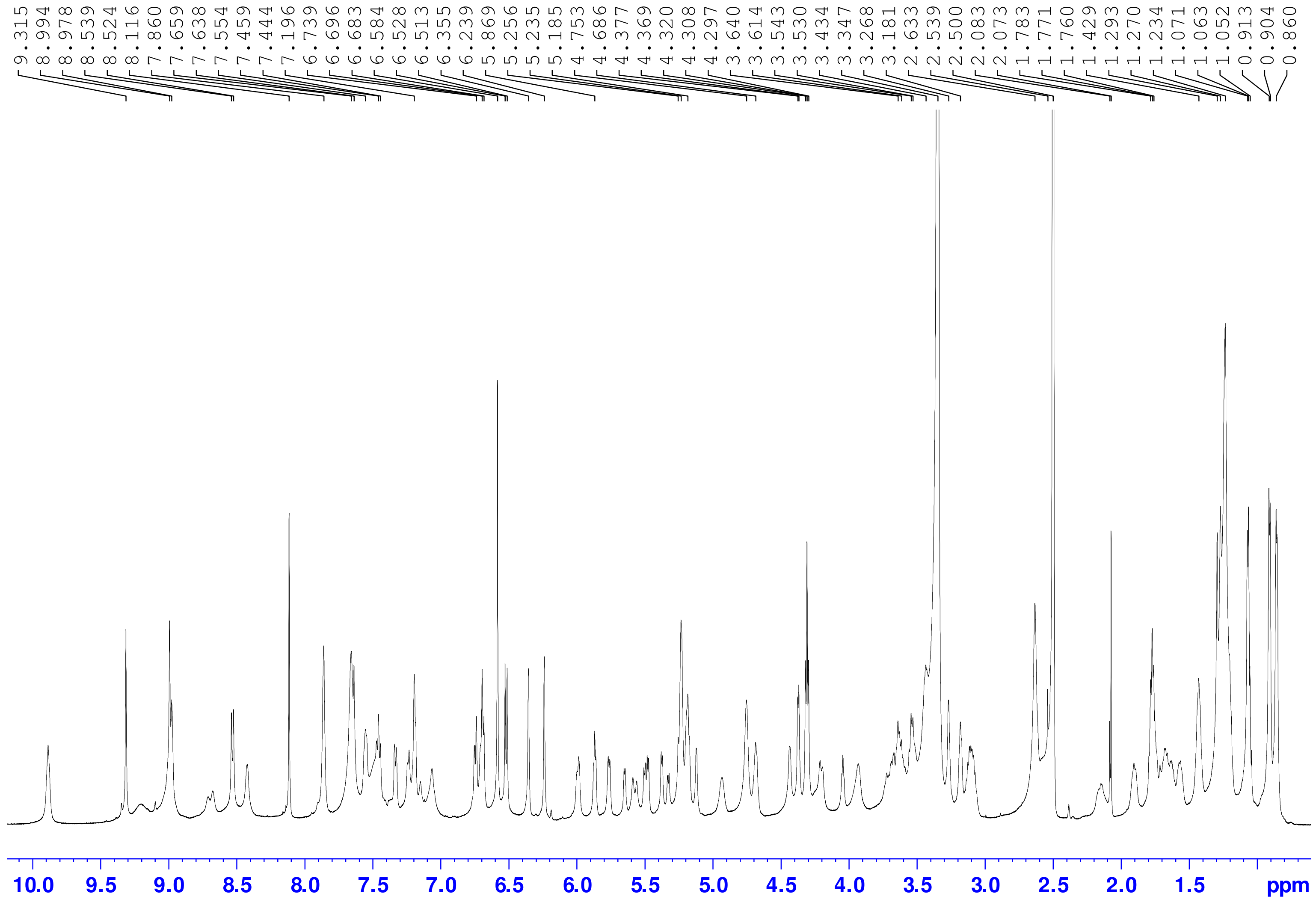

Figure S31. ^{1}H NMR (600 MHz, DMSO-d_6) of vanco-8C-Tz-NBD **7**.

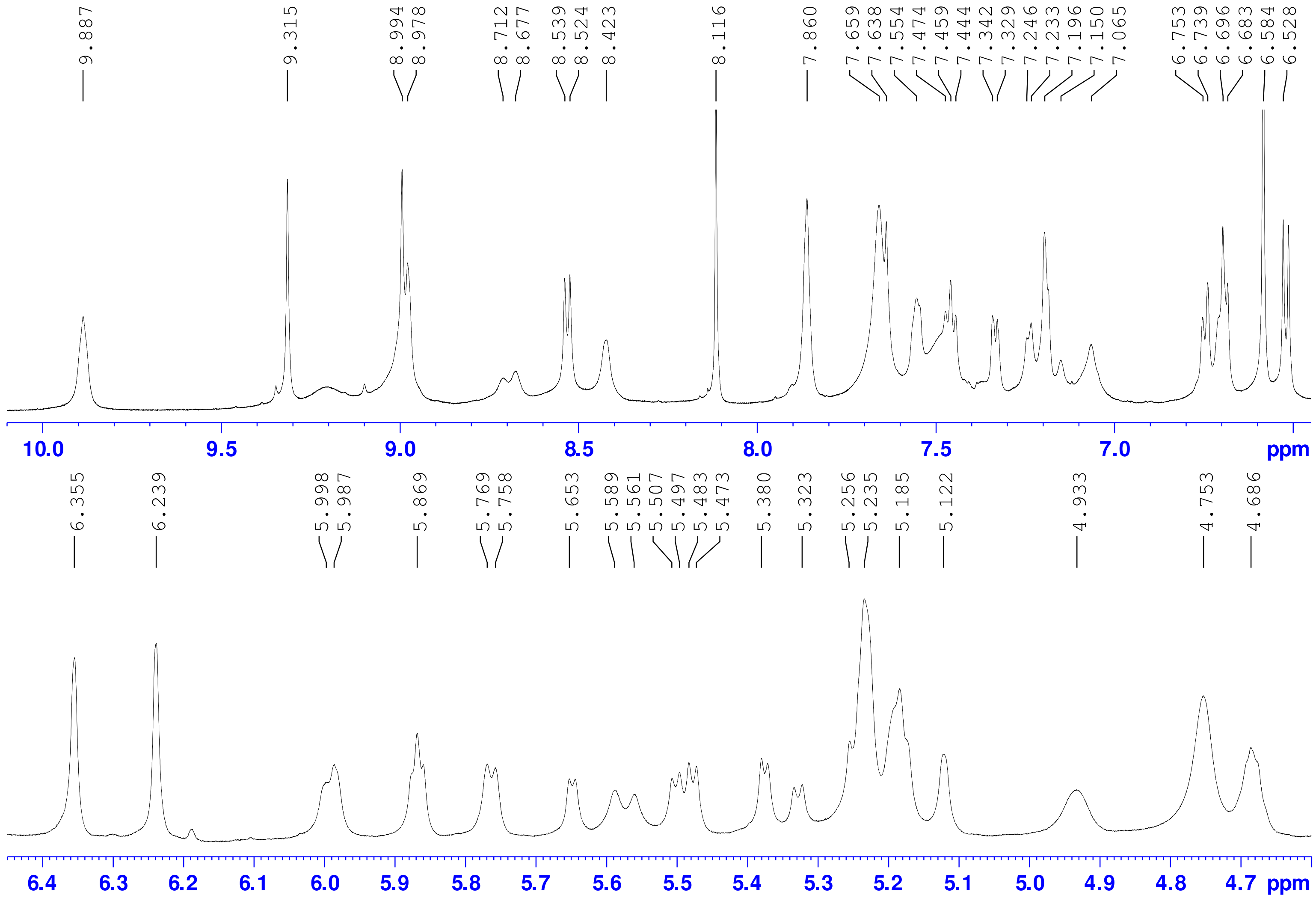

Figure S32. ^{1}H NMR (600 MHz, DMSO-d_6) of vanco-8C-Tz-NBD **7**.

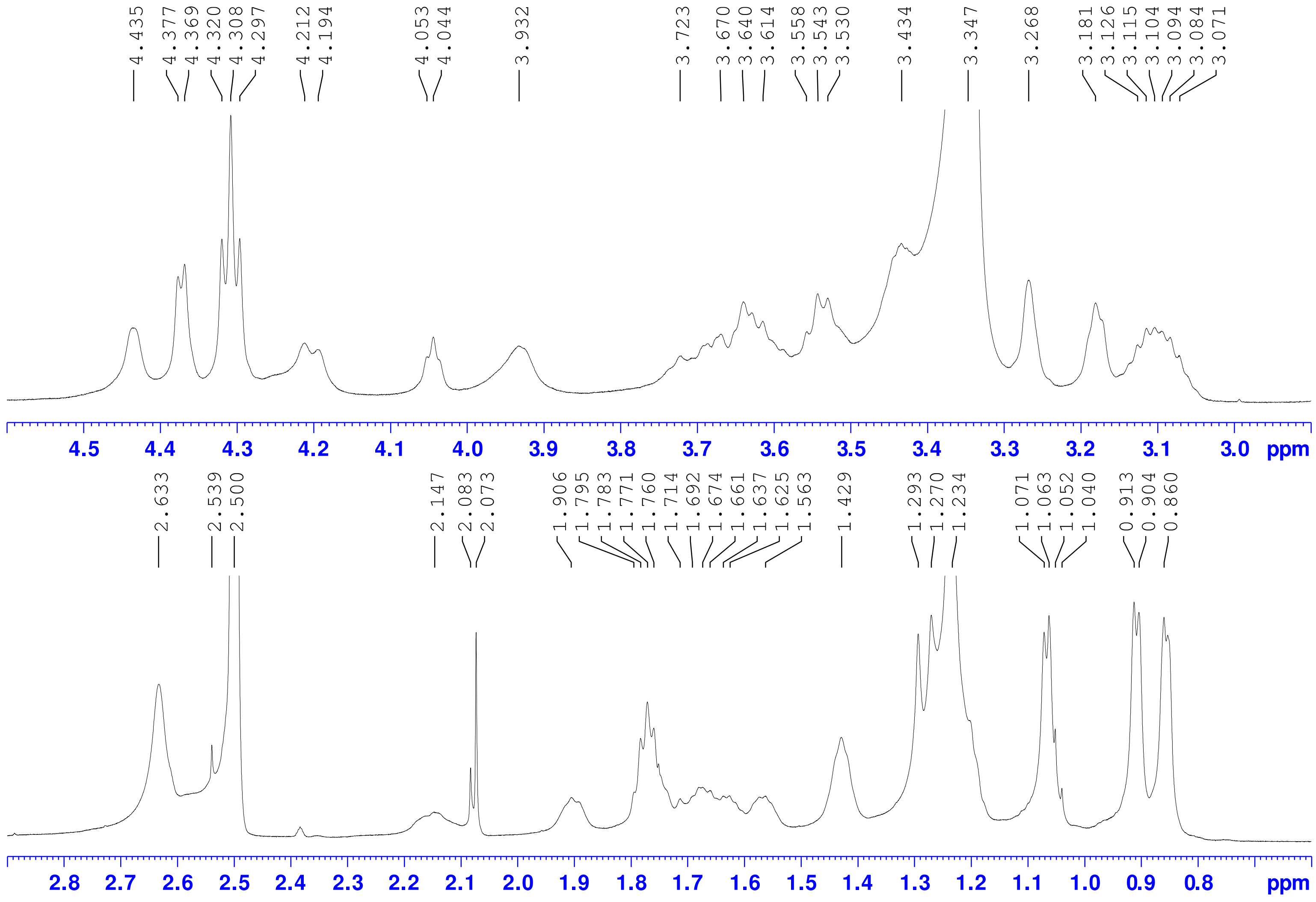

Figure S33. ^{1}H NMR (600 MHz, DMSO-d_6) of vanco-8C-Tz-NBD **7**.

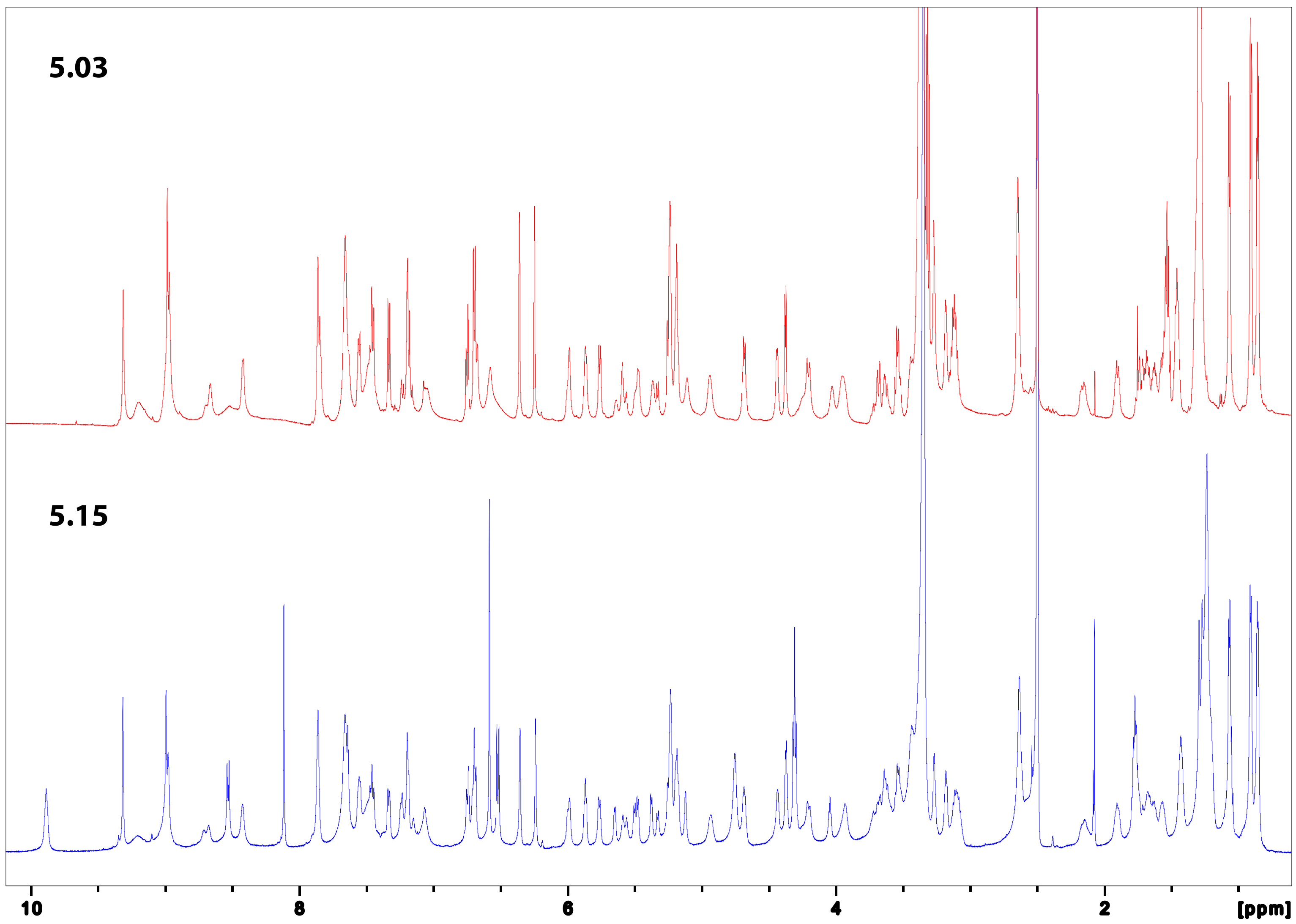

Figure S34. ^{1}H NMR (600 MHz, DMSO-d_6) of vanco-8C-Tz-NBD **7** (blue) *vs* vanco-8C-N_3 **3** (red).

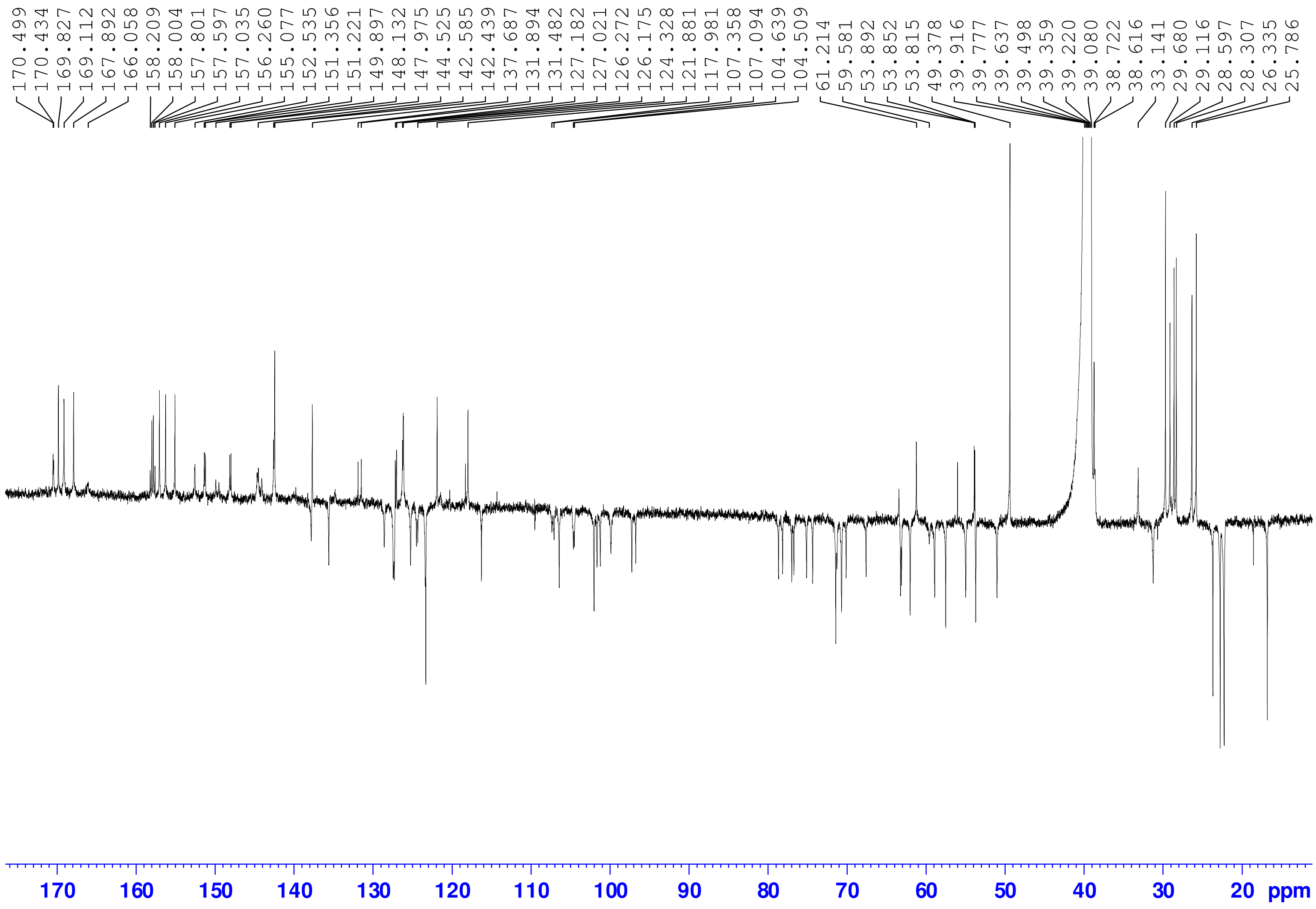

Figure S35. JMOD NMR (150 MHz, DMSO-d_6) of vanco-8C-Tz-NBD **7**.

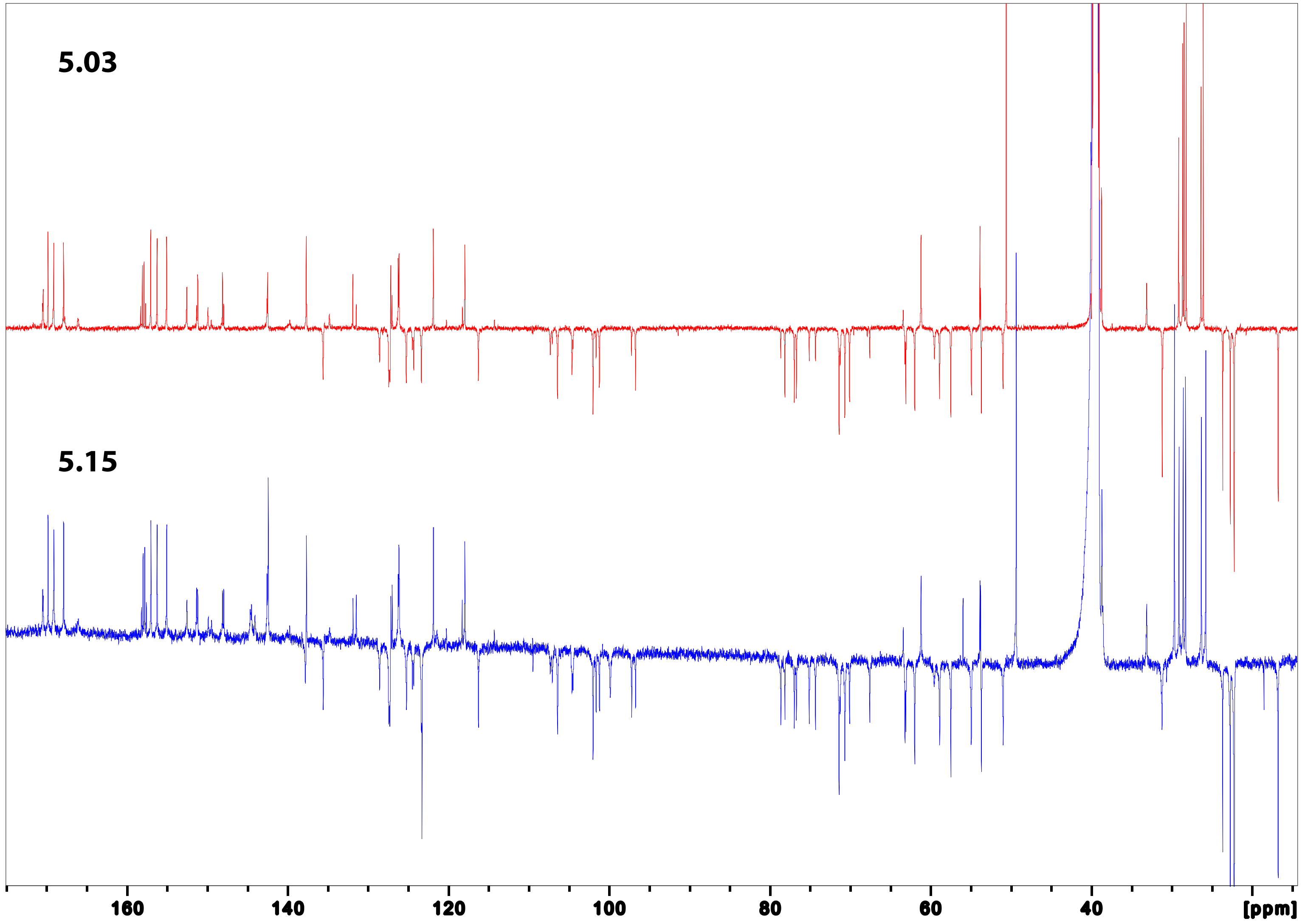

Figure S36. JMOD NMR (150 MHz, DMSO-d_6) of vanco-8C-Tz-NBD **7** (blue) *vs* vanco-8C-N_3 **3** (red).

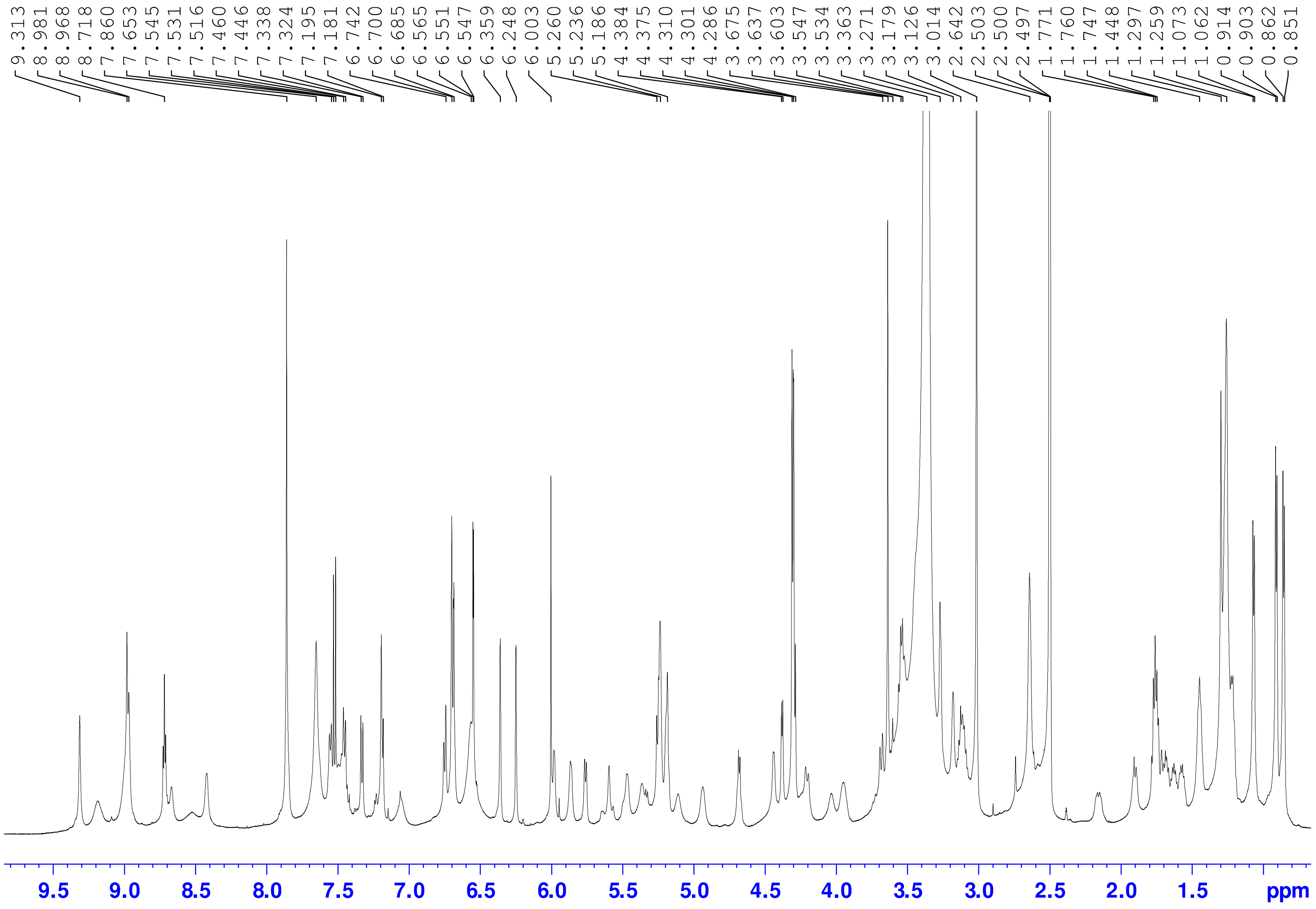

Figure S37. ^{1}H NMR (600 MHz, DMSO-d_6) of vanco-8C-Tz-DMACA **8**.

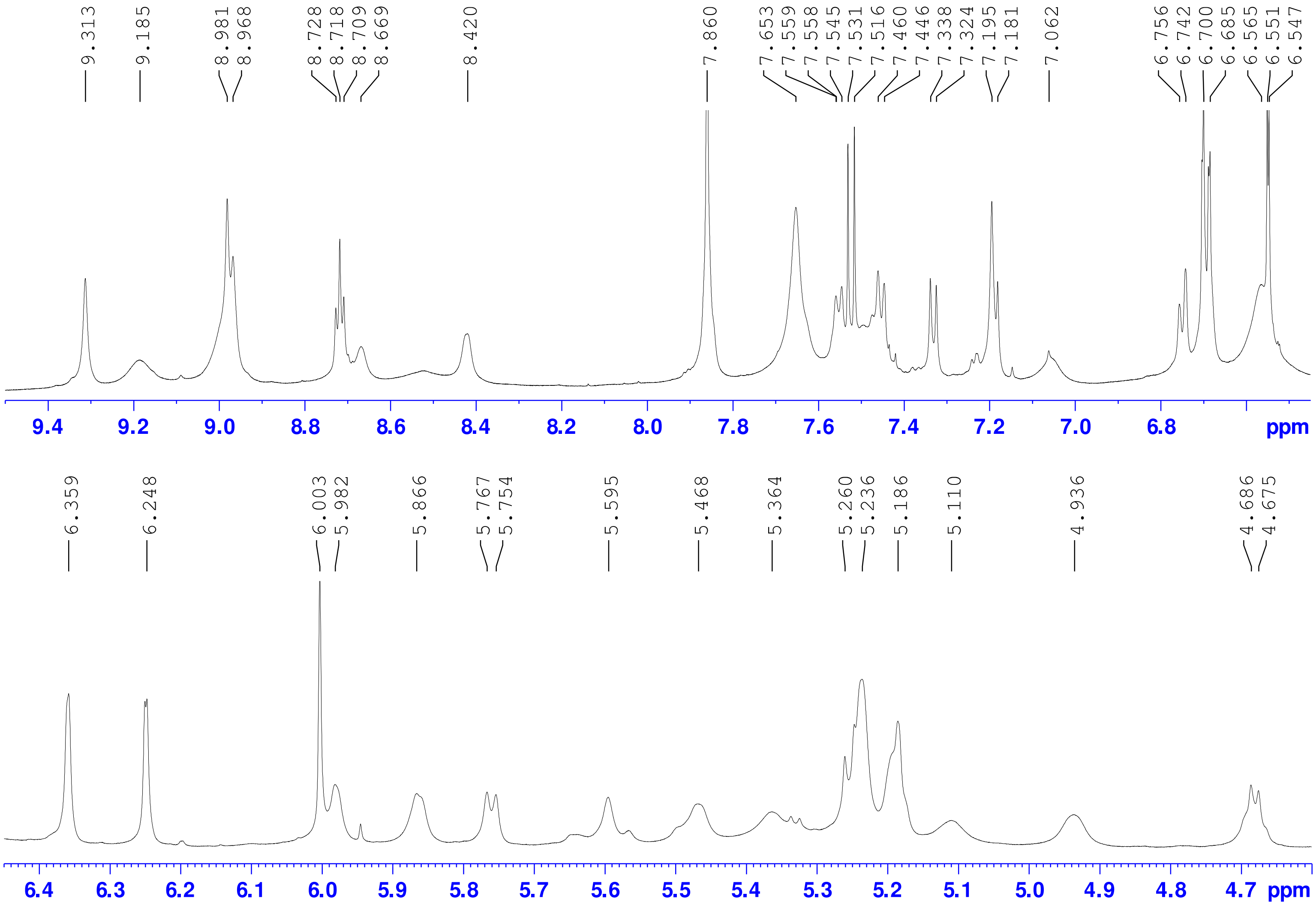

Figure S38. ^{1}H NMR (600 MHz, DMSO-d_6) of vanco-8C-Tz-DMACA **8**.

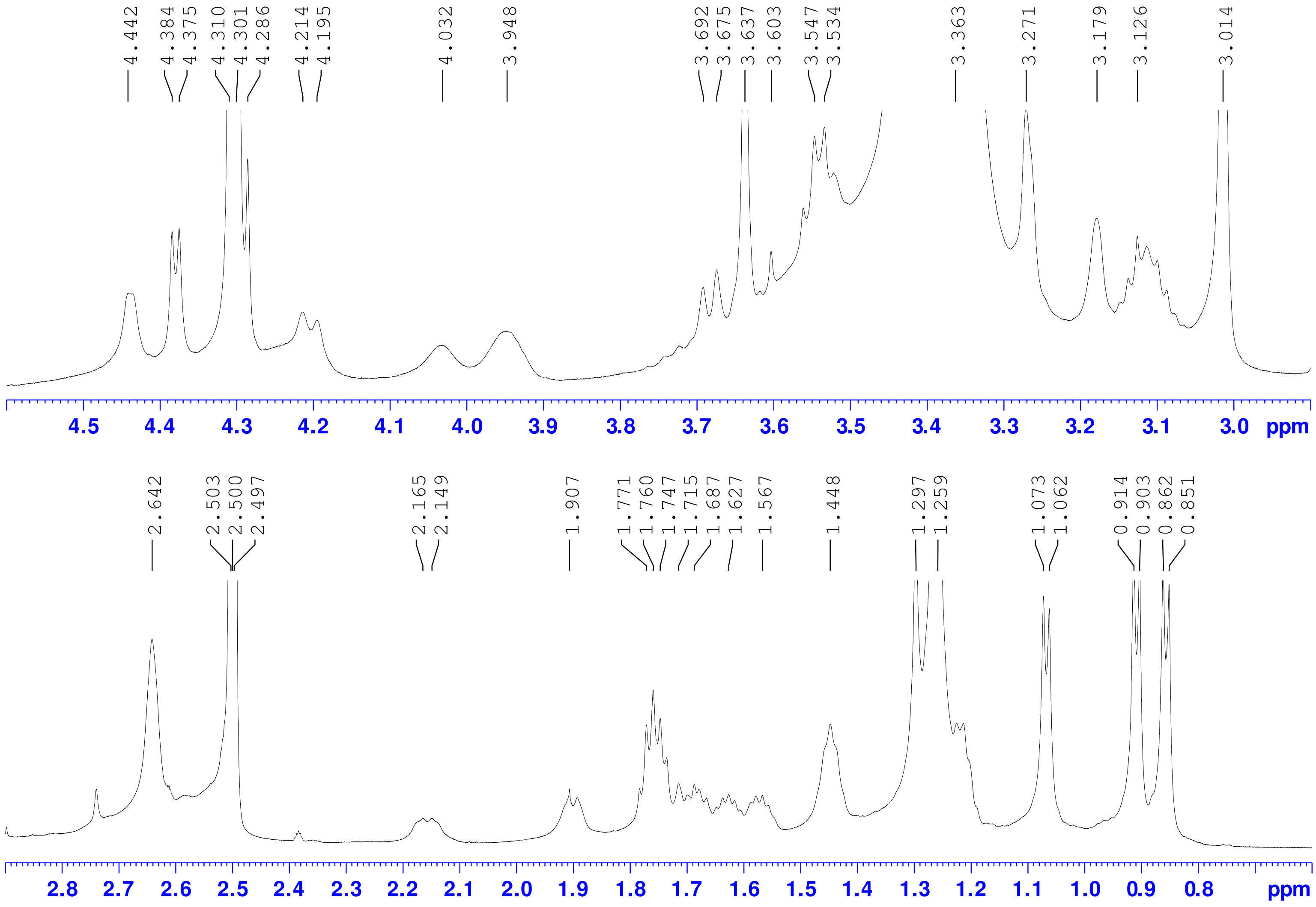

Figure S39. ^{1}H NMR (600 MHz, DMSO-d_6) of vanco-8C-Tz-DMACA **8**.

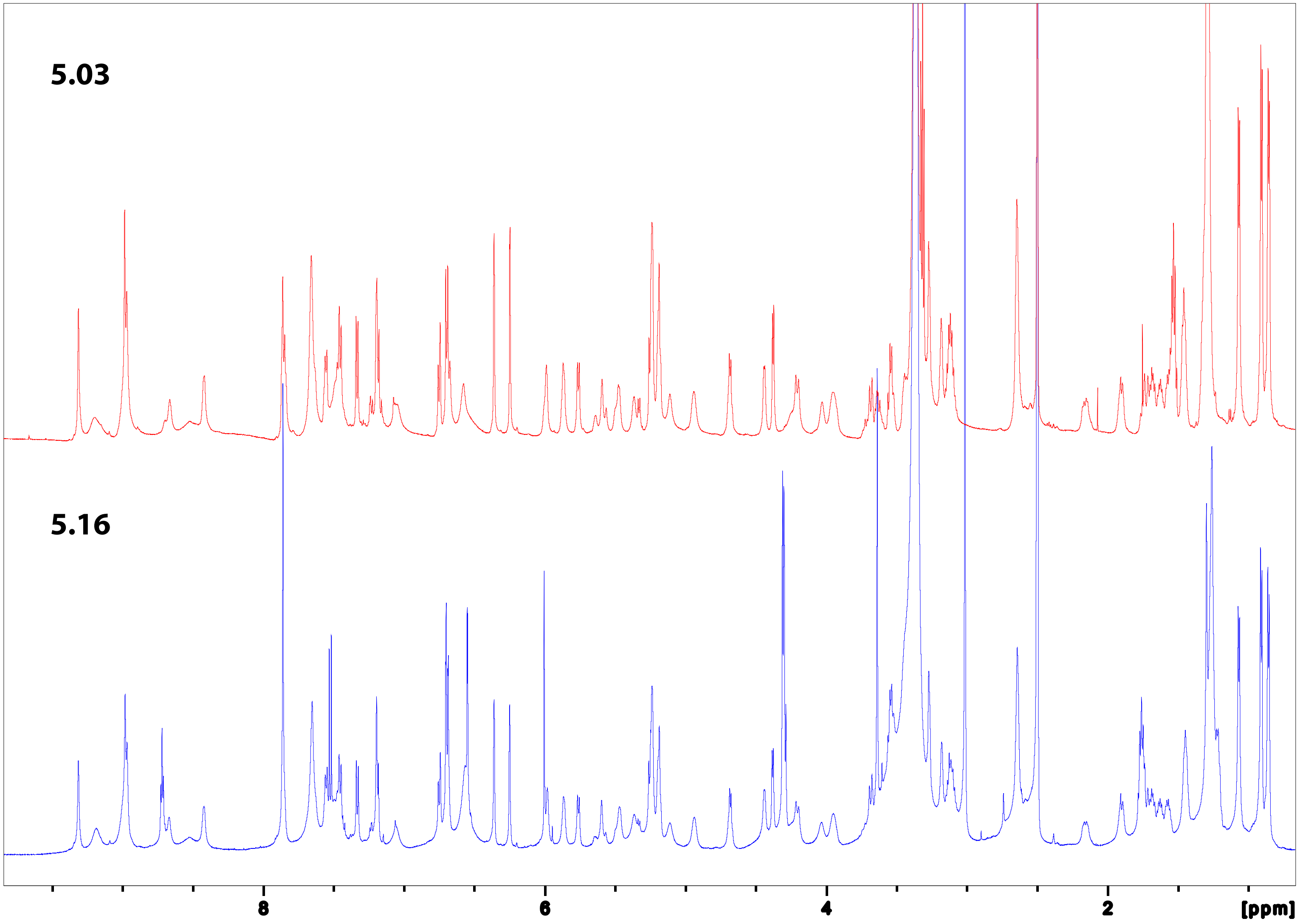

Figure S40. ^{1}H NMR (600 MHz, DMSO-d_6) of vanco-8C-Tz-DMACA **8** (blue) *vs* vanco-8C-N_3 **3** (red).

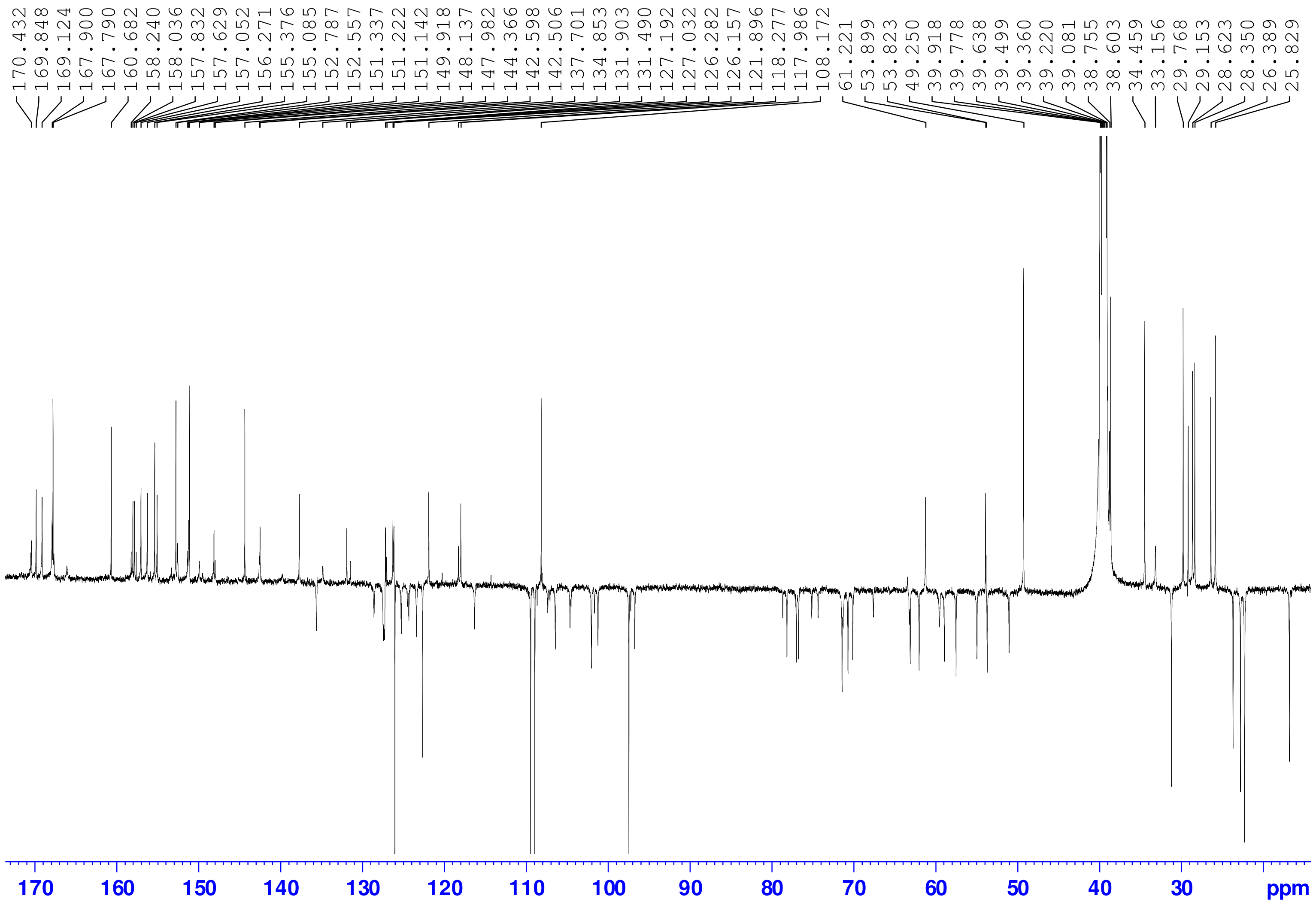

Figure S41. JMOD NMR (150 MHz, DMSO-d_6) of vanco-8C-Tz-DMACA **8**.

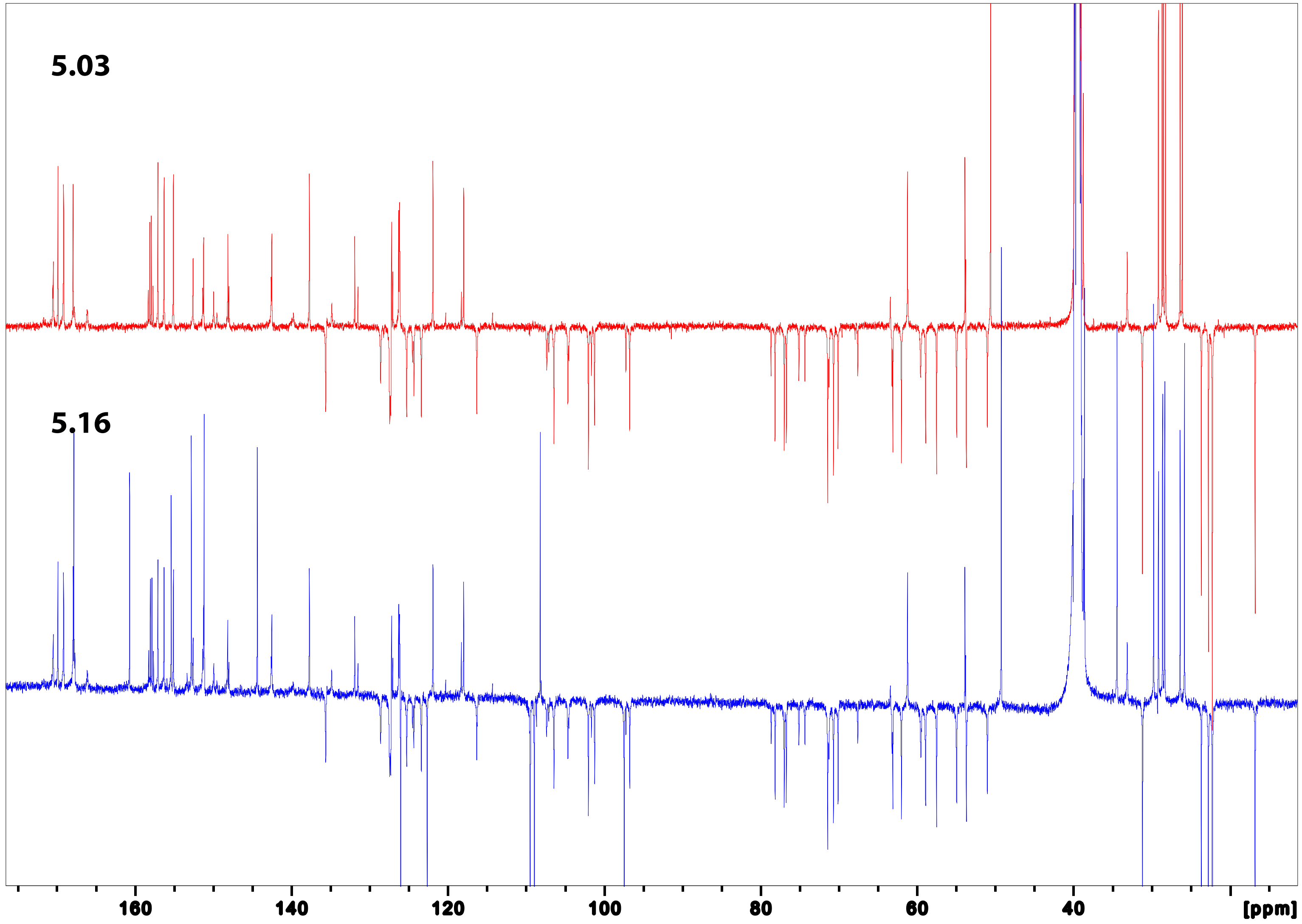

Figure S42. JMOD NMR (150 MHz, DMSO-d_6) of vanco-8C-Tz-DMACA **8** (blue) *vs* vanco-8C-N_3 **3** (red).

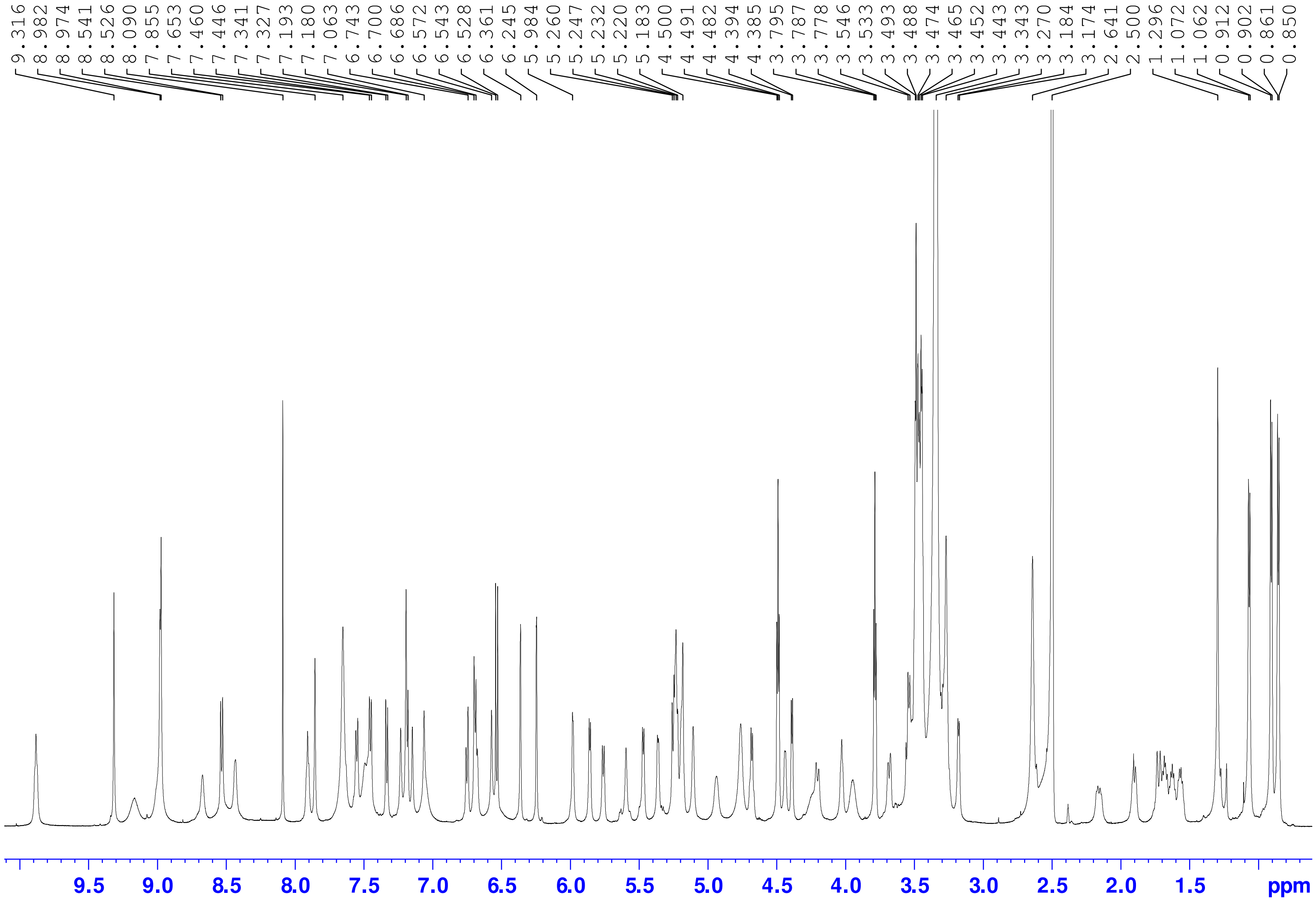

Figure S43. ^{1}H NMR (600 MHz, DMSO-d_6) of vanco-3PEG-Tz-NBD **9**.

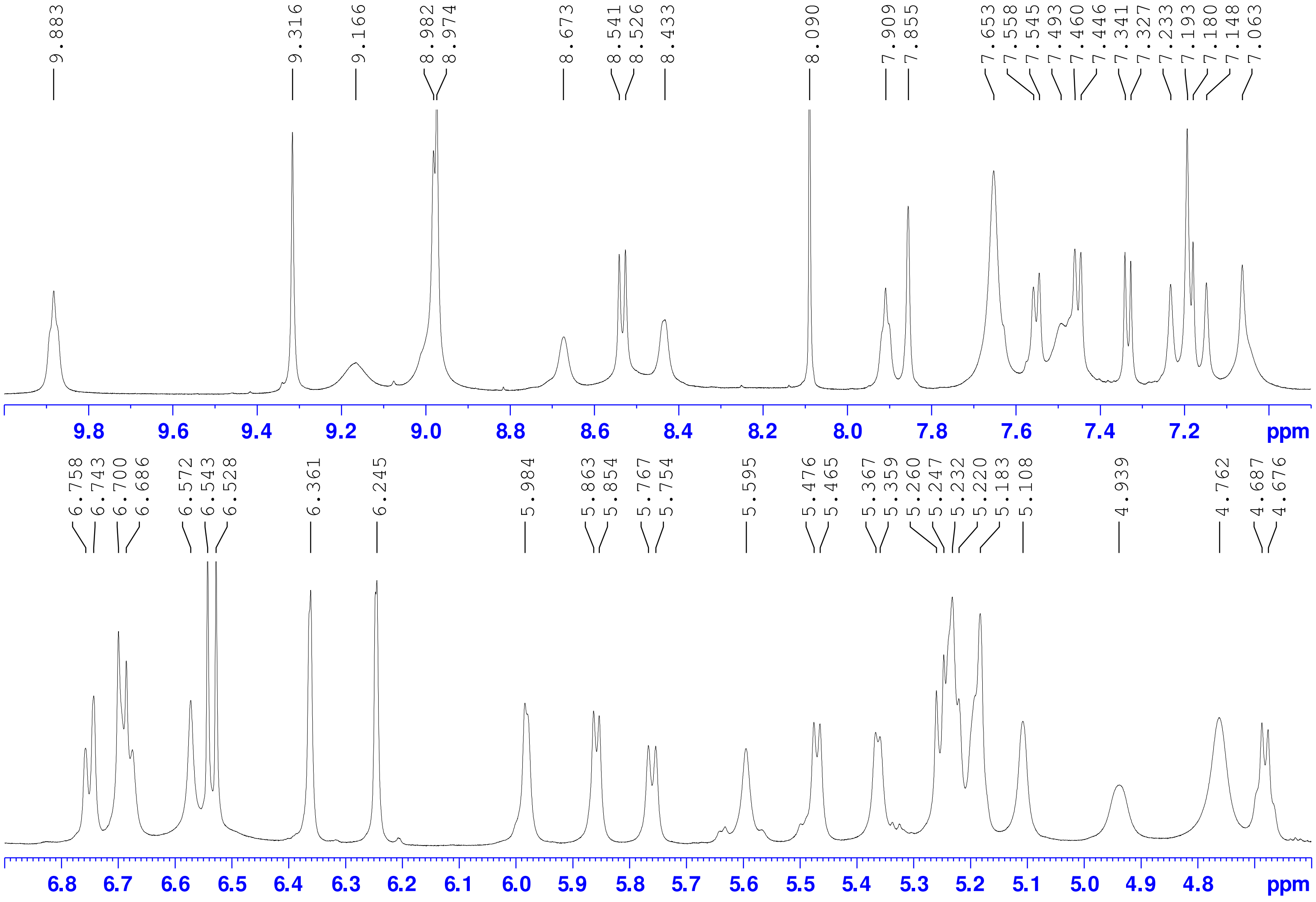

Figure S44. ^{1}H NMR (600 MHz, DMSO-d_6) of vanco-3PEG-Tz-NBD **9**.

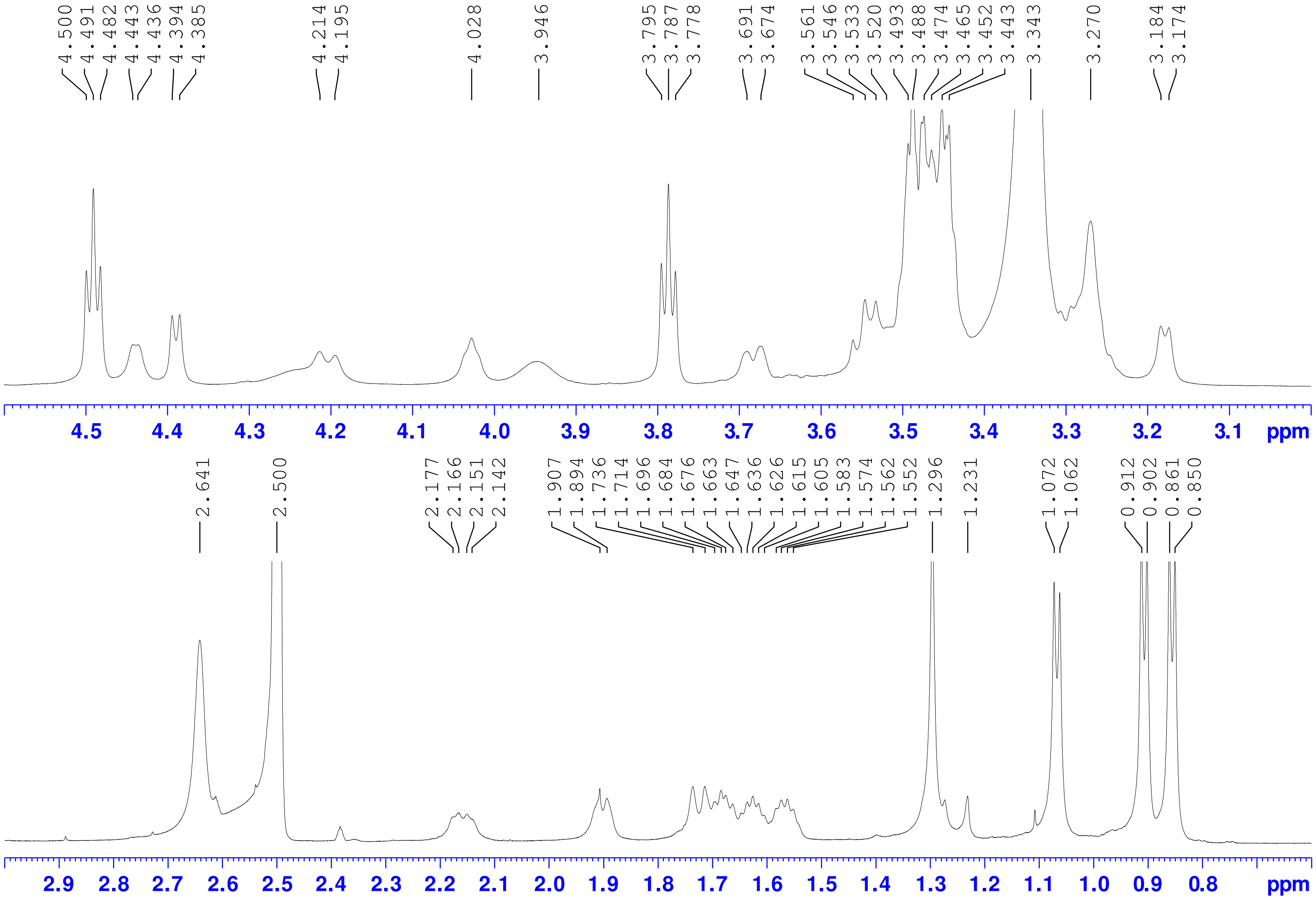

Figure S45. ^{1}H NMR (600 MHz, DMSO-d_6) of vanco-3PEG-Tz-NBD **9**.

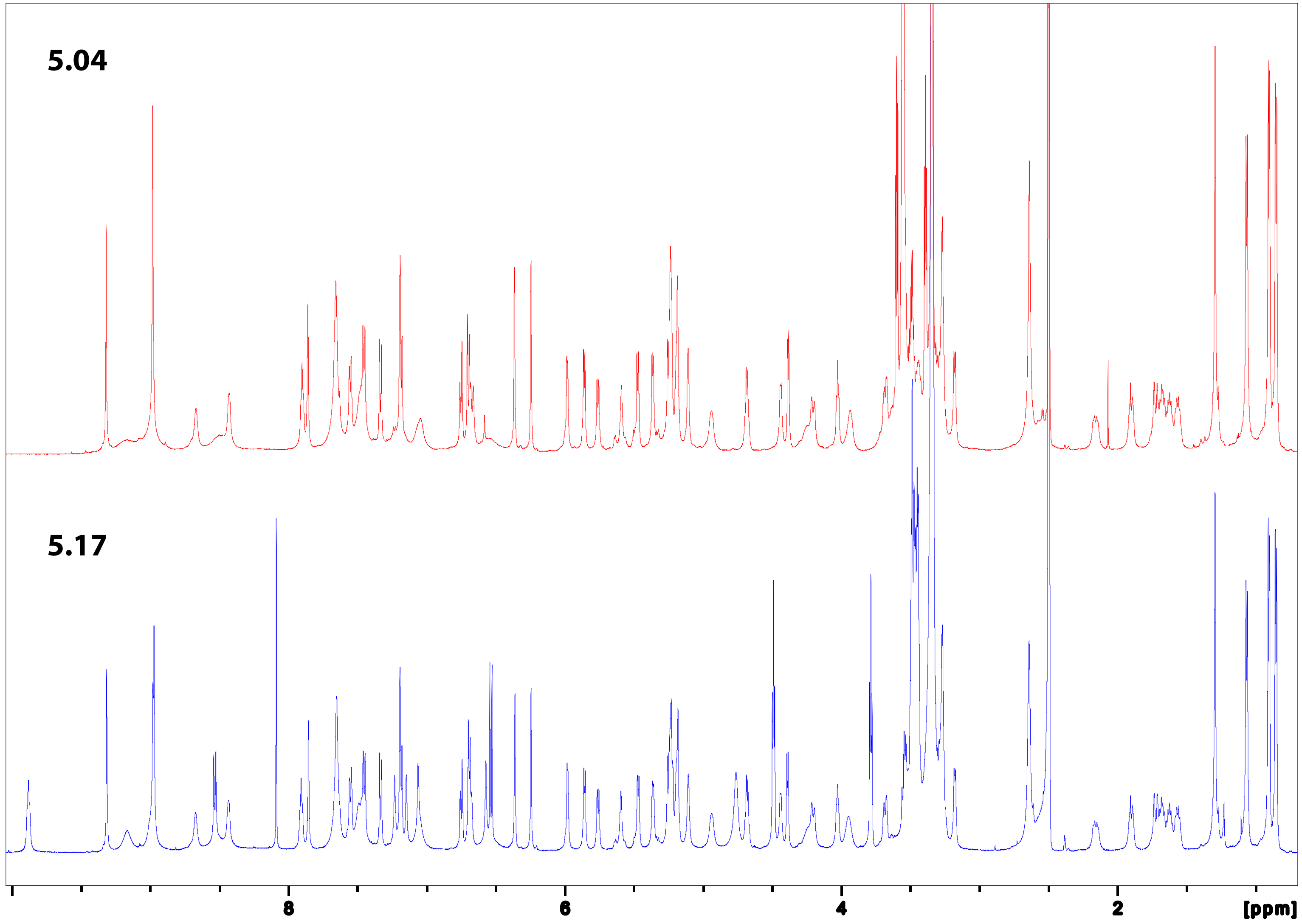

Figure S46. ^{1}H NMR (600 MHz, DMSO-d_6) of vanco-3PEG-Tz-NBD **9** (blue) *vs* vanco-3PEG-N_3 **4** (red).

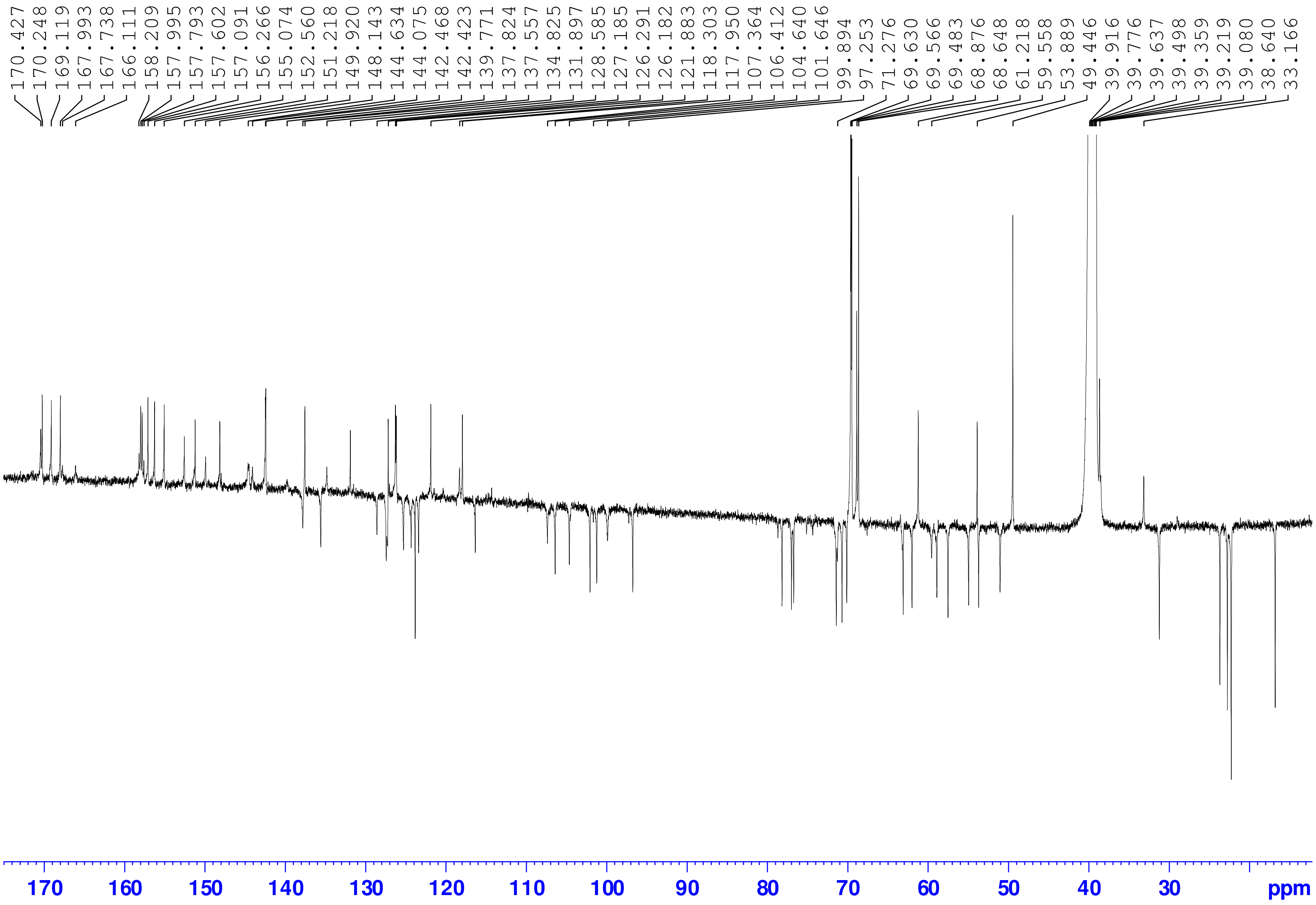

Figure S47. JMOD NMR (150 MHz, DMSO-d_6) of vanco-3PEG-Tz-NBD **9**.

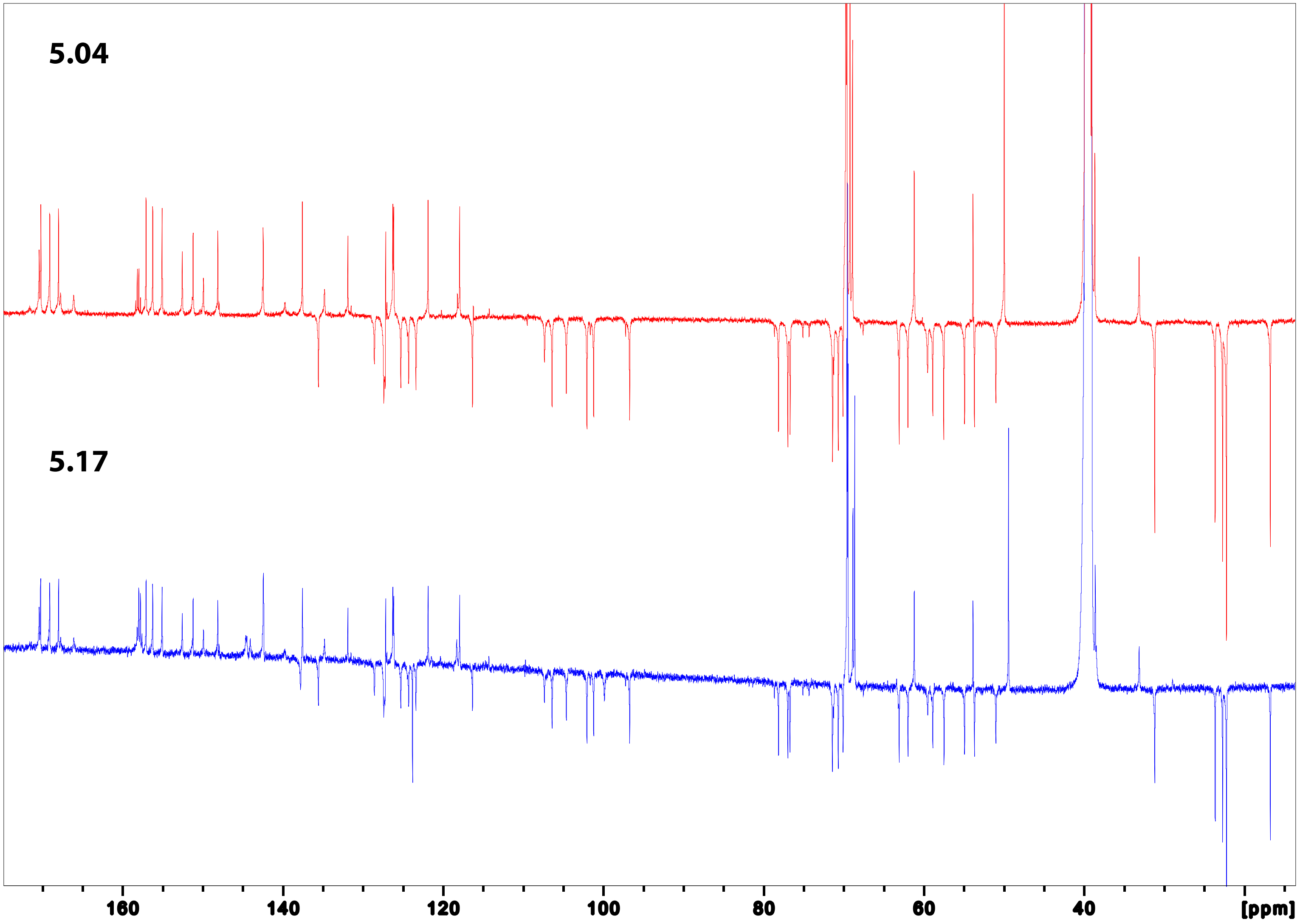

Figure S48. JMOD NMR (150 MHz, DMSO-d_6) of vanco-3PEG-Tz-NBD **9** (blue) *vs* vanco-3PEG-N_3 **4** (red).

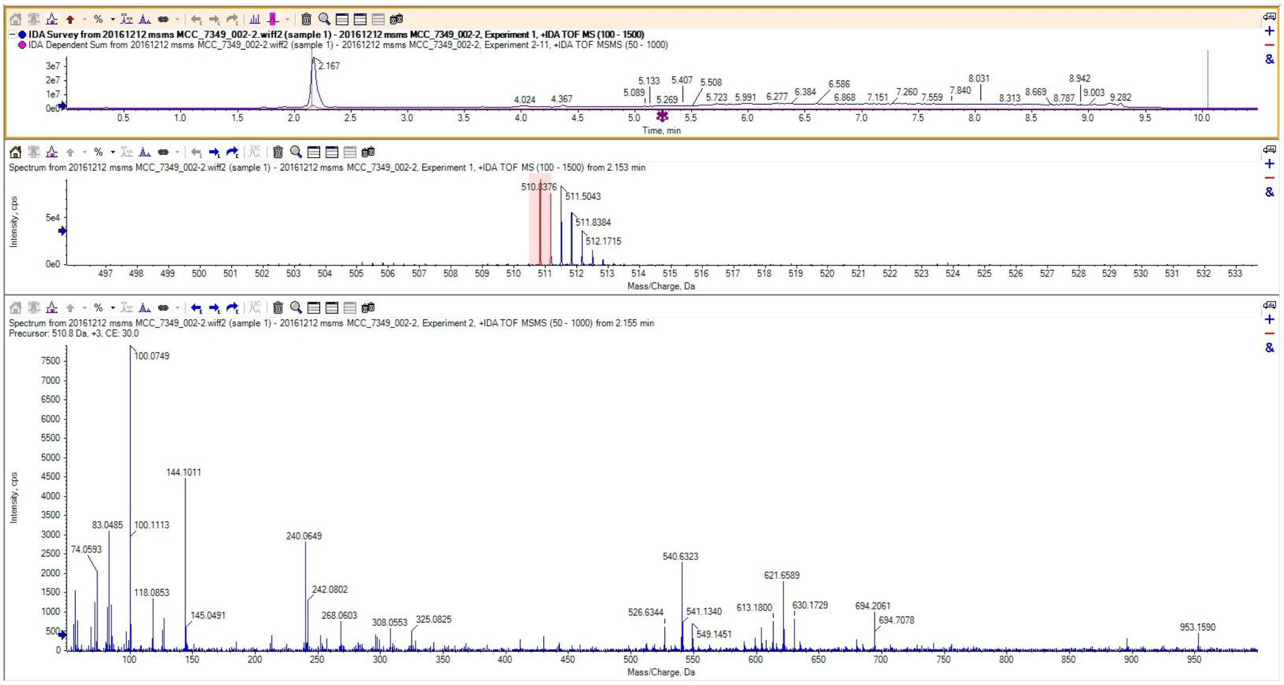

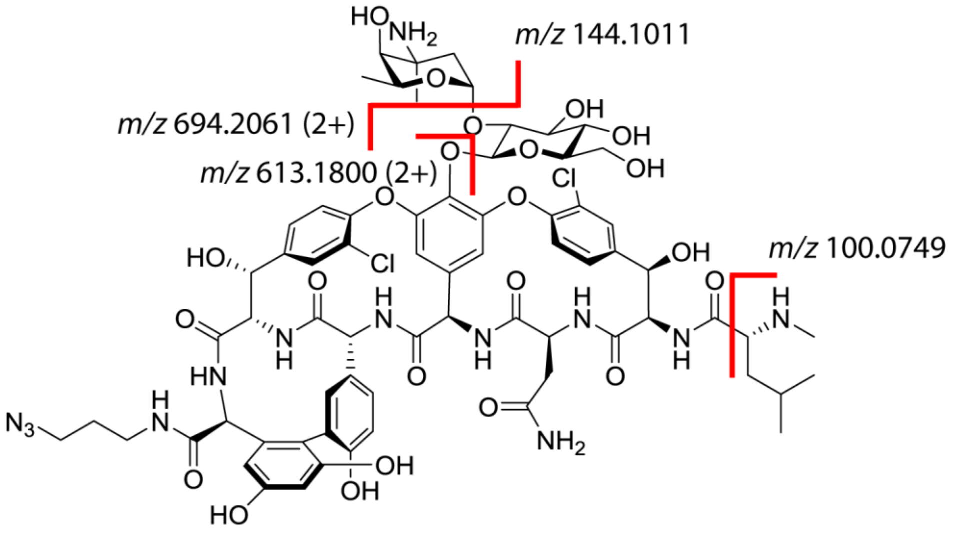

Figure S49. (+)-ESI-TOF-MS/MS of vanco-3C-N_3 **2.** Red lines indicate fragmentation patterns.

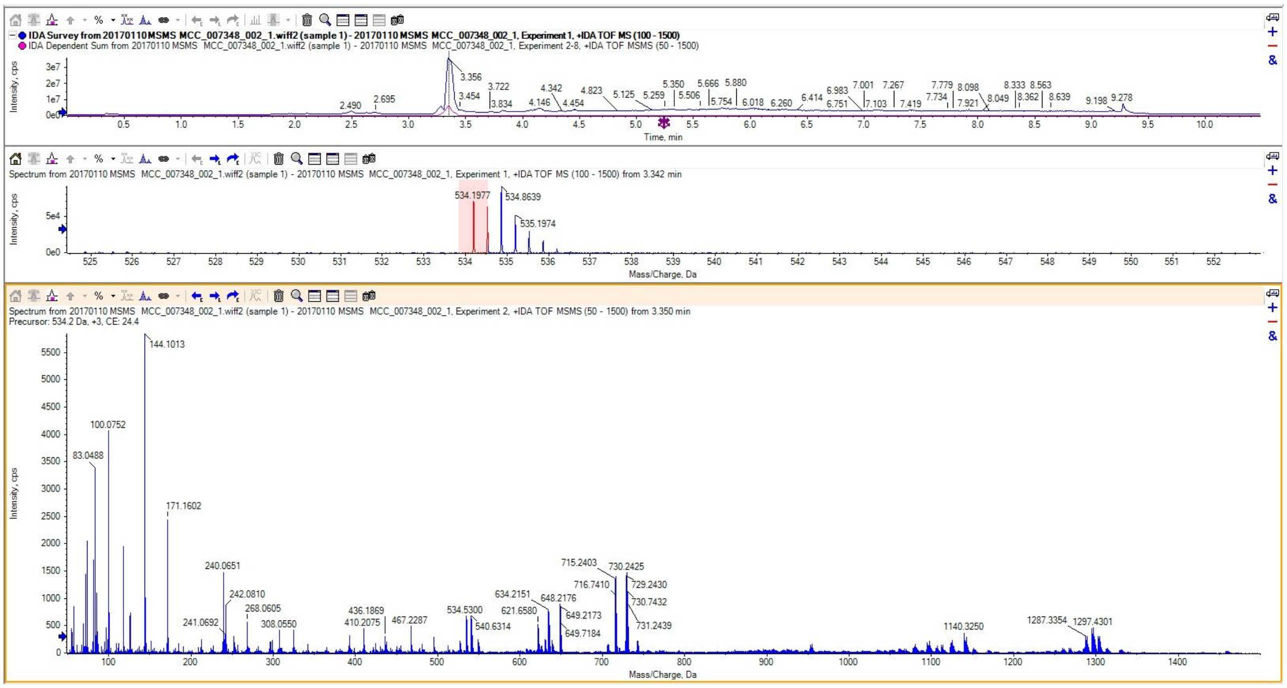

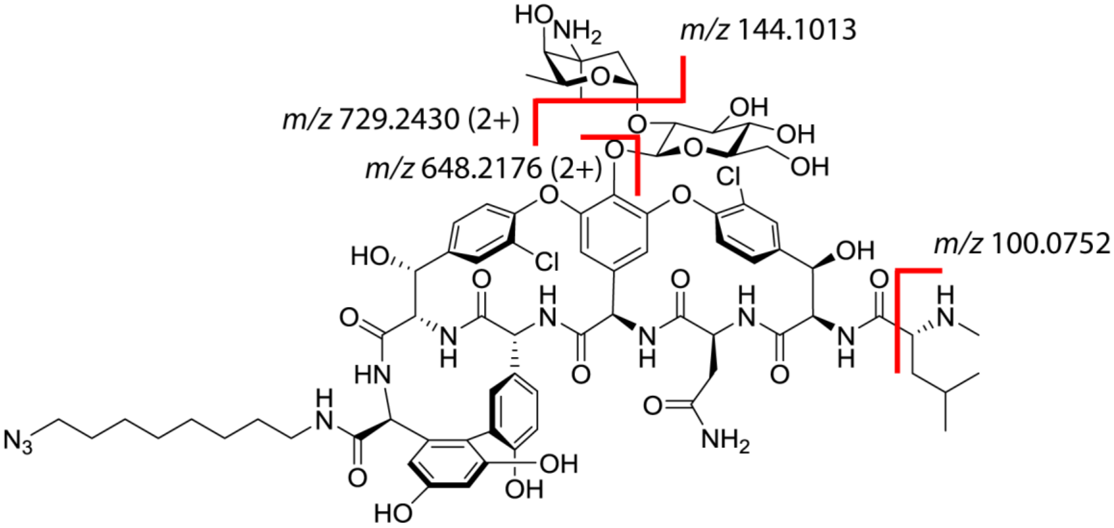

Figure S50. (+)-ESI-TOF-MS/MS of vanco-8C-N_3 **3**. Red lines indicate fragmentation patterns.

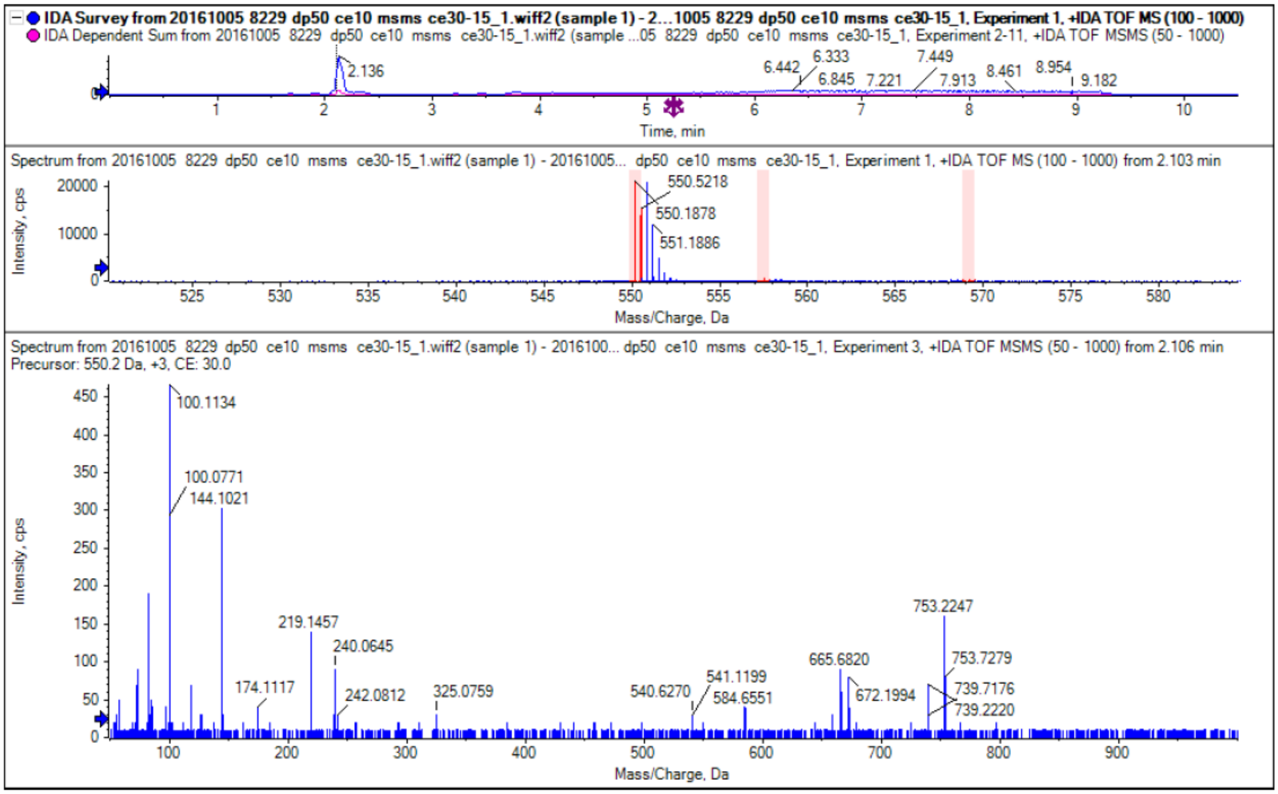

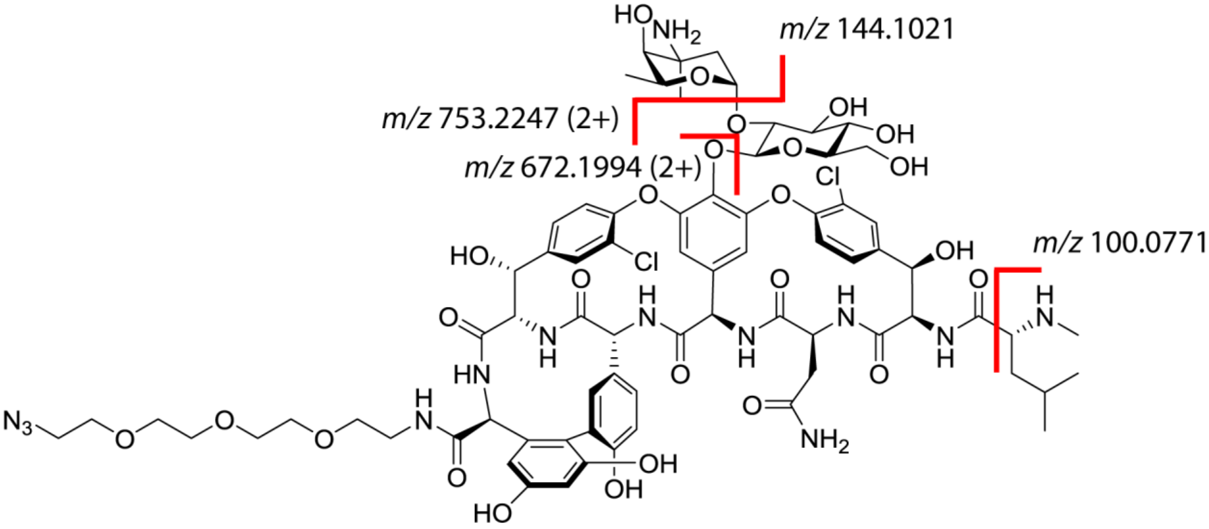

Figure S51. (+)-ESI-TOF-MS/MS of vanco-3PEG-N_3 **4**. Red lines indicate fragmentation patterns.

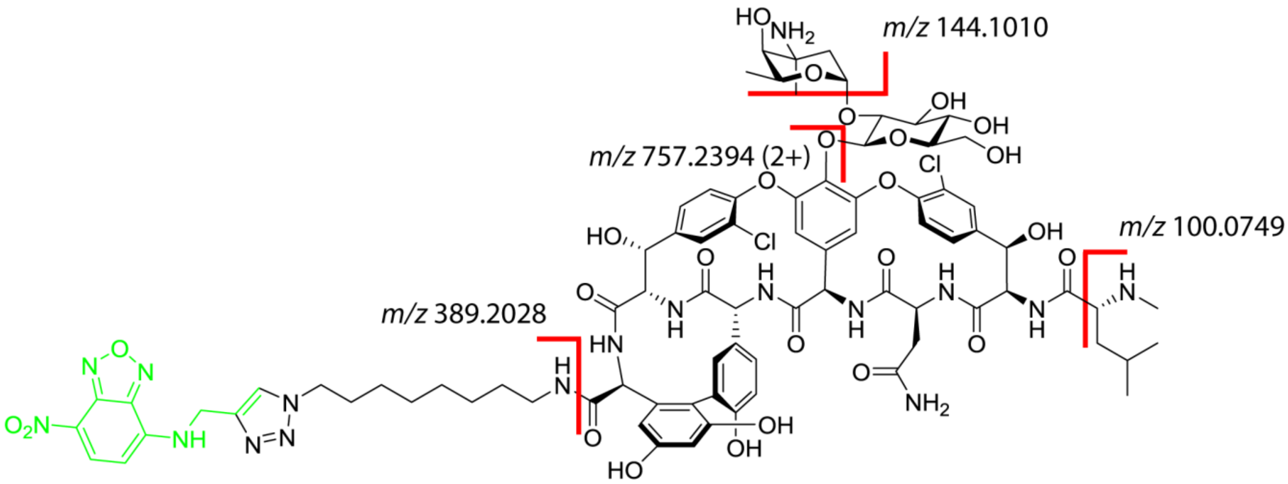

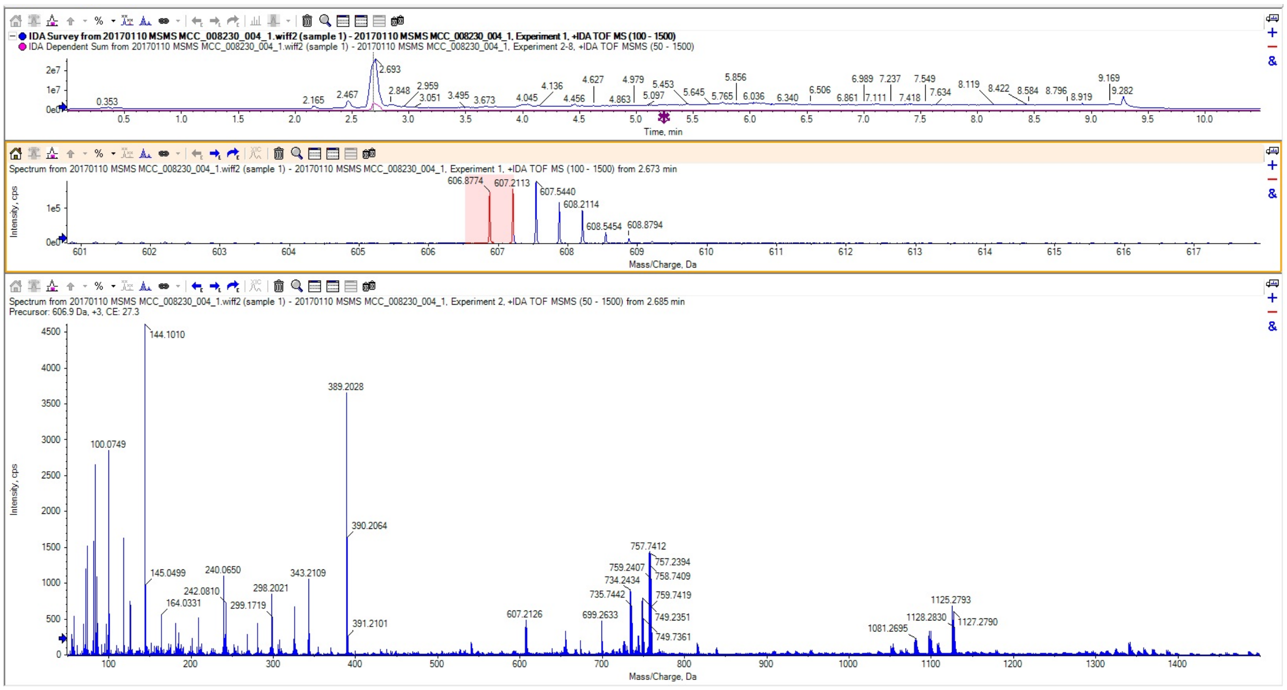

Figure S52. (+)-ESI-TOF-MS/MS of vanco-8C-Tz-NBD **7.** Red lines indicate fragmentation patterns, green structure is the NBD fluorophore.

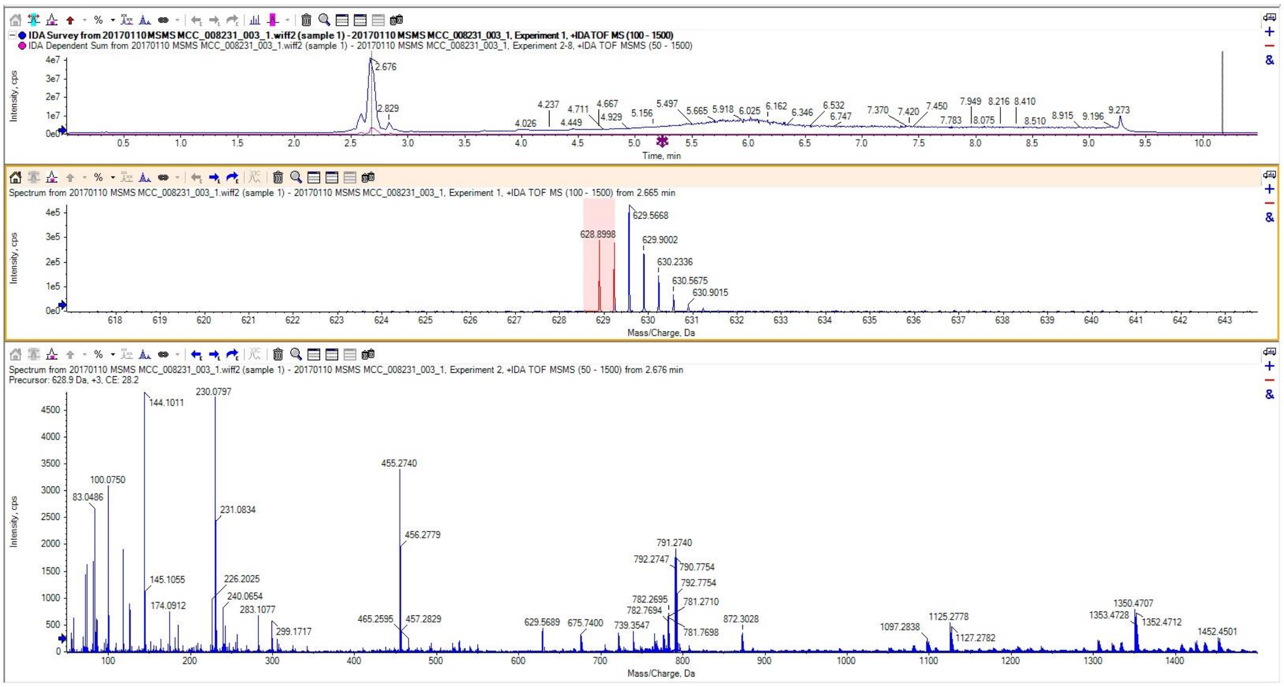

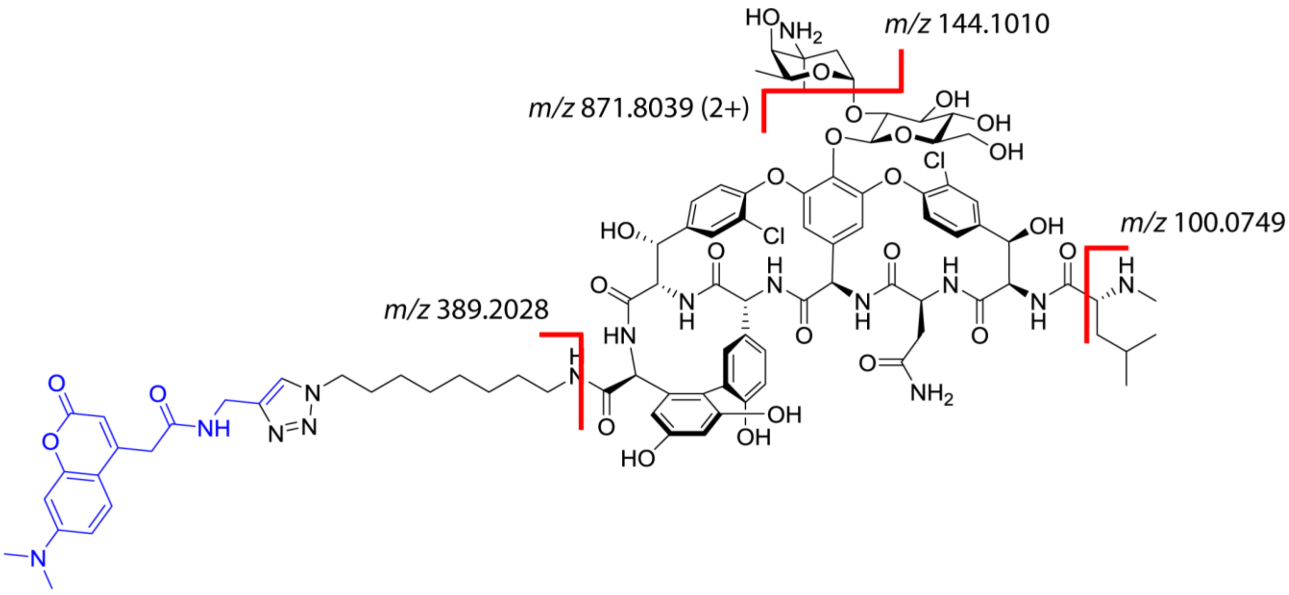

Figure S53. (+)-ESI-TOF-MS/MS of vanco-8C-Tz-DMACA **8**. Red lines indicate fragmentation patterns, blue structure is the DMACA fluorophore.

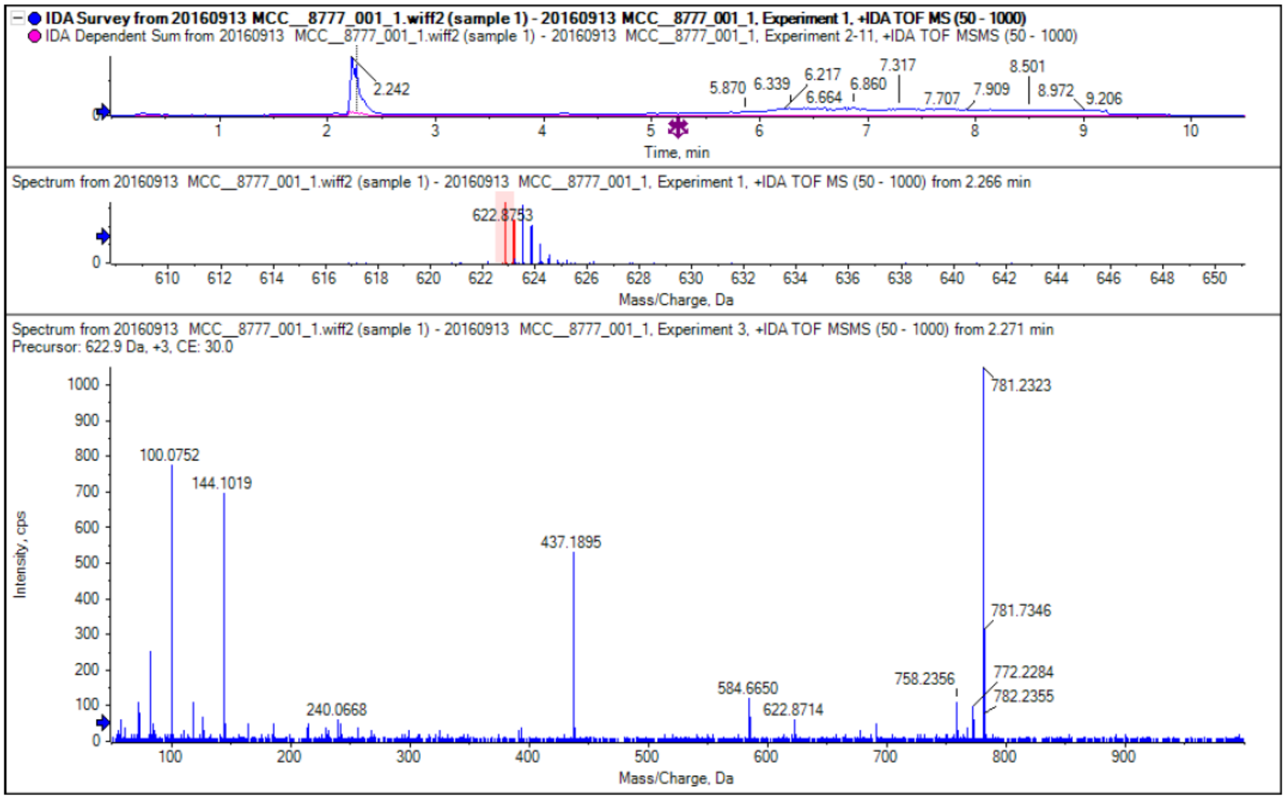

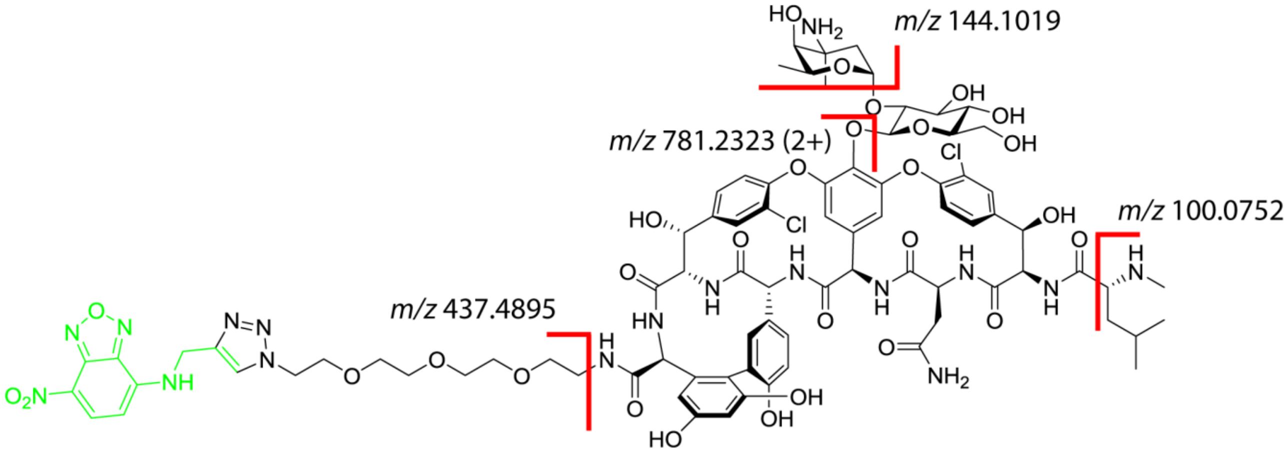

Figure S54. (+)-ESI-TOF-MS/MS of vanco-3PEG-Tz-NBD **9**. Red lines indicate fragmentation patterns, green structure is the NBD fluorophore.

Table S1. Selectivity ratio for *Staphylococcus aureus* strains over *S. aureus* ATCC 25923.

S. aureus strains[a]	Probe 9 Selectivity vs *S. aureus* ATCC 25923			
	1 μg/mL	8 μg/mL	16 μg/mL	64 μg/mL
ATCC 25923, Control	1	1	1	1
ATCC 43300, MRSA	1.38	1.17	1.13	1.14
Clinical isolate, MRSA, DRSA	4.27	4.58	4.34	5.09
Clinical isolate, MRSA	1.10	1.08	1.27	1.43
NRS 17, GISA	1.70	2.68	2.77	3.53
NRS 1, GISA	9.90	13.74	17.55	18.07
VRS 3b, VRSA	0.25	0.09	0.12	0.17
VRS 4, VRSA	0.14	0.05	0.06	0.08
VRS 1, VRSA	0.04	0.01	0.01	0.02
VRS 10, VRSA	0.27	0.09	0.11	0.15

[a]Full strain descriptions detailed in **Table S4.**

Table S2. Antimicrobial activity of antibiotics against *Escherichia coli* strains[a].

Compound	*E. coli* MIC (µg/mL)							
	ATCC 25922 FDA Control		MB4827 K12[b]		CGSC7139 DC2 mutant		MB4902 LpxC mutant	
	MHB	CaMHB	MHB	CaMHB	MHB	CaMHB	MHB	CaMHB
Polymyxin B sulfate	0.125	0.125	0.125	0.125	0.125	0.125	0.03–0.06	0.03–0.06
Colistin sulfate	0.25	0.25	0.06–0.125	0.06–0.25	0.125	0.125–0.25	0.03–0.06	0.03–0.06
Octapeptin C4	4	4	2	2	1–2	2–8	2	2
Tachyplesin-1	2–4	2–8	2–4	2–4	2–8	2–8	2	1–2
Arenicin-3	2	1–4	1–4	2–4	2–8	2–8	1–2	1
Gentamicin sulfate	2	1	0.5-1	0.5–1	0.5–4	0.5–2	0.25–0.5	0.25–0.5
Trimethoprim	1	1-2	0.5	0.5	0.25–0.5	0.25–0.5	0.5–1	0.5–1
Citropin 1.1	32	64	32-64	>64	64	>64	16	64
Erythromycin	64	64	64	>64	>64	>64	8	8

[a]MIC (minimum inhibitory concentration) was tested in 96-well micro-titre plates (non-treated polystyrene plate, flat bottom). [b]Parent strain for LpxC mutant.

Table S3. Comparison of antimicrobial activity of probe 7, probe 9 and commercial fluorescent probes[a].

Compound	MIC (µg/mL)				
	S. aureus				*E. coli*
	ATCC 25923 Control	ATCC 43300 MRSA	NRS 1 GISA, MRSA	VRS 1 NARSA	ATCC 29522
1 Vancomycin	2	1	8	>64	>64
7 Van-8C-Tz-NBD	1	0.5	4	>64	>64
8 Van-8C-Tz-DMACA	1	1	4	>64	>64
9 Van-3PEG-Tz-NBD	2	2	8	>64	>64
9 Van-3PEG-Tz-NBD (one year old stock)	2	2	8	>64	>64
Van-FITC	>32	>32	>32	>64	>64
Van-BODIPY	2	1	8	>64	>64

[a]All compounds were tested side by side in triplicate. This experiment was independently performed from **Table 1**, therefore shows some variation in MIC (minimum inhibitory concentration) results for compounds reported in both tables.

Table S4. The strains used in this study.

Strain		Strain description	Source
Escherichia coli	ATCC 25922	FDA control	American Type Culture Collection (ATCC)
	ATCC 700928 (CFT073)	Parent strain for *E. coli* WaaL mutant	
	MB4827	K12; parent strain for *E. coli lpxC* mutant	Merck
	MB4902	*lpxC* mutant	
	CGSC7139	DC2 mutant	The *E. coli* Genetic Stock Center (CGSC)
	Mutant of CFT073	WaaL mutant	Schembri, M. A.[1]
	BW25113	Genotype: F- Δ(*araD-araB*)*567* Δ*lacZ4787*::rrnB-3 λ- *rph-1* Δ(*rhaD-rhaB*)*568 hsdR514*	Wanner, B. L.[2]
Klebsiella pneumoniae	ATCC 700603	Multidrug-resistant (MDR); extended spectrum β-lactamase (ESBL) producer	ATCC
Pseudomonas aeruginosa	ATCC 27853	QC strain	ATCC
Staphylococcus aureus	clinical isolate	Methicillin resistant *S. aureus* (MRSA)	Clinical isolate - Australia
	clinical isolate	MRSA, daptomycin resistant *S. aureus* (DRSA)	
	NRS 17	MRSA (mecA), Glycopeptide intermediate *S. aureus* (GISA)	Network on Antimicrobial Resistance in *Staphylococcus aureus* (NARSA)*
	NRS 1 (Mu50)	MRSA (SCC*mec*: type II), GISA	
	VRS 3b	MRSA (mecA), vancomycin resistant *S. aureus* (VRSA) (VanA)	
	VRS 10	VRSA (VanA)	
	VRS 4	MRSA (mecA), VRSA(VanA)	
	VRS 1	MRSA (mecA), VRSA (VanA, Tn1546)	
	ATCC 43300	MRSA (SCC*mec*: type II)	ATCC
	ATCC 25923	seattle 1945, sensitive QC strain control	
Streptococcus pneumoniae	ATCC 700677	MDR	ATCC
	ATCC 33400	Type strain	
Staphylococcus epidermidis	ATCC 12228	FDA strain PCI 1200	ATCC
Bacillus subtilis	ATCC 6633	QC strain for food testing	ATCC
Enterococcus faecalis	ATCC 29212	Control strain	ATCC

Strain		Strain description	Source
Enterococcus faecium	clinical isolate	vancomycin resistant Enterococcus (VRE), VanA	Clinical isolate - Australia
	ATCC 35667	Control strain	ATCC
	ATCC 51559	MDR, VRE (VanA)	

*NARSA strains were acquired via BEI Resources www.beiresources.org

REFERENCES

1. Sarkar, S.; Ulett, G. C.; Totsika, M.; Phan, M.-D.; Schembri, M. A., Role of capsule and O antigen in the virulence of uropathogenic *Escherichia coli*. *PloS one* **2014,** *9* (4), e94786.
2. Datsenko, K. A.; Wanner, B. L., One-step inactivation of chromosomal genes in *Escherichia coli* K-12 using PCR products. *Proceedings of the National Academy of Sciences* **2000,** *97* (12), 6640-6645.
