# Supplementary material for: Synthesis of vancomycin fluorescent probes that retain antimicrobial activity, identify Gram-positive bacteria, and detect Gram-negative outer membrane damage
Source: Commun Biol. 2023 Apr 14;6:409. doi: 10.1038/s42003-023-04745-x (PMC10102067; doi:10.1038/s42003-023-04745-x)
Supplement: Supplementary file 3 — Description of Additional Supplementary Data [file 42003_2023_4745_MOESM3_ESM.docx]

**Description of Additional Supplementary Files**

**File name:** Supplementary Data 1

**Description:** Tables S5-S8

**File name:** Supplementary Data 2

**Description:** The source data behind the graphs in the paper

**File name:** : Figure S3-S. aureus ATCC 700699 movie

**Description:** : Video of S. aureus ATCC 700699 labelled with probe 7.
