# Supplementary material for: Synthesis of vancomycin fluorescent probes that retain antimicrobial activity, identify Gram-positive bacteria, and detect Gram-negative outer membrane damage
Source: Commun Biol. 2023 Apr 14;6:409. doi: 10.1038/s42003-023-04745-x (PMC10102067; doi:10.1038/s42003-023-04745-x)
Supplement: Supplementary file 7 — Reporting Summary [file 42003_2023_4745_MOESM7_ESM.pdf]

nature portfolio

Corresponding author(s): Mark A. T. Blaskovich

Last updated by author(s): Feb 20, 2023

Reporting Summary

Nature Portfolio wishes to improve the reproducibility of the work that we publish. This form provides structure for consistency and transparency in reporting. For further information on Nature Portfolio policies, see our Editorial Policies and the Editorial Policy Checklist.

Statistics

For all statistical analyses, confirm that the following items are present in the figure legend, table legend, main text, or Methods section.

n/a	Confirmed	
☐	☒	The exact sample size (*n*) for each experimental group/condition, given as a discrete number and unit of measurement
☐	☒	A statement on whether measurements were taken from distinct samples or whether the same sample was measured repeatedly
☐	☒	The statistical test(s) used AND whether they are one- or two-sided<br>*Only common tests should be described solely by name; describe more complex techniques in the Methods section.*
☒	☐	A description of all covariates tested
☐	☒	A description of any assumptions or corrections, such as tests of normality and adjustment for multiple comparisons
☐	☒	A full description of the statistical parameters including central tendency (e.g. means) or other basic estimates (e.g. regression coefficient) AND variation (e.g. standard deviation) or associated estimates of uncertainty (e.g. confidence intervals)
☐	☒	For null hypothesis testing, the test statistic (e.g. F, t, r) with confidence intervals, effect sizes, degrees of freedom and P value noted<br>*Give P values as exact values whenever suitable.*
☒	☐	For Bayesian analysis, information on the choice of priors and Markov chain Monte Carlo settings
☒	☐	For hierarchical and complex designs, identification of the appropriate level for tests and full reporting of outcomes
☒	☐	Estimates of effect sizes (e.g. Cohen's d, Pearson's r), indicating how they were calculated

Our web collection on statistics for biologists contains articles on many of the points above.

Software and code

Policy information about availability of computer code

Data collection	CytExpert 2.3<br>BD FACSDiva Software v8.0.1
Data analysis	Fiji (Image J), Kaluza Analysis 1.3 and 2.1 software<br>GraphPad Prism 8, Microsoft Excel,

For manuscripts utilizing custom algorithms or software that are central to the research but not yet described in published literature, software must be made available to editors and reviewers. We strongly encourage code deposition in a community repository (e.g. GitHub). See the Nature Portfolio guidelines for submitting code & software for further information.

Data

Policy information about availability of data

All manuscripts must include a data availability statement. This statement should provide the following information, where applicable:

- Accession codes, unique identifiers, or web links for publicly available datasets
- A description of any restrictions on data availability
- For clinical datasets or third party data, please ensure that the statement adheres to our policy

All relevant data are available in this article and its Supplementary Information files, except for original image files and data underlying the graphs, which are available from the corresponding author upon reasonable request.

Human research participants

Policy information about studies involving human research participants and Sex and Gender in Research.

Reporting on sex and gender	N/A
Population characteristics	N/A
Recruitment	N/A
Ethics oversight	N/A

Note that full information on the approval of the study protocol must also be provided in the manuscript.

Field-specific reporting

Please select the one below that is the best fit for your research. If you are not sure, read the appropriate sections before making your selection.

☒ Life sciences ☐ Behavioural & social sciences ☐ Ecological, evolutionary & environmental sciences

For a reference copy of the document with all sections, see nature.com/documents/nr-reporting-summary-flat.pdf

Life sciences study design

All studies must disclose on these points even when the disclosure is negative.

Sample size	A sample size of at least 2 biological replicates was used to determine statistical significance between tested groups.
Data exclusions	No data was excluded
Replication	All studies conducted at the University of Queensland and University of Exeter were repeated at minimum of 2 biological replicates. Using the described methodology, all attempts at replication were successful.
Randomization	NRandomization is not relevant to our studies as no population, clinical data or field studies where randomization is required was carried out. All work was direct experimental design and all output was used in each set of analysis.
Blinding	N/A

Reporting for specific materials, systems and methods

We require information from authors about some types of materials, experimental systems and methods used in many studies. Here, indicate whether each material, system or method listed is relevant to your study. If you are not sure if a list item applies to your research, read the appropriate section before selecting a response.

Materials & experimental systems

n/a	Involved in the study	
☒	☐	Antibodies
☒	☐	Eukaryotic cell lines
☒	☐	Palaeontology and archaeology
☒	☐	Animals and other organisms
☒	☐	Clinical data
☒	☐	Dual use research of concern

Methods

n/a	Involved in the study	
☒	☐	ChIP-seq
☐	☒	Flow cytometry
☒	☐	MRI-based neuroimaging

Flow Cytometry

Plots

Confirm that:

☒ The axis labels state the marker and fluorochrome used (e.g. CD4-FITC).

☒ The axis scales are clearly visible. Include numbers along axes only for bottom left plot of group (a 'group' is an analysis of identical markers).

☒ All plots are contour plots with outliers or pseudocolor plots.

☒ A numerical value for number of cells or percentage (with statistics) is provided.

Methodology

Sample preparation	Bacteria were cultured in LB at 37 °C overnight. A sample of each culture was then diluted 50-fold in LB and incubated at 37 °C for 1.5–2 h. The resultant mid-log phase cultures were harvested at 4000 rpm for 25 min, washed once with HBSS (4000 rpm, for 15 min), and resuspended in HBSS to an OD600 of 2. Bacteria were treated with vancomycin probes at a concentration of 16 µg/mL in HBSS (37 °C, 30 min) and washed one time with 1 mL of HBSS. Bacteria cell pellets were resuspended in 1 mL of HBSS. Fluorescence intensity was measured using the flow cytometer.<br>In terms of selectivity test in a bunch of Gram-positive and Gram-negative bacteria with the fluorescent probe 9. Bacteria were cultured in LB at 37 °C overnight. A sample of each culture was then diluted 40-fold in fresh LB and incubated at 37 °C for 1.5-2 h (OD=0.4-0.6). The resultant mid-log phase cultures were harvested and washed once with HBSS, and resuspended in HBSS to an OD600 of 1. Then 1 mL of the cultures were transferred to Eppendorf tubes and centrifuged. A solution (500 µL) of probe 9 (32 µg /mL) was added to an Eppendorf tube containing bacteria pellets, left for 30 min at 37 °C with shaking (180 rpm). Bacteria were washed again with HBSS, then a solution (500 µL) of SYTO® 60 (5 µM in HBSS) was added and left for 10 min on ice. Bacteria were washed once with HBSS and the pellets were suspended and then diluted in HBSS for flow cytometer measurement.
Instrument	Gallios flow cytometer from Beckman Coulter; Cytoflex S, Beckman Coulter, Inc. 250 S. Kraemer Blvd. Brea, CA 92821 U.S.A.
Software	CytExpert 2.3<br>BD FACSDiva Software v8.0.1<br>Kaluza Analysis 1.3 software<br>Kaluza Analysis 2.1 software
Cell population abundance	No cell sorting was performed in this work.
Gating strategy	Cytoflex S: The detectors used here were FITC (Excitation 488 nm; Emission 525/40 nm) and APC (Excitation 638 nm; Emission 660/10 nm). 40000 events were collected. Flow cytometer Gallios from Beckman Coulter: 10000 events were collected. Fluorescent intensity from FL1 (Excitation 488 nm; Emission 525/20 nm) and FL9 (Excitation 405 nm; Emission 450/50 nm) were plotted against the number of events count. Positive events were gated on the histogram to estimate the mean fluorescent intensity (MFI). Forward and side scatter gating were used to identify the cells of interest based on the relative size and complexity of the cells, while removing debris and other events that are not of interest. To achieve high-resolution detection, the instrument detection gain was set to side scatter (SSC) 300, forward scatter (FSC) 50, and manual threshold for SSC in 3000 (height) (Figure2C).

☒ Tick this box to confirm that a figure exemplifying the gating strategy is provided in the Supplementary Information.
